# Origin and evolution of the major histocompatibility complex class I region in eutherian mammals

**DOI:** 10.1002/ece3.5373

**Published:** 2019-06-14

**Authors:** Shamshidin Abduriyim, Da‐Hu Zou, Huabin Zhao

**Affiliations:** ^1^ Department of Ecology, Hubei Key Laboratory of Cell Homeostasis, College of Life Science Wuhan University Wuhan China

**Keywords:** bats, comparative genomics, evolution, mammals, MHC, origin

## Abstract

Major histocompatibility complex (MHC) genes in vertebrates are vital in defending against pathogenic infections. To gain new insights into the evolution of MHC Class I (MHCI) genes and test competing hypotheses on the origin of the MHCI region in eutherian mammals, we studied available genome assemblies of nine species in Afrotheria, Xenarthra, and Laurasiatheria, and successfully characterized the MHCI region in six species. The following numbers of putatively functional genes were detected: in the elephant, four, one, and eight in the extended class I region, and κ and β duplication blocks, respectively; in the tenrec, one in the κ duplication block; and in the four bat species, one or two in the β duplication block. Our results indicate that MHCI genes in the κ and β duplication blocks may have originated in the common ancestor of eutherian mammals. In the elephant, tenrec, and all four bats, some MHCI genes occurred outside the MHCI region, suggesting that eutherians may have a more complex MHCI genomic organization than previously thought. Bat‐specific three‐ or five‐amino‐acid insertions were detected in the MHCI α1 domain in all four bats studied, suggesting that pathogen defense in bats relies on MHCIs having a wider peptide‐binding groove, as previously assayed by a bat MHCI gene with a three‐amino‐acid insertion showing a larger peptide repertoire than in other mammals. Our study adds to knowledge on the diversity of eutherian MHCI genes, which may have been shaped in a taxon‐specific manner.

## 1 INTRODUCTION

The major histocompatibility complex (MHC) is an extremely important component of the vertebrate genome due to the vital roles of the proteins it encodes, including the processing and presentation of self‐ and foreign peptides involved in adaptive and innate immunity against pathogenic infection, and in autoimmunity (Cresswell, 2005; Warrens & Lechler, 1999). In particular, MHC class I (MHCI) and II (MHCII) genes encode proteins that play a pivotal role in the adaptive immune system. While the proteins of both classes are similar in structure and function, there are also differences (see Kaufman, Salomonsen, & Flajnik, 1994). While both classes of proteins present peptides on the cell surface for recognition by T cells, MHCI antigens bind endogenous peptides to form peptide‐MHCI complexes, which are then presented on nucleated cells and recognized by cytotoxic CD8^+^ T lymphocytes. In contrast, MHC class II proteins bind exogenous peptides to form peptide‐MHCII complexes, which are presented on cells such as dendritic cells, macrophages, or B cells and activate CD4^+^ helper T cells, leading to subsequent coordination and regulation of effector cells **(**Cresswell, 2005; Kaufman et al., 1994; Shiina, Blancher, Inoko, & Kulski, 2017; Wieczorek et al., 2017). Although both MHCI and MHCII class molecules present antigens to T‐cell receptors, some MHCI genes interact with receptors in the vomeronasal organ; are involved in mating choice and kin recognition; affect nervous system development and plasticity, synaptic function, and behavior (reviewed in Shiina et al., 2017).

The MHC region, in which the MHCI and MHCII genes are the core components, is typically the most gene‐dense and polymorphic region in the genome—for example, MHCI *HLA‐B* is the most polymorphic gene in the human genome (Mungall et al., 2003). Polymorphism in MHCI and MHCII genes can be explained by host–pathogen coevolution (Borghans, Beltman, & De Boer, 2004) and accordingly provides an ideal means of assessing the immunological fitness of a population and/or species in terms of ability to respond to diseases (Sommer, 2005). Since its discovery in the mouse more than 80 years ago (Gorer, 1936), the MHC, and especially MHCI, has become one of the most intensively studied regions in the vertebrate genome (Abduriyim et al., 2017, 2019; Deakin et al., 2006; Minias, Pikus, & Anderwald, 2019; Mungall et al., 2003; Ng et al., 2016).

**Figure 1:**
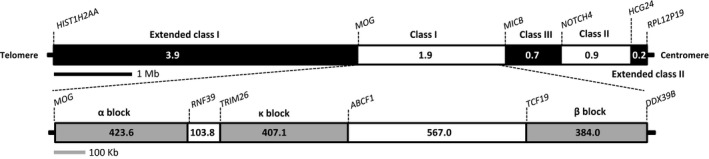
Simplified map of the human MHC (upper) and MHC class I (lower) regions. The MHC was drawn according to Horton et al. (2004). Both regions are drawn to scale. The α duplication lies between the *MOG* (myelin oligodendrocyte glycoprotein) and *RNF39* (RING finger protein 39) genes, the κ duplication block between *TRIM26* (tripartite motif containing 26) and *ABCF1* (ATP‐binding cassette subfamily F member 1), and the β duplication block between *TCF19* (transcription factor 19) and *MICB*/*BAT1*

The classical MHC region in mammals, particularly eutherian mammals, comprises class I, II, and III clusters (Beck et al., 1999; Kumánovics, Takada, & Lindahl, 2003). Later on, large‐scale studies extended this region, due to its content in relation to paralogy, polymorphism, immune function, and disease (Horton et al., 2004; Stephens et al., 1999). This extended region is on the short arm of human chromosome 6 (Figure 1); spans about 7.6 Mb, from the histone H2A type 1‐A (*HIST1H2AA*) gene to ribosomal protein L12 pseudogene 19 (*RPL12P19*); and is further divided into five subregions, including extended class I, class I, class III, class II, and extended class II (Horton et al., 2004; Shiina et al., 2017). Of them, MHCI consists of three duplicated‐block regions separated by two framework regions (Figure 1), which are highly conserved in gene content and order among mammals, but are absent from nonmammalian vertebrates (Belov et al., 2006; Kulski, Shiina, Anzai, Kohara, & Inoko, 2002).

MHCI genes within the canonical MHCI region (CMR) are distributed in three duplication blocks, termed (in order) α, κ, and β (Figure 1) (Dawkins et al., 1999; Kulski et al., 2002). The α block is bounded by *MOG* and *RNF39*, the κ block by *TRIM26* and *ABCF1*, and the β block by *TCF19* and *MICB*/*BAT1* (Figure 1) (Belov et al., 2006; Dawkins et al., 1999; Kulski et al., 2002). The MHCI gene organization just described is likely specific to eutherian mammals (Kumánovics et al., 2003; Ng et al., 2016; Shiina et al., 2017). In contrast, in fishes, amphibians, birds, and basal mammals such as the opossum and platypus, MHCI genes are interspersed with class II genes in the class II region (Belov et al., 2006; Dohm, Tsend‐Ayush, Reinhardt, Grützner, & Himmelbauer, 2007; Kaufman, 2018; Michalova, Murray, Sultmann, & Klein, 2000; Ohta, Goetz, Hossain, Nonaka, & Flajnik, 2006).

**Figure 2:**
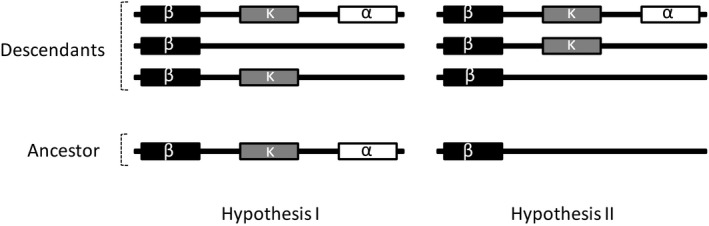
Two previous hypotheses for the evolution of canonical MHC class I region. Hypothesis I (Kumánovics et al., 2003) postulated that all of the three MHCI duplication blocks were present in the common ancestor of mammals, and were lost in taxon‐specific fashion, whereas hypothesis II (Ng et al., 2016) proposed that these duplication blocks arose in a stepwise manner, with the β duplication block originating first and the α block last

Improved MHC gene maps were generated over several decades for various vertebrate species, including eutherian mammals: human, *Homo sapiens*; chimpanzee, *Pan troglodytes*; rhesus macaque, *Macaca mulatta*; rat, *Rattus rattus*; mouse, *Mus musculus*; pig, *Sus scrupus*; horse, *Equus ferus*; sheep, *Ovis aries*; dog *Canis lupus familiaris*; cat, *Felis catus*; and bats (Beck et al., 2005; Chardon, Renard, & Vaiman, 1999; Gustafson et al., 2003; Kulski et al., 2002; Liu, Liu, Wang, & Ma, 2006; Ng et al., 2016; Renard et al., 2006; Shiina et al., 2017; Yuhki, Beck, Stephens, Neelam, & O'Brien, 2007). Eutherian mammals exhibit striking differences in MHCI structure and content from other vertebrates and basal mammals, suggesting that major evolutionary changes occurred between noneutherian and eutherian vertebrates. Two hypotheses have been proposed to explain the origin and evolution of eutherian mammal MHCI genes (Figure 2). Kumánovics et al. (2003) proposed that the MHCI genes in all three duplication blocks were present in the mammalian common ancestor, and some MHCI genes were subsequently lost in a taxon‐specific manner (Figure 2). Alternatively, Ng et al. (2016) proposed from studies on bats that the three duplication blocks arose in a stepwise fashion, with MHCI genes translocated first into the β block, then into the κ block, and finally into the α block (Figure 2).

Both of these hypotheses were based upon observations of MHCI gene organization in species from two of the four superorders in eutherian mammals (Laurasiatheria and Euarchontoglires), with a lack of species from the other two superorders—Xenarthra and Afrotheria (Kumánovics et al., 2003; Ng et al., 2016). To further understand the diversity and genomic distribution of MHCI genes in eutherian mammals and to test these two competing hypotheses, we analyzed the genomic sequences of nine species from Xenarthra, Afrotheria, and Laurasiatheria (Table 1). We included four bat genome sequences in our analysis, because bats rarely display clinical symptoms when infected by various viruses (Baker, Schountz, & Wang, 2013; Hayman, 2016), suggesting that the MHCI genes in bats may show unique immunological features that allow the bats to act as asymptomatic viral reservoirs. Our study adds to knowledge of the diversity of MHCI genes, and the complexity and origin of MHCI genomic structure in eutherian mammals. It also provides valuable genomic information relevant to studies in conservation genetics and evolutionary ecology based on MHCI genes.

## 2 MATERIALS AND METHODS

### 2.1 Genome data

We retrieved the genome assemblies of nine species of eutherian mammals from NCBI (National Center for Biotechnology Information, https://www.ncbi.nlm.nih.gov/, last accessed November 20, 2018). These species were the African bush elephant (*Loxodonta africana*) and lesser hedgehog tenrec (*Echinops telfairi*) in Afrotheria; the giant anteater (*Myrmecophaga tridactyla*) in Xenarthra; and the killer whale (*Orcinus orca*), Sunda pangolin (*Manis javanica*), common vampire bat (*Desmodus rotundus*), Natal long‐fingered bat (*Miniopterus natalensis*), great roundleaf bat (*Hipposideros armiger*), and Chinese horseshoe bat (*Rhinolophus sinicus*) in Laurasiatheria (Table 1). Detailed information for each genome assembly was presented in Table S1.

### 2.2 Identification of MHC class I genes

To obtain the MHCI repertoire for the target species, each genome sequence was analyzed by using an automatic pipeline developed in our laboratory, as described elsewhere (Feng, Zheng, Rossiter, Wang, & Zhao, 2014; Hong & Zhao, 2014; Jiao, Wang, Zhang, Jiang, & Zhao, 2018; Wang & Zhao, 2015). Briefly, we used full‐length MHCI protein sequences from the human, horse, and bats as queries in TBLASTN searches against each genome assembly, with a cutoff E‐value of 10^−10^. We then filtered redundant sequences that hit on the same genomic region and excluded hits shorter than 400 nucleotides, which is approximately one‐third of the complete length of an MHCI coding sequence. The remaining sequences were then further confirmed by nucleotide and protein BLAST searches at NCBI (https://blast.ncbi.nlm.nih.gov/Blast.cgi). Sequences with greater than 90% similarity to published MHC class I sequences were retained and further subjected to protein BLAST after translating nucleotides into protein sequences by MEGA7 (Kumar, Stecher, & Tamura, 2016), to preliminarily check their completeness and exons. Sequences with all six domain sequences (leader, extracellular domains α1, α2, α3, intercellular domain, and cytoplasmic tail; Bjorkman & Parham, 1990) were regarded as intact genes. Those with a continuous sequence longer than 400 nucleotides but lacking part of the coding region were considered to be partial. All confirmed sequences were then realigned by MACSE v2.03 (Ranwez, Douzery, Cambon, Chantret, & Delsuc, 2018), to discriminate presumably functional genes (PFGs) and pseudogenes. Among intact gene sequences, those with a putative start codon at the beginning of exon 1 and stop codon at the end of exon 6, and without deletions and/or insertions (indels; apart from multiples of three nucleotides) or premature stop codons in the open reading frame, were treated as PFGs; sequences not fulfilling these requirements were considered to be pseudogenes. Partial sequences containing no missing exons, indels, or premature stop codons were considered to be PFGs if the flanking regions of a given gene contained ambiguous nucleotides (Ns) that resulted from either incomplete genome sequencing or poor genome assembly. Otherwise, partial sequences were regarded as pseudogenes.

### 2.3 Identification of the canonical MHC class I region

To identify the canonical MHC class I region (CMR), we searched the genomic sequences for the six genes that demarcate the three duplication blocks (Figure 1). Steps to identify these six genes were the same as for the MHCI genes, but their protein sequences were used instead as queries. The scaffolds in which these genes were located were identified first, and the scaffolds that encompassed the MHCI and/or the six block‐demarcating genes were then retrieved from the genome sequences, separately for each species, before annotation.

To annotate the scaffolds extracted from each genome, all protein sequences annotated from the human, horse, shrew mouse (*Mus pahari*), and Natal long‐fingered bat (*M. natalensis*) genomes were used as queries, and all possible coding sequences (CDSs) were identified from the scaffolds using Genewise (Birney, Clamp, & Durbin, 2014). BLASTX searches (Altschul et al., 1997) were then executed at a cutoff E‐value of 10^−5^, using extracted CDSs as queries against the UniProt protein sequence database. Finally, we selected the best hit for each CDS and determined the gene name, location, and transcription direction.

### 2.4 Recombination analysis

Many studies involving various species have reported recombination events in MHC evolution (Abduriyim et al., 2017, 2019; Schaschl, Suchentrunk, Hammer, & Goodman, 2005). To minimize the impact of recombination on phylogenetic analysis (Arenas & Posada, 2010; Rousselle, Laverré, Figuet, Nabholz, & Galtier, 2019), a recombination analysis was performed by using RDP4 v. 4.97 (Martin, Murrell, Golden, Khoosal, & Muhire, 2015), following the advice of Martin, Murrell, Khoosal, and Muhire (2017), prior to subsequent analyses. Owing to the incompleteness of some sequences (Data S1), we focused on the region from exons 2 to 5, in order to include more MHCI sequences in our analyses. MHC sequences were first aligned by MUSCLE (Edgar, 2004), separately for each species. The most divergent sequences were excluded using the SDT v1.2 software (http://web.cbio.uct.ac.za/~brejnev/; distributed with RDP4), as suggested by Martin et al. (2017). Recombination detection was then run in RDP4 using the RDP, GENECONV, Bootscan, Maxchi, Chimaera, SiSscan, and 3Seq methods (Martin et al., 2015). Recombinants detected by at least three methods were further analyzed for recombination breakpoints by constructing a neighbor‐joining tree and checking with RECSCAN, RDP, Maxchi, 3Seq, and Chimaera plots. Finally, recombination events were rechecked using all methods available in RDP4, and those showed significant recombination signatures with at least four of the nine methods were regarded as significant recombinants. Given that our analytical regions were all shorter than 1,000 bp, we considered all recombination‐like events to be recombination, because recombination and gene conversion showed similar effects on sequences shorter than 1,000 bp (Richman, Herrera, Nash, & Schierup, 2003).

### 2.5 Reconstruction of MHCI gene repertoire evolution

In addition to the sequences derived in this study, our phylogenetic analysis also included the longest, most complete sequence for each of the MHCI genes in primates (human and rhesus macaque), rodents (mouse), carnivore (dog), even‐toed ungulate (pig), odd‐toed ungulate (horse), marsupial (opossum), downloaded from the NCBI GenBank. The accession number for each gene is given in the phylogenetic NeighborNet network. The data set was analyzed with a phylogenetic NeighborNet network, as a preferable alternative to phylogenetic trees in cases where gene duplication, recombination, and conversion likely occurred, implemented in SplitsTree v4.14.8 (Huson & Bryant, 2006) using the Jukes–Cantor method. A bootstrap analysis (Minh, Nguyen, & Haeseler, 2013) with 1,000 pseudo‐replicates was performed to investigate edge support across the network, with bootstrap values greater than 70 considered as strong support (Hillis & Bull, 1993). Sequences that showed significant recombination were excluded from the phylogenetic network reconstruction.

## 3 RESULTS

### 3.1 MHCI gene repertoire in eutherian mammals

In the genome assemblies of nine eutherian mammal species, the number of MHCI genes detected varied from three in the killer whale to 31 in the elephant (Table 1). The killer whale and pangolin had the lowest numbers of putatively functional genes (three and four PFGs, respectively), while the elephant had the highest number (19), with other species intermediate (six to 13; Table 1). We also found pseudogenes in all species studied except the killer whale, ranging from one in pangolin to 12 in elephant (Table 1). The four bat species were found to have nine to 19 MHCI genes, of which eight to 13 were PFGs and one to six were pseudogenes (Table 1). Nucleotide sequences of MHCI gene derived from genome sequences of these species in this study were presented in Data S1.

**Table 1:** Numbers of MHC class I gene repertoires derived from the genomes of nine eutherian mammals

Classification	Common name	Species name	Gene	PFG	Pseudogene	Full‐length gene	Partial gene	Gene^Scaffold^
Superorder Laurasiatheria								
Order Chiroptera								
Suborder Yangochiroptera	Common vampire bat	*Desmodus rotundus*	18	13	5	12	6	11^1^;4^1^;1^3^
	Natal long‐fingered bat	*Miniopterus natalensis*	9	8	1	1	8	2^1^;1^7^
Suborder Yinpterochiroptera	Great roundleaf bat	*Hipposideros armiger*	19	13	6	11	8	3^1^;2^3^;1^10^
	Chinese rufous horseshoe bat	*Rhinolophus sinicus*	15	13	2	3	12	3^1^;1^12^
Order Cetartiodactyla	Killer whale	*Orcinus orca*	3	3	0	3	0	2^1^;1^1^
Order Pholidota	Sunda pangolin	*Manis javanica*	5	4	1	3	2	1^5^
Superorder Xenarthra								
Order Pilosa	Giant anteater	*Myrmecophaga tridactyla*	12	10	2	5	7	1^12^
Superorder Afrotheria								
Order Afrosoricida	Lesser hedgehog tenrec	*Echinops telfairi*	8	6	2	4	4	3^1^;2^1^;1^3^
Order Proboscidea	African bush elephant	*Loxodonta africana*	31	19	12	18	13	7^1^;5^1^;4^2^;3^1^;2^1^;1^6^

Note

PFG is the abbreviation of putatively functional gene. Gene^Scaffold^ shows the number of MHC class I genes contained by each scaffold, e.g. 1^3^ indicates that three scaffolds contain one MHC class I gene each.

### 3.2 Recombination and phylogenetic analyses

The elephant showed the highest number of recombination events in MHCI genes, with eight recombinants. Two recombinant sequences each were detected in the pangolin, pig, and macaque; three recombinants each in the horse and tenrec; and three to five in each of the five bat species (Table S2). The recombination detection program RDP4 identified not only the parent sequences of the recombinants but also the recombination breakpoints. Generally, the breakpoints were randomly positioned, such that recombination targeted exon 2 region in some genes, or exon 3, 4, or 5 in others (Table S2). For example, a recombination event occurred in exon 2 in pseudogene Dero_G9_ps and exon 3 in Dero_G25 of the common vampire bat, and in exon 5 of Echtel_G5 of the tenrec. In the great roundleaf bat, recombination in Hiar_G49 involved a stretch from the end region of exon 2 to exon 5 (Table S2).

**Figure 3:**
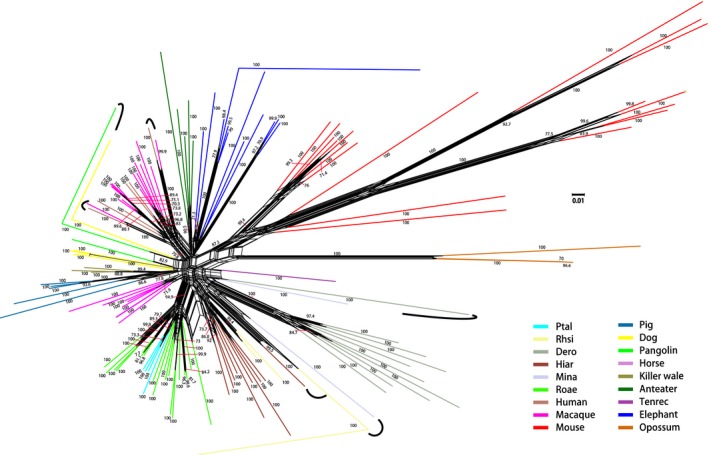
NeighborNet network showing inferred phylogenetic relationships among MHC class I sequences of eutherian mammals, based on a segment of MHCI exons 2–5. Numerals are bootstrap support values for each edge, with only values >70% shown. Edges with high support are thickened. Different colors indicate different species, as defined at lower right. Orthologous sequences are indicated by curved lines. Dero, *D. rotundus* (common vampire bat); Hiar, *H. armiger* (great roundleaf bat); Mina, *M. natalensis* (natal long‐fingered bat); Ptal, *P. alecto* (black flying fox); Rhsi, *R. sinicus* (Chinese horseshoe bat); Roae, *R. aegyptiacus* (Egyptian rousette bat). See Figure S1 for sequence names and accession numbers at the tips of branches

To better understand the evolutionary relationships of eutherian MHCI genes, we also reconstructed a phylogenetic NeighborNet network. Most of the MHCI sequences were clustered by species, indicating paralogous relationships (Figure 3 and Figure S1). In some cases, however, MHCI sequences were grouped by gene rather than by species, indicating orthologous relationships (Figure 3 and Figure S1).

### 3.3 MHCI gene organization in the genome

**Figure 4:**
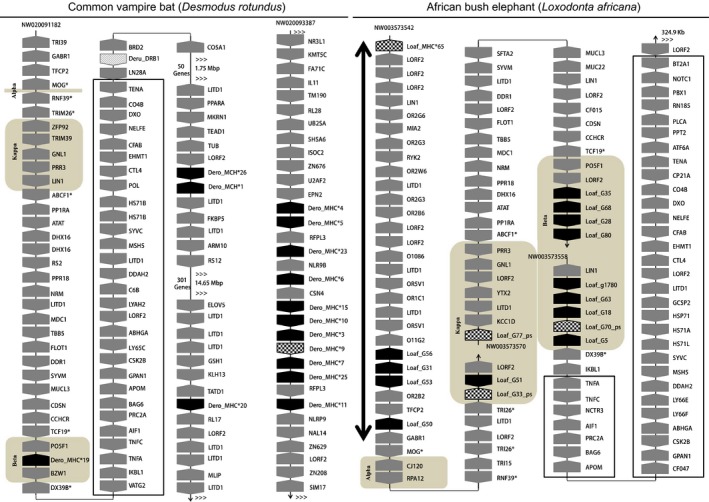
Genomic map of MHC class I (MHCI) genes for common vampire bat (left) and elephant (right). Black boxes indicate putatively functional MHCI genes; gray boxes, non‐MHC I genes; heavily stippled boxes, MHC I pseudogenes. The apex of each box indicates the transcriptional direction. The α, β, and κ blocks are shaded and labeled. An outline rectangle indicates the Class III region. The thick, double‐headed arrow indicates the extended class I region. The lightly stippled box indicates a class II gene. Accession numbers for scaffolds are at the top for each separate fragment. MHCI maps for other species are presented in Figure S2

To understand the origin and evolution of the eutherian MHCI region, we attempted to locate the identified MHCI genes in the genomes by annotating the scaffolds in which MHCI genes were found. Although we could not localize MHCI genes in the genomes of killer whale, pangolin, or anteater due to dispersed distributions on a number of short scaffolds, we were able to determine the organization of MHCI genes and the CMR in the remaining six species. In the elephant, 11 of 31 genes found in 12 scaffolds (Table 1) were located in the CMR—no MHCI genes in the α block, but two (one pseudogene and one PFG) in the β block and nine (one pseudogene and eight PFGs) in the κ block. Interestingly, one pseudogene and four PFGs were found in the extended class I region, while others were either scattered outside the CMR or not localized in the genome (Figure 4 and Figure S2). In the tenrec, the only PFG detected in the CMR was in the κ block, with the remaining genes outside the MHCI region (Figure S2). Similarly, in the four bat species, only the β block contained one or two MHCI genes, with most of the MHCI genes outside the CMR (Figure S2). Uniquely in the common vampire bat, 15 of 18 MHCI genes were identified in two scaffolds (Table 1 and Figure 4), of which one comprised the CMR, while the other lay outside the CMR and contained 11 tightly organized MHCI genes (Figure 4), suggesting a unique MHCI gene duplication in the genome outside the CMR.

In addition, we found no MHC class II region following the class III region in the vampire bat, tenrec, and elephant (Figure 4 and Figure S2), except for one class II gene in the vampire bat, implying that the Class II region in these species is probably unlinked to class III. In the vampire bat, two and one MHCI PFGs in nonclass I regions were detected at positions about 1.75 and 16.40 Mbp, respectively, away from class III (Figure 4). In the tenrec MHC, a 10.16 Mbp insertion was detected in the κ block following the *ERO1A* gene (Figure S2), indicating another split of the CMR in the κ block.

## 4 DISCUSSION

In this study, we attempted to understand the origin and evolution of eutherian MHCI gene organization by analyzing genome assemblies publicly available at NCBI from nine species of eutherian mammals. Of these genome assemblies, eight were obtained by Illumina high‐throughput parallel sequencing, while one (the elephant) was obtained by Sanger sequencing (Table S1). A potential source of error could have been that the sequences analyzed were not true biological sequences, but chimeras or assembly artifacts. However, the accuracy of Sanger sequencing is 99.999%, and that of the Illumina sequencing is 98%–99% after filtering (Liu et al., 2012; Sohn & Nam, 2018). The random sequencing errors can be corrected by the overlapping alignments of numerous short reads (Sohn & Nam, 2018). Despite that short‐read genome assemblies encounter with many challenges, many strategies have been put forward to overcome those challenges (reviewed in Sohn & Nam, 2018). Moreover, genome assembly algorithms might be able to operate at 100% stringency if the sequencing produces error‐free reads at high coverage (Miller, Koren, & Sutton, 2010); indeed, the genome coverages in this study ranged from 60X to 218X for Illimuna sequencing. In addition, although assembly artifacts may be introduced in scaffolding while joining contigs, most scaffolds we analyzed were single‐contig scaffolds. Therefore, although possible chimeras and/or artifacts in these genome assemblies cannot be completely ruled out, we believe that if any occurred, they would not influence the overall results and conclusions of this study. Indeed, our results from two bats are in agreement with previous studies (Ng et al., 2016; Pavlovich et al., 2018). Since the MHCI regions in these species are fragmented to some degree, chromosome‐level genome sequences obtained by a combination of multiple sequencing methods will help confirm our observations.

### 4.1 Evolution of eutherian MHCI repertoires

The number of MHCI genes identified to date in eutherian mammals varies from species to species, ranging from 51 genes in the rhesus macaque (Shiina et al., 2017) to seven in the domestic dog (Yuhki et al., 2007). The number of functional MHCI genes ranges from about four in the domestic dog to 30 in the rhesus macaque (Liu et al., 2017; Shiina et al., 2017; Yuhki et al., 2007). The numbers of both MHCI genes and PFGs identified in most species in our study (Table 1) were well within these ranges, although the killer whale and pangolin were exceptions. The MHCI gene content in the killer whale is similar to that in nonmammalian vertebrates (Belov et al., 2006; Didinger, Eimes, Lillie, & Waldman, 2017; Dohm et al., 2007; Michalova et al., 2000; Ohta et al., 2006), and to that in other cetaceans—Hector's dolphin (Heimeier et al., 2009), Yangtze finless porpoise (Ruan, Wan, Zheng, Zheng, & Wang, 2016), and North Atlantic right whale (Gillett, Murray, & White, 2014). The low number of MHCI genes in cetaceans might be related to the ancestral shift in habitat from land to water, with lower pathogenic selection pressure in aquatic environments compared to terrestrial ones (Slade, 1992). To confirm whether the low number of MHCI genes observed in a small number of species extends to aquatic mammals in general will require detailed studies on additional species. The pangolin, while terrestrial, has fewer MHCI genes than most other terrestrial mammals. Possible explanations are that it has hardened scales covering the body (Spearman, 1967; Wang, Yang, Sherman, & Meyers, 2016), a highly specialized diet (Ashokkumar, Valsarajan, Suresh, Kaimal, & Chandy, 2017; Pietersen, Symes, Woodborne, McKechnie, & Jansen, 2016), and solitary behavior (https://www.pangolins.org), all of which might have reduced their exposure to pathogens.

Bats, on the other hand, are reservoir hosts and vectors for a wide spectrum of viral and fungal pathogens, but show few clinical symptoms (Hayman, 2016); one would expect them to have a large number of MHCI genes. Nonetheless, bats exhibit smaller MHCI repertoires than do the other mammals, such as the rhesus macaque (Shiina et al., 2017), rodents (Kumánovics et al., 2003), and African bush elephant (Table 1). To resist pathogenic disease, humans have accumulated high allelic variation (10,574 alleles for MHCI) among relatively few MHCI loci (eight functional genes), while the rhesus macaque has many regional configurations, with low allelic variation at many different loci (Shiina et al., 2017). Thus, it appears that various mammals have evolved different strategies for defense against pathogens.

**Figure 5:**
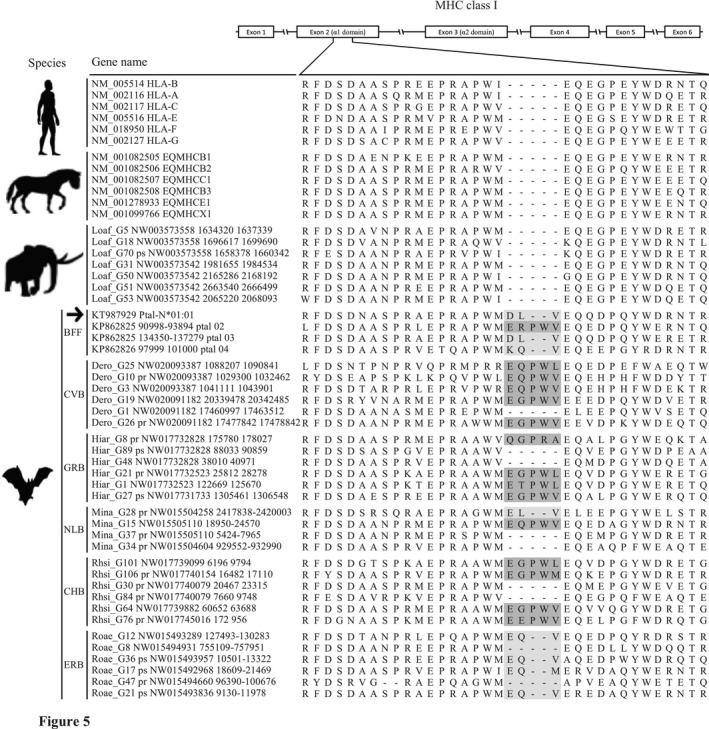
Alignment of deduced amino acid sequences, showing bat‐specific amino acid insertions in the α1 peptide‐binding domain (exon 2) of MHC class I genes. Three‐amino‐acid insertions are shaded in light gray, five‐amino‐acid insertions in dark gray. BFF, black flying fox; CHB, Chinese horseshoe bat; CVB, common vampire bat; ERB, Egyptian rousette bat; GRB, great roundleaf bat; NLB, Natal long‐fingered bat. The antigen presentation function of Ptal‐N*01:01 (arrow) was characterized by Wynne et al. (2016). See Figure S3 for the complete MHCI amino acid sequences

What mechanism, then, could explain the exceptional ability of bats to host pathogens fatal to other mammals? In agreement with the results of Ng et al. (2016), in all four bat species examined in this study representing both suborders of Chiroptera, we found the α1 antigen‐binding domain in MHCI genes to be three or five amino acids (AAs) longer than in the other eutherian mammals (Figure 5 and Figure S3), suggesting that this is a common feature across bats. Pavlovich et al. (2018) showed that MHCI genes with an additional three AAs were transcribed in almost all bat tissues. Most importantly, Wynne et al. (2016) demonstrated that functional MHCI allele Ptal‐N*01:01 in the black flying fox has a three‐AA insertion in the same position, that its product can present virus‐derived peptides on the cell surface, and that the peptides presented show a broader length distribution than those presented by MHCIs in other mammals. This suggests that bat MHCI genes with the five‐AA insertion might also be functional, and might present an even broader length range of peptides, because the peptide repertoire presented by MHC proteins depends largely on structural features of the binding groove in each MHC allelic variant. Thus, we speculate that, in contrast to the rhesus macaque and human, the unique three‐ or five‐AA insertions in bat MHCI genes allow the gene products to present a wider range of peptides which in turn allows bats to better resist pathogens.

Of the six bats studied so far, *D. rudentun* and *M. natalensis* belong to the suborder Yangochiroptera, and *H. armiger*, *Rousettus aegyptiacus*, *P. alecta*, and *R. sinicus* to the other suborder Yinpterochiroptera, indicating that the three‐ or five‐AA insertions may have appeared in the common ancestor of bats approximately 64 million years ago (Miller‐Butterworth et al., 2007; Teeling, 2005). During the coevolution with viral, bacterial, and fungal pathogens (Brook & Dobson, 2015; Mühldorfer, 2013), MHCI genes with three‐ or five‐AA insertions were likely highly selected over shorter ones. Further investigation is needed to understand the evolutionary and functional significance of the five‐AA insertion in bat MHCI genes.

Some MHCI genes in the mouse, human, and macaque—especially nonclassical MHCI genes regarded as monomorphic, with limited tissue distribution—do not necessarily function in presenting antigen to T‐cell receptors, but instead have nonimmune functions (reviewed in Kumánovics et al., 2003; Shiina et al., 2017). The natural roles of the MHCI genes we detected in this study remain to be elucidated, and we cannot rule out the possibilities that some of them have nonimmune functions.

Phylogenetic reconstructions for MHCI genes showed patterns of paralogy, as well as of orthology, the latter defined as sequences descendant from the same ancestral sequence and separated through speciation events; orthologous sequences thus cluster by locus or genes rather than by species (Gu & Nei, 1999; Nei & Rooney, 2005). However, orthologous relationships are typically seen only in relatively closely related taxa, such as among primates or among rodents (Cao et al., 2015; Kumánovics et al., 2003). Our phylogenetic NeighborNet network consistently reflected orthology among bats and among primates, but paralogy among different orders (Figure 3 and Figure S1), apart from the dog DLA‐79 and pangolin Manjave_G15 MHCI pseudogenes. Kumánovics et al. (2003) explained these patterns of paralogy and orthology through a model of MHCI gene expansion and contraction, in which multigene families (and especially MHC genes) appear to have generated or lost some genes during evolution (Gu & Nei, 1999; Nei & Rooney, 2005). This is congruent with the general trend that as the number of gene duplications increases, so does the number of pseudogenes (Nei, Gu, & Sitnikova, 1997), and we found one to 12 pseudogenes in all but one species we studied.

Nei and Rooney (2005) concluded that the effect of recombination on MHC variation is quite minor. However, recombination has had a complex effect on the molecular evolution of coding sequences (Rousselle et al., 2019) and is markedly evident in the generation of MHC variation (Abduriyim et al., 2017, 2019; Schaschl et al., 2005). Indeed, we detected signatures of recombination events across all eutherian taxa we examined (Table S2). It appears that recombination contributes to MHCI variation and/or pseudogenization in eutherian mammals to some extent, as some of the recombinants we identified were pseudogenes (Table S2), highlighting the complex nature of MHC gene evolution.

### 4.2 MHCI gene organization and origin of MHCI region in eutherians

It appears that MHCI genes show more complex content and genomic organization in eutherian mammals than in lower vertebrates (Kulski et al., 2002). Studies on MHCI organization in eutherians have focused mainly on model species or domesticated animals (Beck et al., 2005; Chardon et al., 1999; Gustafson et al., 2003; Kulski et al., 2002; Liu et al., 2006; Renard et al., 2006; Yuhki et al., 2007), but rarely on species in the wild (Ng et al., 2016). In this study, we investigated MHCI gene content and organization in the genomes of wild species. Consistent with the findings of Ng et al. (2016) and Pavlovich et al. (2018), we found for all four bat species, that within the canonical MHCI region (CMR), only the β block contained one to several MHCI genes, whereas most MHCI genes were apparently outside the CMR (Figure S2). Intriguingly, uniquely in the common vampire bat, 11 of 18 genes were detected flanking one another on a scaffold not in the CMR (Figure 4), suggesting that a regional duplication has occurred. In the vampire bat, representing the first observation of MHCI gene duplication outside of class I region, it might be related to this species' sanguivorous feeding (Greenhall, Joermann, Schmidt, & Seidel, 1983), which has resulted in direct exposure to pathogens in the blood they feed on. Unexpectedly, we also found MHCI genes outside the CMR in the tenrec and elephant (Figure S2); two genes have also been reported outside the CMR in the dog genome (Yuhki et al., 2007). These observations indicate that, while occurrences of MHCI genes outside the CMR are a common feature in the genomes of some eutherian groups, they occur haphazardly and species‐specifically in others (Figure S2). Interestingly, in the elephant we found MHCI genes in an extended MHCI region (Figure 4), a pattern previously reported only in rodents so far (Lambracht, Prokop, Hedrich, Lindahl, & Woniget, 1995; Yoshino et al., 1998). The similarity of MHCI genes in this extended region in two distantly related taxa is striking, and raises the question whether similar long‐term pathogenic burdens have resulted in the similar gene distributions. However, the mouse MHCI molecule H2‐M3, whose coding gene is in the extended MHCI region, presents peptides that are inherently different from peptides presented by classical MHCI; it may present peptides in substantially larger quantities or higher concentrations due to lack of competition from self‐peptides (Colmone & Wang, 2006). Xu, Chun, Choi, Wang, and Wang (2006) have shown a unique role for H2‐M3‐restricted T cells in host defense against bacterial infection as well. It thus remains to be seen whether this similarity between the mouse and elephant is evolutionarily and functionally significant, and whether the functions of the elephant genes resemble those of mouse H2‐M3. Thus, our findings emphasize that MHCI gene organization in eutherian mammals is more complex than previously thought.

**Figure 6:**
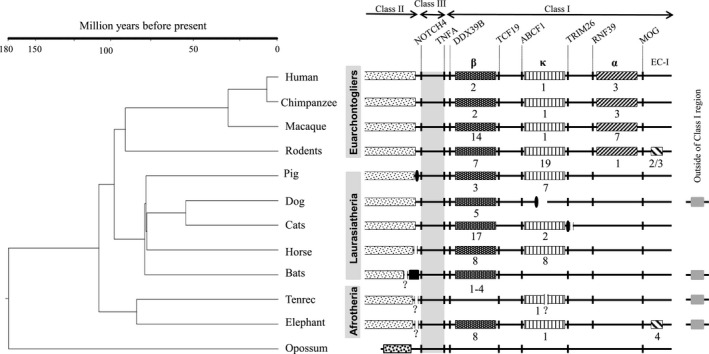
Diagram showing MHC organization in eutherian mammals. Vertical bars in each row indicate non‐MHC genes that are labeled near the top of the figure. Differently shaded boxes indicate the β, κ, and α duplication blocks, and the extended class I region (EC‐1), all labeled at the top of each column; the numbers below the boxes indicate the number of class I genes in that duplication block; finely stippled boxes, class II region; vertical black ellipses, centromeres; black box, bat‐specific MHCI gene region flanked by class III region; question marks, possible splits in the MHC region at the positions indicated; heavily stippled box in opossum, mixed class I and II region

Comparison of the distribution of MHCI genes within the CMR among eutherian mammals (species in our study with those in previous studies) revealed that MHCI genes occupy relatively fixed regions, that is, the α, κ, and β blocks (Figure 6). This finding is in accordance with the framework hypothesis that permissive areas interspersed among highly conserved non‐MHC genes can be filled by the expansion of MHCI genes (Amadou, 1999). Researchers studying the evolution of the CMR and the origin of the three MHCI duplication blocks in eutherian mammals, initially postulated based on observations in the human, mouse, rat, and pig, that all three duplication blocks were present in the common ancestor of eutherian mammals, and were subsequently lost in a taxon‐specific fashion (Kumánovics et al., 2003). In contrast, by studying bats, Ng et al. (2016) proposed that the three blocks arose in a stepwise manner, with the β block ancestral to the κ and then the α blocks. From our results (Figure 6), we hypothesize that MHCI and II genes were separated after the divergence between eutherian and marsupial mammals more than 180 million years ago (Woodburne, Rich, & Springer, 2003); MHCI genes were translocated from the ancestral mammalian MHC set (Belov et al., 2006) into the κ and β blocks of the CMR in eutherian mammals, but remain unchanged in marsupials (Belov et al., 2006). Subsequently, eutherian MHCI genes duplicated within the CMR (bats and the tenrec appear to be exceptions) and were further translocated across the genome in a lineage‐ or species‐specific manner, such as in the elephant, tenrec, dog, and bats. The MHCI genes in α block were established more than 100 million years ago (Misawa & Janke, 2003; Murphy, Pevzner, & O'Brien, 2004) in the common ancestor of Euarchontoglires (Figure 6) and have duplicated in the primate lineage.

It is noteworthy that we found possible breaks/insertions in the tenrec, elephant, and vampire bat MHC (Figure 4 and Figure S2). Similarly, centromere invasion has occurred at the border of the class II and III regions in the pig (Renard et al., 2006). Both the cat and dog exhibit exactly the same chromosome split in MHC structure: the cat class II, III, and I regions are organized in the pericentromeric region of the long arm of chromosome B2, whereas the remaining MHC is located in the subtelomeric region of the short arm of the same chromosome; the dog class II, III, and I regions are found in the pericentromeric region of chromosome 12, while the remaining region occupies the subtelomeric region of chromosome 35 (Yuhki et al., 2007). Thus, the high level of integrity of the MHC seen in the human and macaque might be among a few exceptional cases in the eutherian MHC.

## 5 CONCLUSIONS

Our findings highlight that the MHCI gene distribution in eutherian mammals is more complex than previously thought. Based on observations of the MHCI genomic organization in multiple eutherian mammals, we hypothesize that both the κ and β duplication blocks were present in the common ancestor of eutherians, arising through translocation of MHCI genes from the marsupial‐like class II/ I common region. The α duplication block, on the other hand, was found only in Euarchontoglires, suggesting that this region originated more than 100 million years ago in the common ancestor of Euarchontoglires (Figure 6). The existence of MHCI genes in an extended MHCI region in the elephant and rodents is striking, and similar long‐term pathogenic burdens in these animals may explain this phenomenon. Intriguingly, we also found three‐ or five‐amino‐acid insertions in the α1 domain of MHCI to be specific to bats. Instead of having a limited number of genes with large allelic variation as in human, or vice versa as in macaque (Shiina et al., 2017), bat MHCIs may have a wider peptide‐binding groove that allows them to better resist pathogens; it has been shown that a bat MHCI gene with a three‐amino‐acid insertion presents a larger peptide repertoire than the genes in other mammals (Wynne et al., 2016). The occurrences of the bat‐specific insertions across the bat species studied, representing both suborders Yinpterochiroptera and Yangochiroptera, indicate that these insertions originated in the common ancestor of bats approximately 64 million years ago (Miller‐Butterworth et al., 2007). Moreover, it seems that aquatic mammals have a small number of MHCl genes possibly due to reduced pathogenic burdens in aquatic environments. The MHCI repertoire in some eutherians is likely related to feeding and behavioral ecology as well. Altogether, our results indicate that an adaptation of eutherians to diverse environments and ecological niches with different pathogenic burdens and/or profiles might have driven the evolution of eutherian MHCI repertoire and distribution in a taxon‐specific manner.

## CONFLICT OF INTEREST

The authors declare that they have no conflict of interest.

## AUTHOR CONTRIBUTIONS

S.A. and H.Z. conceived and designed the study; S.A. analyzed data with contributions from D‐H.Z and H.Z.; S.A. and H.Z. wrote the paper; all authors read and approved the manuscript.

## Data Availability

Nucleotide sequences of MHC class I genes derived from genome sequences of nine eutherian mammals in this study were provided in the Data S1.
