# Supplementary material for: Origin and evolution of the major histocompatibility complex class I region in eutherian mammals
Source: Ecol Evol. 2019 Jun 14;9(13):7861–74. doi: 10.1002/ece3.5373 (PMC6636196; doi:10.1002/ece3.5373)
Supplement: Supplementary file 1 [file ECE3-9-7861-s001.pdf]

Supporting Information for

Origin and evolution of the MHC class I region in eutherian mammals

Shamshidin Abduriyim, Da-Hu Zou, and Huabin Zhao*

Department of Ecology, Hubei Key Laboratory of Cell Homeostasis, College of Life Science, Wuhan University, Wuhan 430072, China

***Corresponding author:** Huabin Zhao (Email: huabinzhao@whu.edu.cn)

This supporting file includes:

Tables S1-S2

Figures S1-S3

Supplementary Table S1. Genome assembly information for nine species investigated in this study.

Suborder/Order	Family	Species	Assembly	Contig N50 (bp)	Sequencing platform	Coverage	Genebank accession
Yangochiroptera	Phyllostomidae	*Desmodus rotundus*	ASM294091v2	80250	Illumina Hiseq	94x	PEHR00000000
	Miniopteridae	*Miniopterus natalensis*	Mnat.v1	29777	Illumina HiSeq	77.0x	LDJU00000000
Yinpterochiroptera	Rhinolophoidae	*Hipposideros armiger*	ASM189008v1	39863	Illumina HiSeq	218.6x	JXIK00000000
		Rhinolophus sinicus	ASM188883v1	37803	Illumina HiSeq	146.44x	LVEH00000000
Artiodactyla	Delphinidae	*Orcinus orca*	Oorc_1.1	70300	Illumina HiSeq	200.0x	ANOL00000000
Pholidota	Manidae	*Manis javanica*	ManJan1.0	38922	Illumina HiSeq	60.0x	JSZB00000000
Pilosa	Myrmecophagidae	*Myrmecophaga tridactyla*	MyrTri_v1_BIUU	36434	Illumina HiSeq	28.9x	PVIY000000000
Afrosoricida	Tenrecidae	*Echinops telfairi*	EchTel2.0	20428	Illumina HiSeq	78x	AAIY00000000
Proboscidea	Elephantidae	*Loxodonta africana*	Loxafr3.0	69023	Sanger; ABI.	7x	AAGU00000000

Supplementary Table S2. Recombinant sequences and their parent sequences detected in this study.

**Species**	**Breakpoint Positions**		**Recombinant^**	**Minor Parent Sequence**	**Major Parent Sequence**	**Detection Methods (*P* values)**							
	**Begin**	**End**				**RDP**	**GENECONV**	**Bootscan**	**Maxchi**	**Chimaera**	**SiSscan**	**LARD**	**3Seq**
Dero	333	498	Dero_G25	Unknown (Dero_G1)	Dero_G6	0.003	0.026	NS	0.000	0.000	0.003	NS	0.000
	551*	732	Dero_G19	Dero_G25	Dero_G1	0.000	0.013	0.014	0.000	0.000	0.000	0.000	0.000
	1*	76	Dero_G9_ps	Unknown (Dero_G20)	Dero_G5	0.000	NS	0.000	0.000	0.000	0.000	NS	0.000
	553	825	Dero_G20	Dero_G7	Dero_G1	0.000	0.000	0.000	0.000	0.000	0.000	0.000	0.000
Roae	1*	314	Roae_G40_ps	Unknown (Roae_G13)	Roae_G26	0.019	0.050	NS	0.000	NS	0.000	0.000	0.014
	9*	301	Roae_G20	Roae_G6_ps	Roae_G27_ps	0.030	NS	0.015	0.000	0.004	0.000	0.000	NS
	238	430	Roae_G18#	Roae_G36_ps	Roae_G27_ps	NS	NS	NS	0.000	0.017	0.000	0.000	NS
	722	881	Roae_G36#	Roae_G1	Roae_G39	NS	0.000	0.000	0.003	0.017	0.000	NS	0.005
Ptal	9*	310	Ptal_02#	Ptal_05	Unknown (Ptal_04)	0.000	0.006	0.001	0.000	0.000	0.000	0.000	0.000
	450	934*	Ptal_0101#	Ptal_03	KP862828_ptal_0102	0.049	NS	0.032	0.000	0.000	0.000	0.000	NS
	461*	564	Ptal_02#	Ptal_0102	Unknown	0.0	NS	0.017	0.041	0.013	0.002	0.00	0.0

					(Ptal_04)	05						0	02
Mina	272	379	Mina_G37#	Mina_G34	Mina_G15	0.000	0.000	0.000	0.000	0.000	0.000	0.000	0.000
	388	545	Mina_G28#	Unknown (Mina_G15)	Mina_G34	0.006	0.003	NS	0.000	0.001	0.000	NS	0.003
	38*	207	Mina_G28#	Mina_G15	Mina_G34	NS	NS	0.005	0.001	0.001	0.000	0.012	0.000
Hiar	270	498	Hiar_G21#	Hiar_G34	Hiar_G1	0.001	0.005	0.000	0.000	0.029	0.000	0.000	0.000
	243	820*	Hiar_G49_pr	Unknown (Hiar_G15_ps)	Hiar_G91	NS	NS	NS	0.000	0.000	0.006	0.000	NS
	68*	211	Hiar_G8_pr	Hiar_G28_pr	Hiar_G91	0.001	NS	0.000	0.000	0.000	0.000	NS	0.000
	1*	234	Hiar_G91#	Unknown (Hiar_G1)	Hiar_G15_ps	NS	NS	NS	0.002	0.001	NS	0.014	0.034
	339	555	Hiar_G27_ps#	Unknown (Hiar_G28)	Hiar_G15_ps	NS	0.001	NS	0.049	NS	0.000	0.002	NS
Pangolin	1*	282	Manjav_G14#	Unknown (Manjav_G15_ps)	Manjav_G2	0.000	0.003	0.012	0.008	0.000	0.000	0.000	0.000
	462	498	Manjav_G5#	Manjav_G15_ps	Manjav_G2	0.002	NS	0.010	0.032	0.006	0.000	0.000	0.020
Dog	452	570	DLA-64#	Unknown (DLA-79)	DLA-12	0.001	0.000	0.000	0.000	0.000	0.000	0.000	0.000
Horse	96*	246	Eqca-N	Eqca-16	EQMHCB1	0.000	0.030	0.001	0.000	0.000	0.005	0.002	NS
	30*	214	EQMHCB	Unknown	EQMHCB1	NS	0.020	NS	0.000	0.012	0.011	0.00	0.0

			2	(EQMHCX1)								1	03
	13*	214	Eqca-16#	EQMHCX1	Eqca-1	NS	0.036	NS	0.024	0.048	NS	NS	0.031
Pig	508	710*	SLA-3#	Unknown (SLA-8)	SLA-7	0.004	NS	0.000	0.006	0.003	0.000	0.000	0.025
	316	473	SLA-6#	SLA-7	Unknown (SLA-8)	0.024	NS	0.012	0.044	0.024	0.017	0.000	NS
Macaque	546	892*	Mamu-B7	Mamu-B13	Mamu-AG1	NS	NS	0.029	0.000	0.000	0.000	0.044	NS
	32*	84	Mamu-B12#	Unknown (Mamu-E)	Mamu-B15	0.003	0.010	0.002	NS	0.025	0.000	NS	0.036
Elephant	9*	337	Loaf_G82_ps	Loaf_G50	Unknown (Loaf_G53)	0.035	0.044	0.026	0.000	0.000	0.001	0.000	0.000
	141*	317	Loaf_G53#	Unknown (Loaf_G51)	Loaf_G50	0.006	NS	0.012	0.040	0.016	NS	NS	0.003
	26*	313	Loaf_G70_ps#	Loaf_G5	Loaf_G11	0.003	0.000	0.000	0.000	0.019	0.002	NS	0.000
	469	932*	Loaf_G5	Loaf_G18	Loaf_G51	0.027	NS	NS	0.024	0.000	0.000	NS	NS
	133	245	Loaf_G39_ps	Loaf_G33_ps	Loaf_G11	0.005	NS	0.004	0.004	0.026	NS	NS	0.006
	1*	360	Loaf_G33_ps	Unknown (Loaf_G31)	Loaf_G51	NS	0.028	NS	NS	0.022	0.000	0.026	0.000
	17*	198	Loaf_G25_ps#	Loaf_G50	Unknown (Loaf_G11)	0.007	NS	NS	0.009	0.020	NS	NS	0.048
	9*	309*	Loaf_G11#	Unknown	Loaf_G50	0.0	NS	0.022	0.002	0.000	0.000	0.00	NS

				(Loaf_G51)		45						9	
Tenrec	28*	160	Echtel_G59#	Echtel_G38	Echtel_G5	0.000	0.000	0.000	0.006	0.000	NS	NS	0.000
	802	944*	Echtel_G5#	Echtel_G38	Echtel_G59	0.036	NS	NS	0.000	0.002	0.000	NS	0.000
	204	493	Echtel_G36#	Unknown (Echtel_G59)	Echtel_G38	0.040	NS	NS	0.002	0.001	0.000	NS	0.006

Note- Minor Parent: Parent contributing the smaller fraction of sequence. Major Parent: Parent contributing the largest fraction of sequence. Unknown: Only one parent and a recombinant need be in the alignment for recombination event to be detectable. The sequence listed as unknown was used to infer the existance of a missing parental sequence. NS: Not significant. * The breakpoint position was most likely overprinted by a subsequent recombination event. # It is possible that this apparent recombination signal could have been caused by an evolutionary process other than recombination. ^ One of the identified parents might be the recombinant in some events.

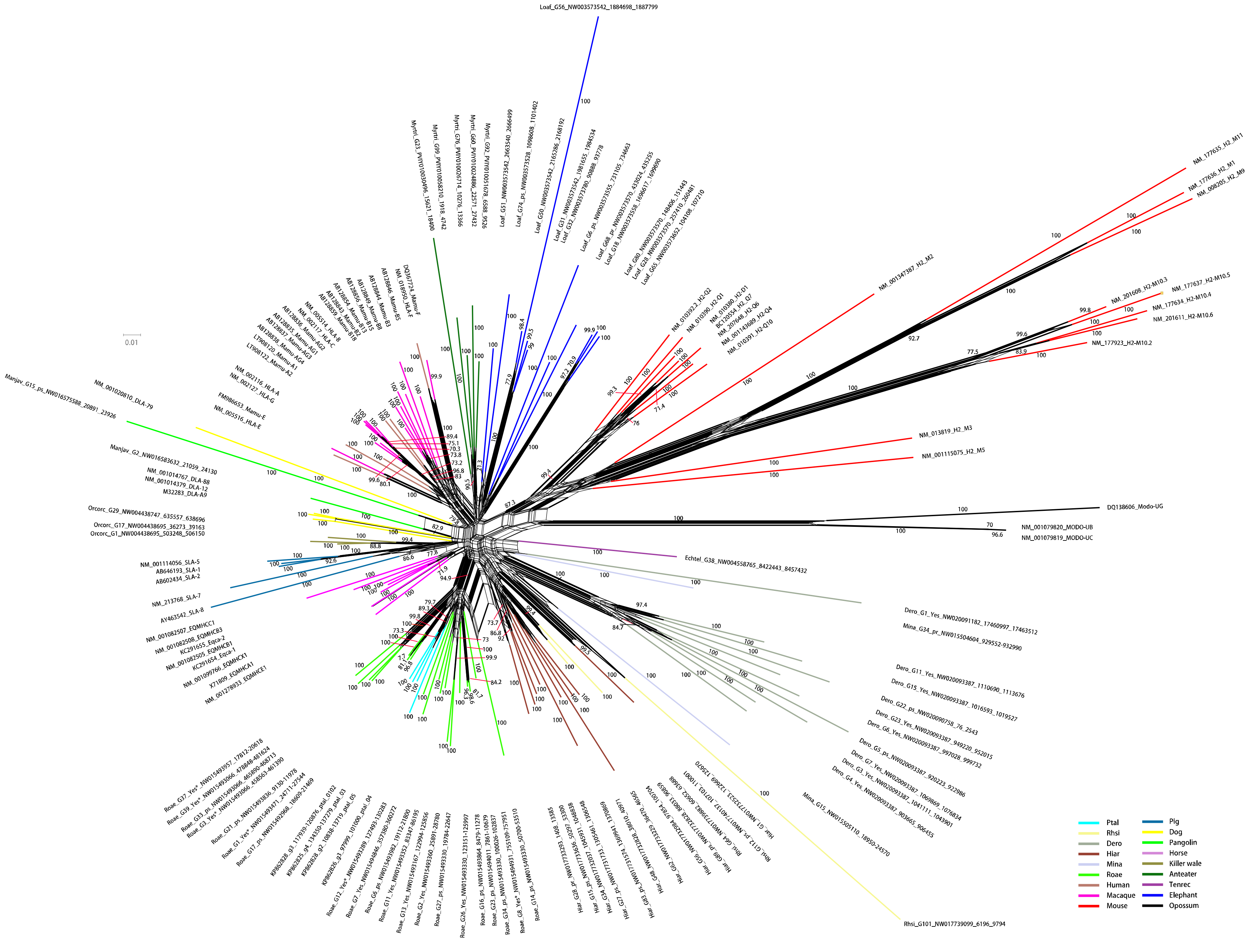

Supplementary Figure 1. NeighborNet network showing inferred phylogenetic relationships among MHC class I sequences of eutherian mammals, based on a segment from MHCI exons 2 to 5. Numerals are bootstrap support values for each edge, with only values >70% shown. Edges with high support are thickened. Different colors indicate different species, as defined in the key at lower right. Abbreviations: Ptal, P. alecto (black flying fox); Rhsi, R. sinicus (Chinese horseshoe bat); Dero, D. rotundus (common vampire bat); Hiar, H. armiger (great roundleaf bat); Mina, M. natalensis (natal long-fingered bat); Roae, R. aegyptiacus (Egyptian rousette bat). The sequence names and accession numbers at the tips of branches: GeneBank accession numbers followed by gene names were given for the sequences obtained from NCBI; gene name before the scaffold accession number, start and end positions was shown for sequences derived from genomes in this study.

Supplementary Figure 2

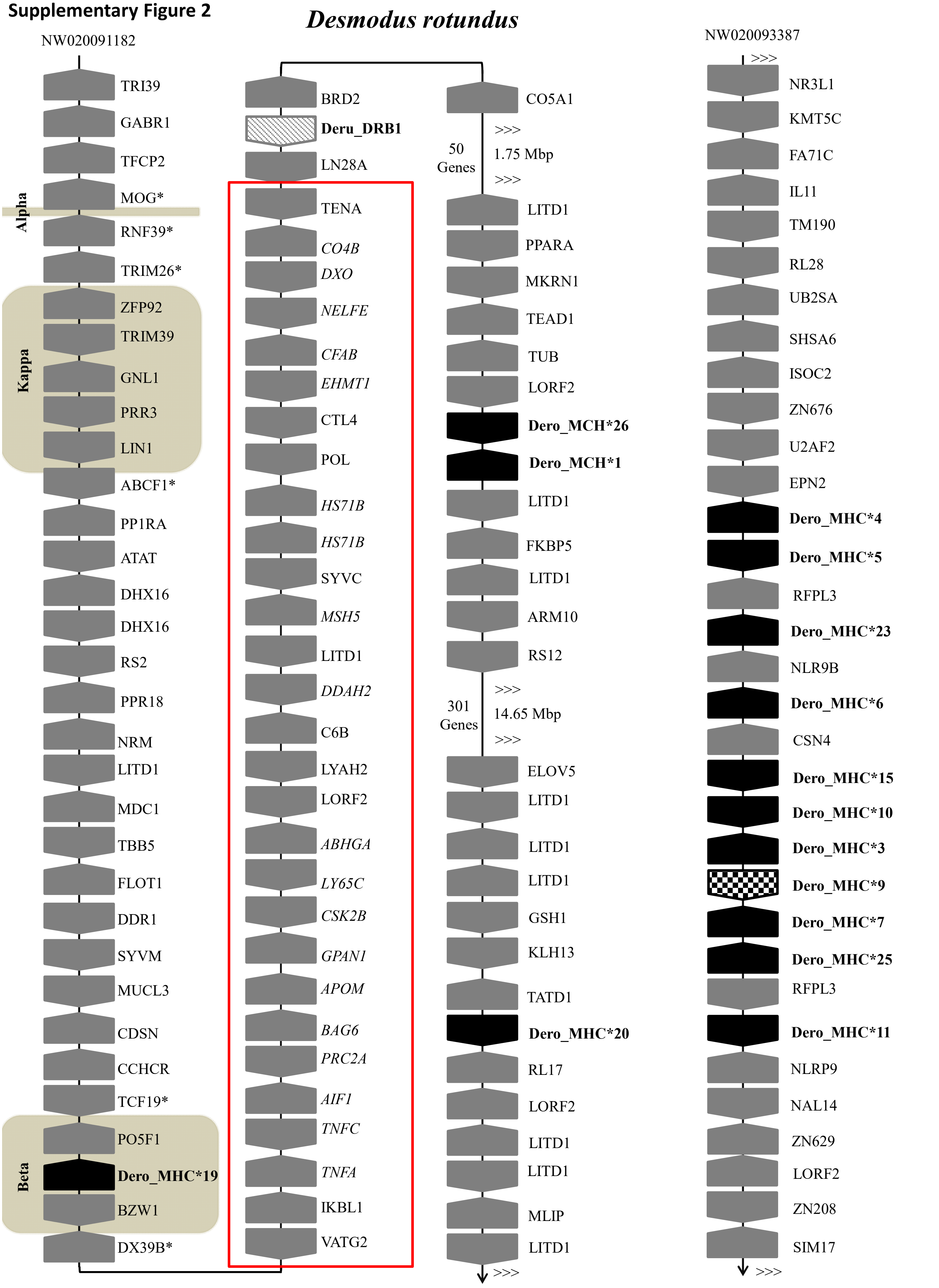

Supplementary Figure 2 continued

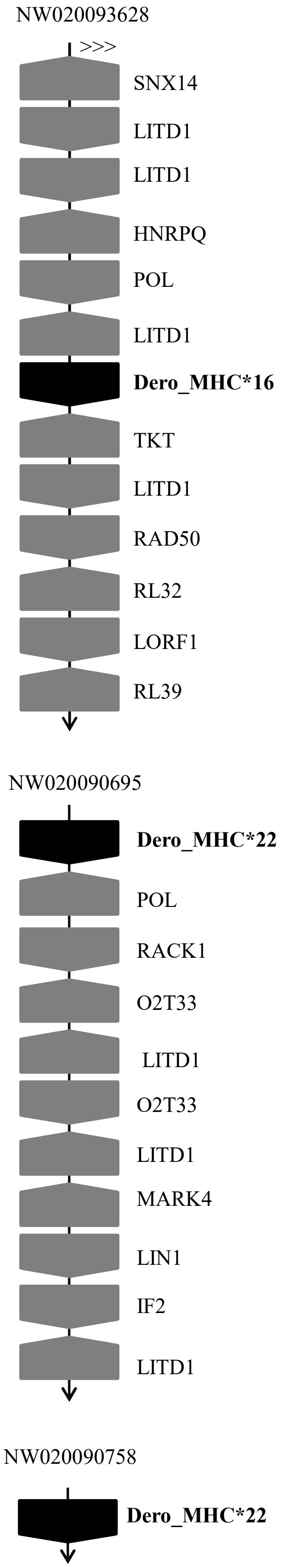

Supplementary Figure 2. Genomic map of MHC class I (MHCI) genes for nine species of eutherian mammals. Black boxes indicate putatively functional MHCI genes; gray boxes, non-MHC I genes; heavily stippled boxes, MHC I pseudogenes. The apex of each box indicates the transcriptional direction. The α, β, and κ blocks are shaded and labeled. An outline red rectangle indicates the Class III region. The thick, double-headed arrow indicates the extended class I region. The lightly stippled box indicates a class II gene. Accession numbers for scaffolds are at the top for each separate fragment.

Supplementary Figure 2 continued

Hipposideros armiger

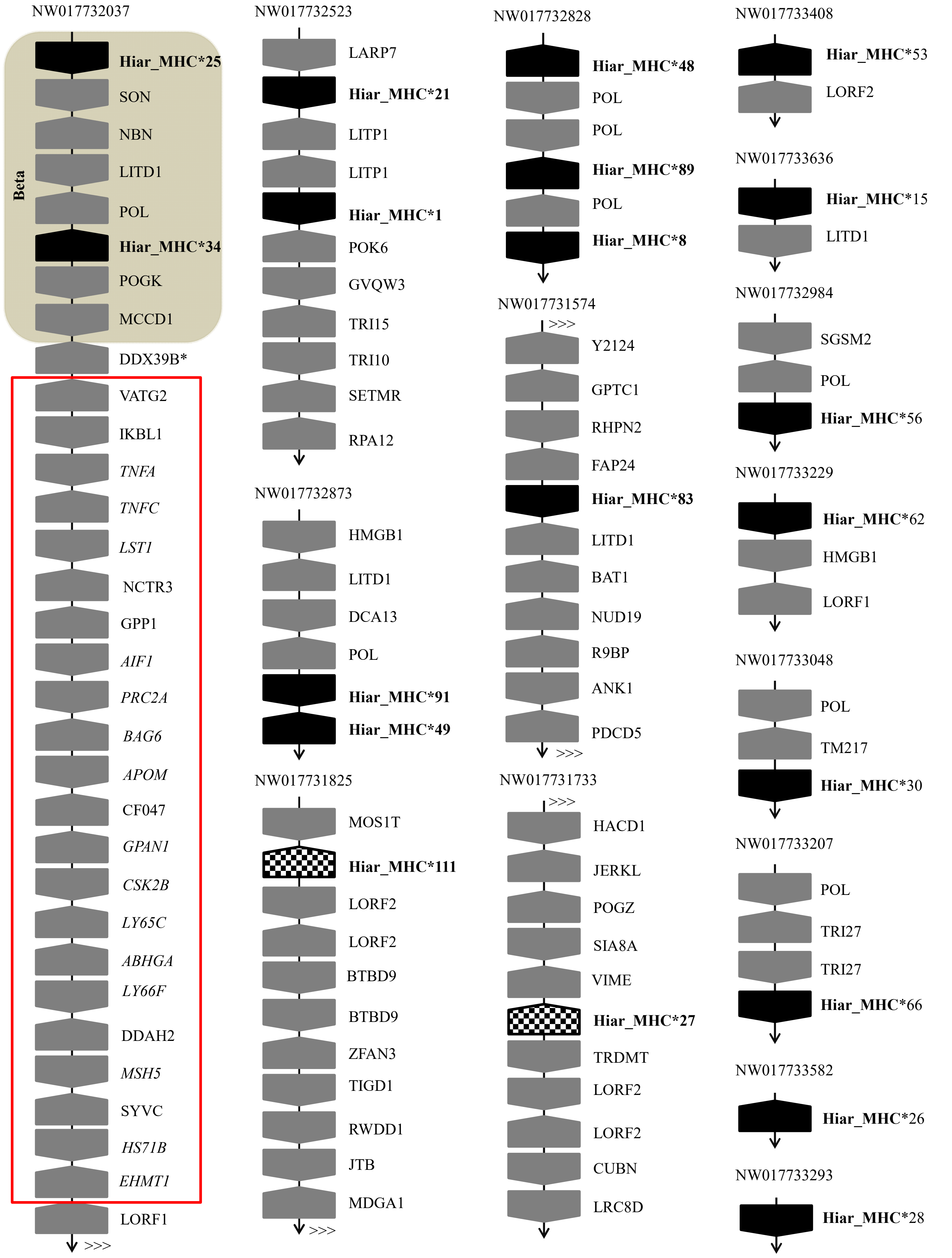

Supplementary Figure 2 continued

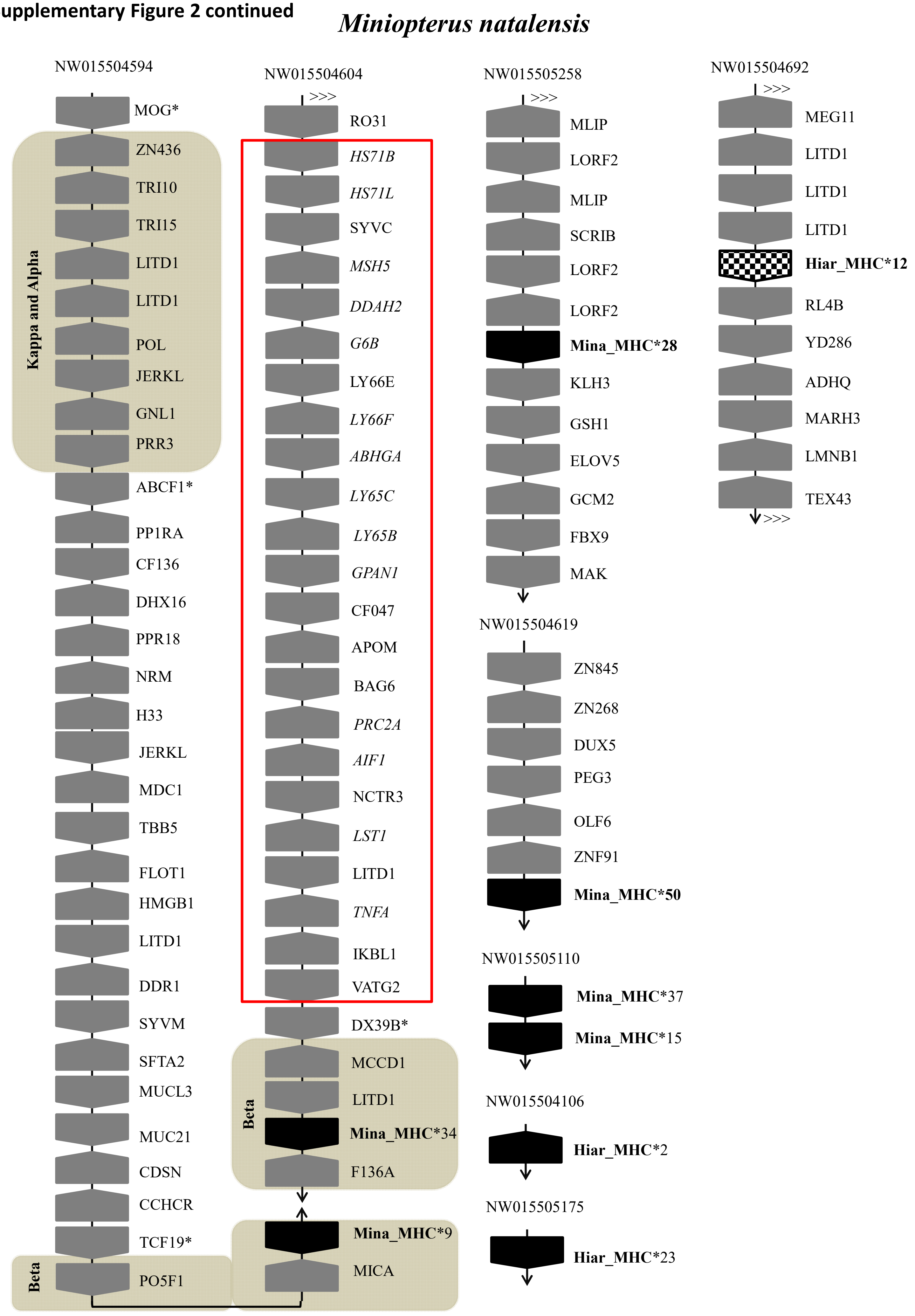

Supplementary Figure 2 continued

Rhinolophus sinicus

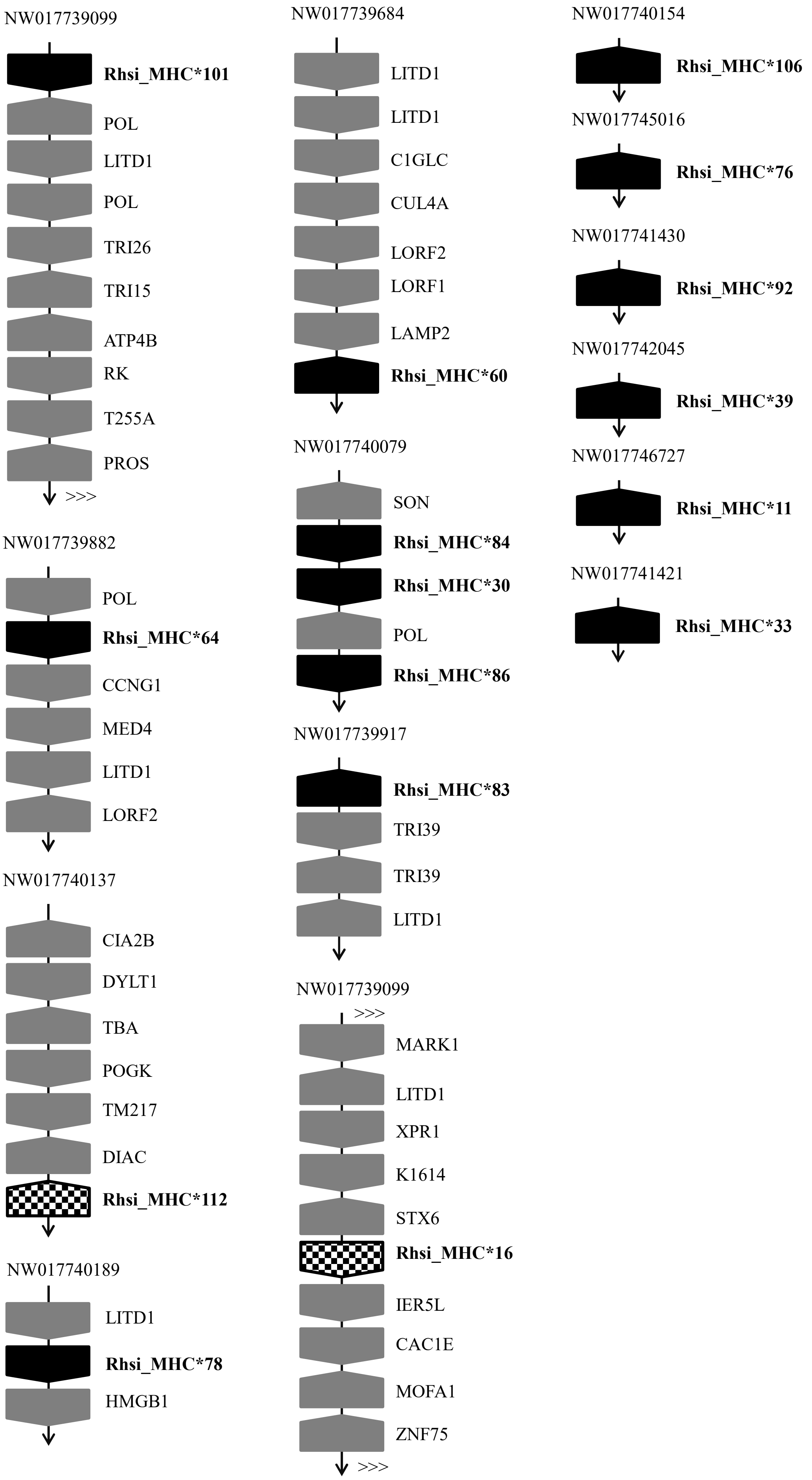

Supplementary Figure 2 continued

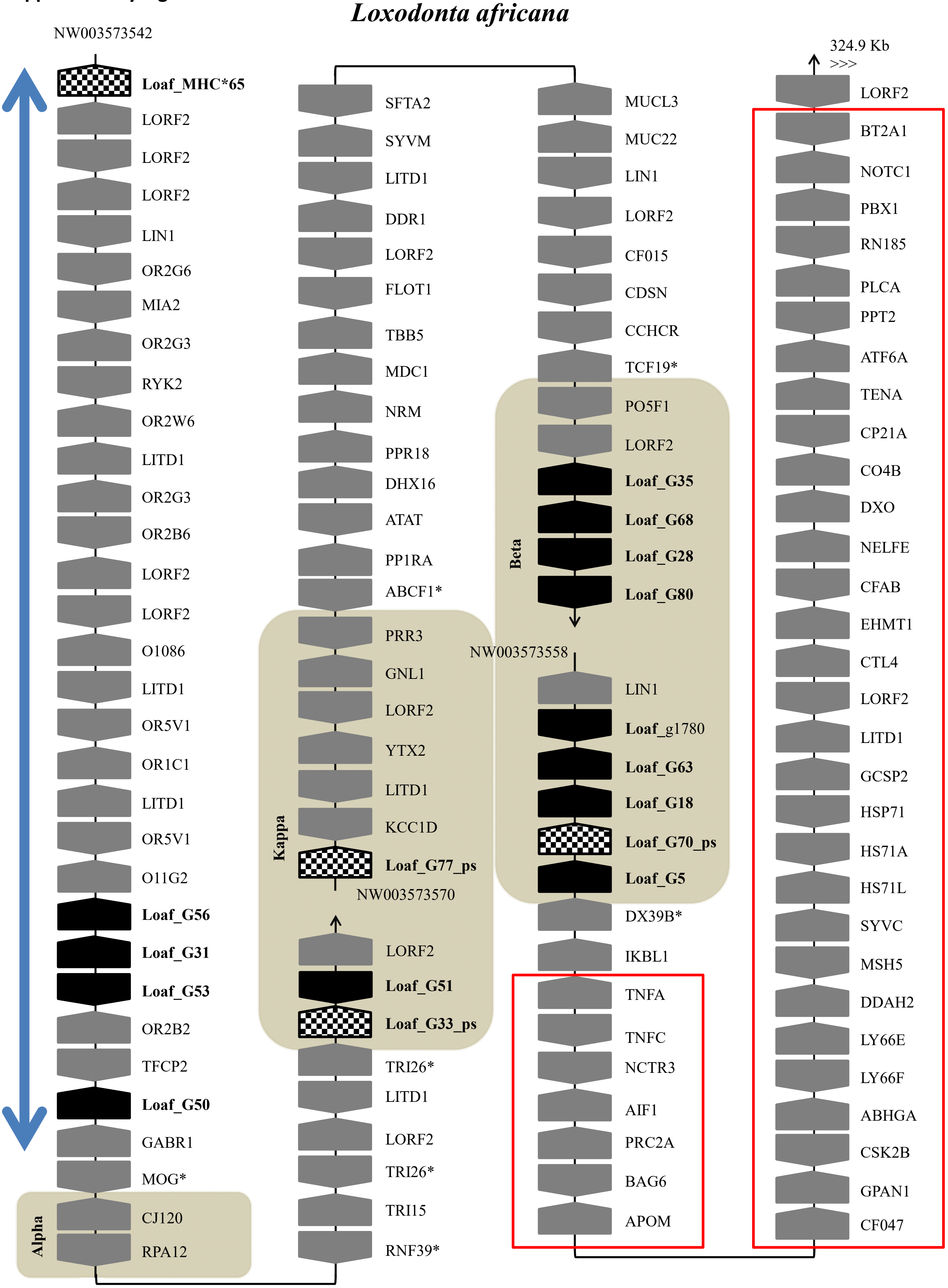

Supplementary Figure 2 continued

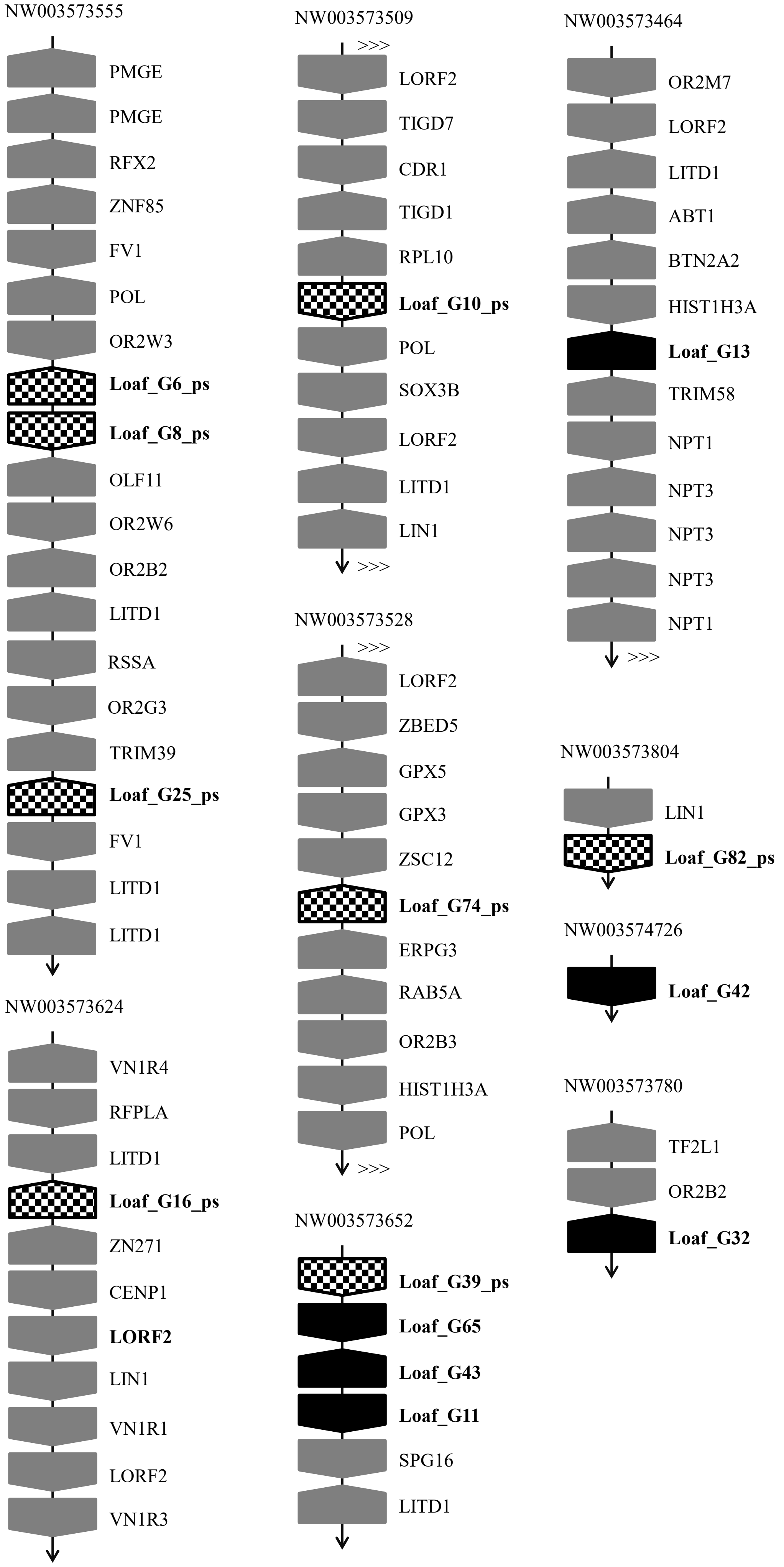

Supplementary Figure 2 continued

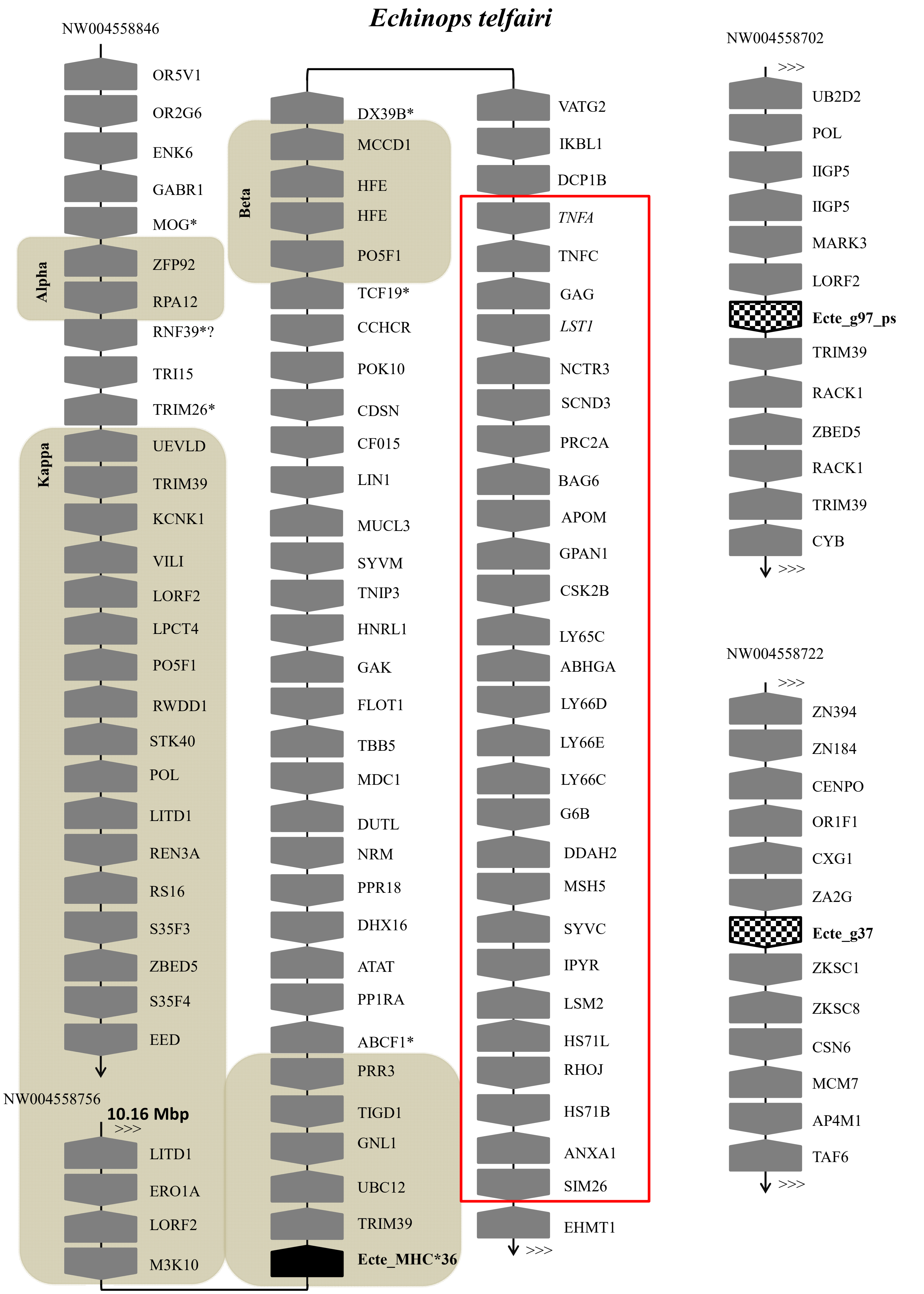

Supplementary Figure 2 continued

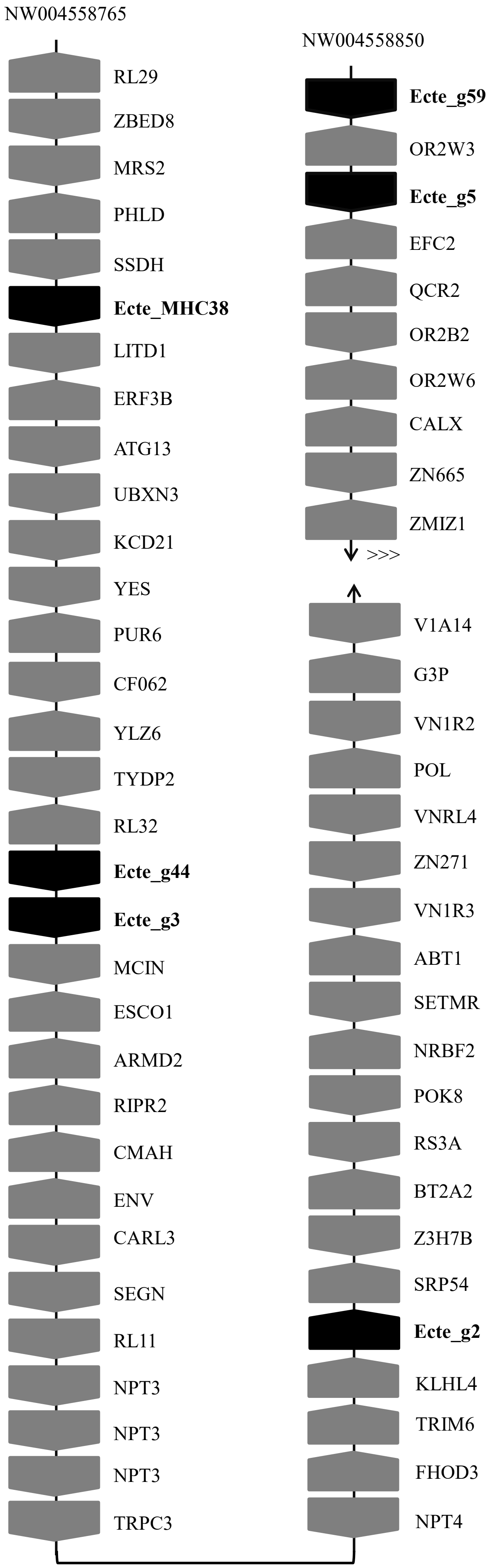

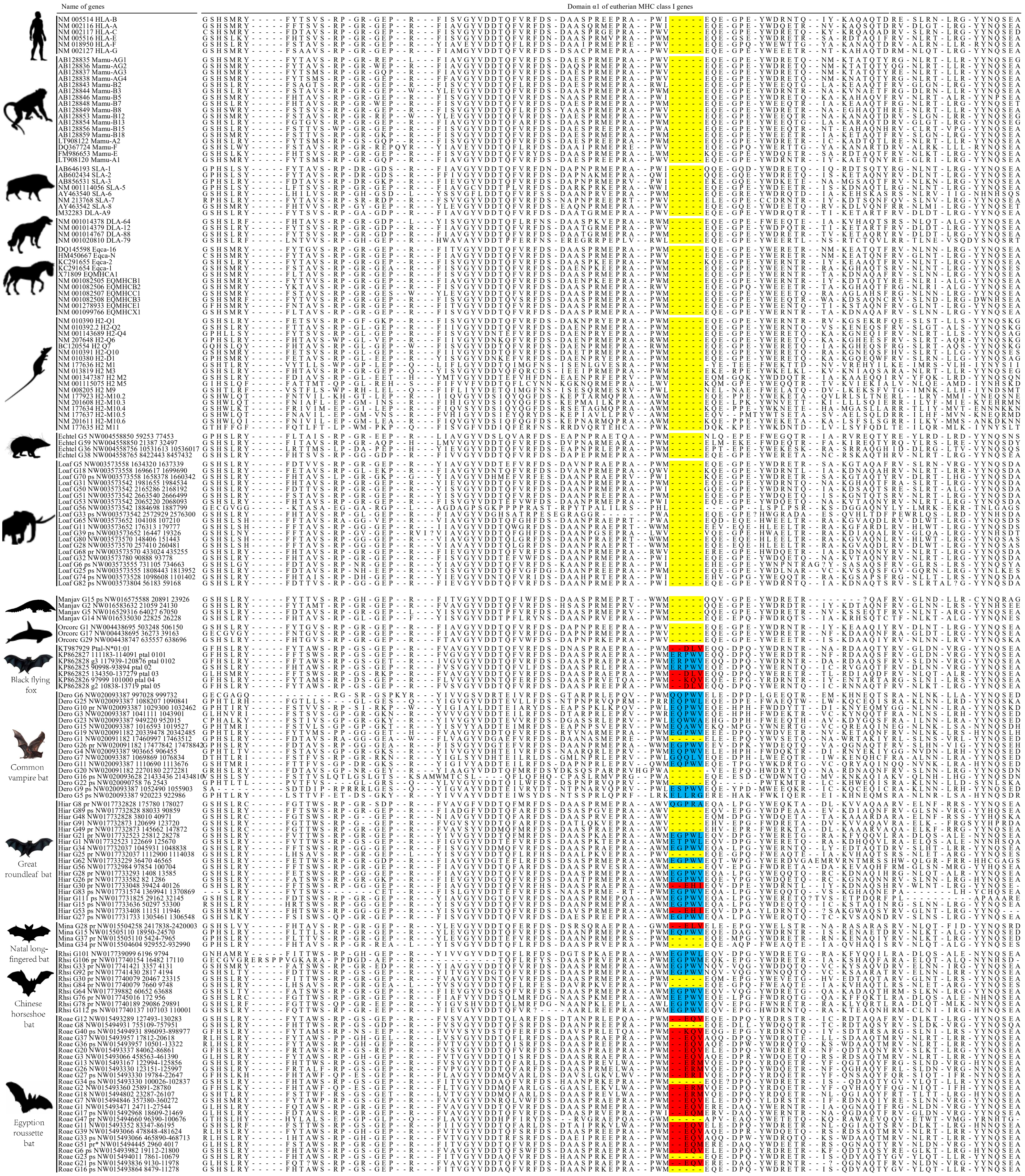

Supplementary Figure 3. Alignment of deduced amino acid sequences, showing bat-specific amino acid insertions in the α1 peptide binding domain (exon 2) of MHC class I genes. Three-AA insertions are shaded in red, five-AA insertions in blue.
