# Supplementary material for: Origin and evolution of the major histocompatibility complex class I region in eutherian mammals
Source: Ecol Evol. 2019 Jun 14;9(13):7861–74. doi: 10.1002/ece3.5373 (PMC6636196; doi:10.1002/ece3.5373)
Supplement: Supplementary file 2 [file ECE3-9-7861-s002.docx]

**Supplementary data set S1.** Nucleotide sequences of MHC class I genes derived from genome sequences of nine eutherian mammals in this study.

The sequence names are shown as follows, taking “**Dero_G6_NW020093387_997028_ 999732**” as one example below.


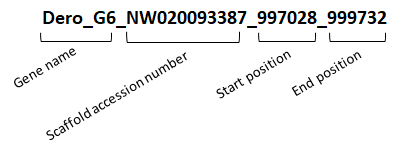


1. **Common vampire bat *Desmodus rotundus***

>Dero_G6_NW020093387_997028_999732

CCAGAAGCTCAGGGGCCCCAAACCTTGCTCCTGCTGCTCTCCGGGGCCCTGGCCCTGAGAAGGACCGGGGCTGGTGAGTGCGGGGCAGGACAGAGGGGATCCCGCGGGAGCCCAAAATACCGGTACATCGTTGTCGGCTCCGTGGACCGCACGGAGATCGTGCGGTTCGACAGCGGCACAGCGAGGCCGAGGCTGGAGCCGCAGGTACCGTGGATGCAGCAGCCGTGGGTGGAGCTGGAGGATCCAGATTTGGGGGAGGAACAGACACGGGAAATGAAGCACAATGAGCAGACTTCCCGAGCGAACCTGAACAAGCTTCTCGCCTACTACAACCAGAGCGAGGACGGATCTCACACCTTCCAGGATAGGACCCGCTGCGTCCTGGGCTCAGACGGGCGCATCCTCCGCGGGTACAGTCGGTTCGCCTACGACGGCACCGACTACCTCGCCCTGAACAAGGACCTGCGCTCCTGGACCACGGTGTACACGGCGTCTGGGATCAGCTGGCACCCCTTGGTGCGGGTCCCTGAAGCGAACGTCAGGAGATTCTTCCTGGAGGACACGTGCGTGCACCGGCTCCGCCTGCTCCTGGAGAAGGGGAATGAGATGCTCCTCCGAGCAGACCCTCCAAATACACACGTGACCCACCACCCCATCTTTGACCACGAGGTTACCCTGAGGTGCTGGGCCCTGGGCTTCTACCCTTCGGACATCACCCTGACCTGGCAGCGTGACGGGGAGGACCTGACCCAGGACATGGAGCTGGTGGAGACCAGGCCTGCAGGGGACGGGACCTTCCAGAAGTGGGCGGCCATGGATGTGCCCCCTGGGGAGGAGCAGAGATACACGTGCCAAGTGCAGCACCAGGTGCTGCCCAAGCCCCTGACCCTGAGATGGGACCCCCCTCCTCAGACCACCATGACCACCGTGGGCATCGCTGCTGCCGCCCTGGGTCTCCTTGGAGCTGTGGCCGCTGGAGCTGTGCTGTGGAGGAGGAGGCGCTCAGGCAGAGGCAGGGAGAGATACTCTCAGGCTGCCTGCAGTGACAGTGCCCAGGGCTCTCATGTGTCTCTCACGGCTCCTAAG

>Dero_G25_NW020093387_1088207_1090841

ATGGAGTCCCGACCACTGCTCCTGCTGCTCTCGGGGGCCCTGGCCCTGACCCAGACCTGGGCGGGCCCACACACCCTGAGACATTTCGGCACCTTACTGTCCGGACTCGGGGAGTCCCAGTATATCGGGGTCGGCTACGTGGACGACACGGAGGTCTTGCTGTTCGACAGCAACACCCCGAACCCGAGGGTGCAGCCGCGGATGCCGAGGAGGGAGCAGCCGTGGCTGGAGCAAGAAGACCCAGAGTTTTGGGCGGAGCAGACGTGGCTCTGGAAGCAGTGTGAACAGATTTCCCGAGCAAAGCTGAACAACCTGCGCGACTACTACAACCAGAGCAAGGCGCGGTCAGGGTTTCACACCTTCCAGGTCATTTCTGGCTGCGTTGTGGGGTCAGACGGGCGCTTCCTCCGCGGGTTCGGTCACACCGCGTACGACGGAACCGAAACCTTCACCCTGAAATTGGAGCTGAGTCCCACCACCGAAGCCAACAAGACCACCCAGATCACCGTGCAGCGCGATTTGTTCCATCAGGACGATGTGGAGGGTTGGAGCGCCTACGTGGAGAACACCTGCGTGCACCGGCTTCGCCTGTTCCTGGAGAAGAGGAAGGAGACGCTGCTCCGAACAGACCCGCCAAAGACACACGTGACCCACCACCCCATCTCTGACCACGAGGTCACCCTGAGGTGCTGGGCCCTGGGCTTCTACCCTGCGGACATCACCCTGACCTGGCAGCGTGATGGGGAGGACCTGACCCAGGACATGGAGCTTGTGGAGACCAGGCCCGCAGGGGACGGGTCCTTCCAGAAGTGGGCGGCTGTAGCCATGCCCCCCGGGGAGGAGCAGAGATACACATGCCGTGTGCAGCACCAGGCTCTGCCTGAGCCCCTGATTCTGAGATGGGACCCCCCTCCTCAGACCACCATGACCACCGTGGGCATCGCTGCTGCCGCCCTGAGTCTCCTTGTAGCTGCTGCCGCTGGAGCTGTGCTGTGGAGGAGGAGGCACTCAGGCAAAGGCAGGGAGAGATACTCTCAGGCTGCCTGCAGCGACAGTTCCCAGGGTTCTGATGTGTCTCTCACGGCTCCTAAG

>Dero_G10_pr_NW020093387_1029300_1032462

GGCCCCCACACCATAAGATATTTCAGCACGGTGGTGTCCAGACCCAGCATAAGGAAGCCCTGGTACATAGGTATTGCCTACTTGGACGACACGGAAATTGCCCGGTACGACAGTGAAGCGCCTAGTCCAAAGCTGAAGCCTCAGGTACCGTGGCTGGAGCAGCCGTGGGTGGAGCAGGAGCACCCACATTTTTGGGACGACTACACGACAGACATCAAGAACTACGAACAGATGTACGGAGGGAACCTGAACAACCTGCGCGCCTACTACAACCAGAGCAAGGACGGTGAGCACACCTTCCAGGAAATGAGTGGCTGCATCGTGGGGTTGGACTGGCACTTCCTCCGCGGATACACTCAGTTTGCCTATGACGGCACCGACTACATCGCCCTGAACGAGGACCTGAGCTCCTGGACCACTGCCGACACAGCGGCCCGGATCACCTGGCGCAACTTGGTGCAGGTTGAAGATGCGGACCACTGGAGGAGAATCCTAGAGAGCACGTGGGTGCACTGGATCCACCTGTTCCTGGAGAATGAAAAGGAGATGCTGCTCCGAGCAGACCCTCCAAAGACACACGTGACCCACCACCCCATCTCTGACCACGAGGTCACCCTGAGGTGCTGGGCCCTGGGCTTCTACCCTGCGGACATCACCCTGACCTGGCAGCGTGACGGGGAGGACCTGACCCAGGACACAGAGCTTGTGGAGACCAGGCCTGTGGGGGATGGAACCTTCCAGAAGTGGGCAGCCGTGGCTGTGCCCCCTGGGGAGGAGCAGAGATACACATGCCGTGTCCAGCATGAGGCGCTGCCTGAGCTCCTGACCCTGAGATGGGACCCCCCTCCTCAGACCACCATGTTATTTTTAAACATAGTCAAAGGG

>Dero_G3_NW020093387_1041111_1043901

ATGGGCCCCAGAACTCTGCTCCTGCTGCTCTCGGAGGTCCTGGCCCTGACCCAGACCTGGGCCGGCCTCCACAACGTGAGAATTTTTAGCACCTCCGTGTCTGGACCGGGCCGCGGGAAGAACCGGTACGTGGTGGTGGGCTACGTGGACGACACCGAGATCTTGCGGTTCGACAGCGACACCGCCAGGCCGAGGCTGGAGCCGCGGGTACCGTGGAGGGAGCAGCCGTGGGTGGAGCAGGAGCACCCACATTTTTGGGATGAGAAGACGCGGGTCTGCAAGTACAACCAACAGACTTTCCGAGCAAACCTAAACAGCCTGCGCCACTACTACAACCAGAGCGAGGATGGGTCAGGGTCTCACACCTACCAGGAAATGAGTGGCTGCGTCTTGGAGTCCGACTCCAGATTCCTGCGTGGGTTCAGTCAGTTCGCCTATGATGGAACTGACTACATTACGCTGAACGAGGACCTGCGCTCCTGGACCGCTGCGGACACGGCAGCTCGGATCACCTGGCGCAACTTGGTGCAAGTGCCTGATGTGGAGCGACGGAGGCTCGCCATTGGAGACTCCTGCGAGCGCTGGCTCCGCCTGTTCCTAGAGAACGGGAAGGAGATGCTGCTCCGAGCAGACCCTCCAAAGACACACGTGACCCACCACCCCATCTCTGACCACGAGGTCACCCTGAGGTGCTGGGCCCTGGGCTTCTACCCTGCGGACATCACCCTGACCTGGCAGCGTGACGGGGAGGACCTGACCCAGGACACAGAGCTTGTGGAGACCAGGCCTGTGGGGGATGGAACCTTCCAGAAGTGGGCAGCCGTGGCTGTGCCCCCTGGGGAGGAGCAGAGATACACATGCCGTGTCCAGCATGAGGCGCTGCCTGAGCTCCTGACCCTGAGATGGGACCCCCCTCCTCAGACCACCATGACCATCGTGGGCATTGCTGCTGCTGCCCTGGGTCTCCTTGTAGCTGTGGGCGCTGGAGCTATGCTGTGGAGGAGGAGGCACTCAGGCAGAGGCAGGGAGAGGTACTCTCAGGCTGCCTGCAGCGACCGTGCCCAGGGCTCTGATGTGTCTCTCACGGCTCCTAAG

>Dero_G23_NW020093387_949220_952015

ATGGAGCCCCAAACGCTGCTCCTGCTGCTCTCGGGGGCCCTGGCCATGACCGAGACCTGGGCCTGCCCCCACGCACTGAAATATTTCAGCACCATCGTGTCCGGACCTGGCCGCGGGAAGTACCGGTACATCGTCGTTGGCTACGTGGACGACACCGAGGTCGTGCGGTTCGACAGCGACGGCGCGAGCTCGAGGCTGGAGCCGCGGGTGCCGTGGCTGGAGCAATGGTGGGCGGAGCACGGGGATCCACATTTTTGGGAAGAGCAGACCCACGCCGTGAAGGACTACGGGCAGAGTTTCCGAGCCATCCTGAACAACCTGCGCGCCTACTACAACCAGAGCGAGGACGGGTCTAACACCTTCCAGGATATGAGCGGCTGCGTCGTGGGGTTGGACAGGAGCCTTCTCCGCTTGTACAGTCAGTACGCCTACGACGGCACTGAATACATCGCCCTTAACGTGGACCTGAGCTCCGACACAGCGGCGGACAAGGGGGTCCGGATCACCGCCAATCGCAATTTGGTGCAGAATCCTAACGTGGAAGGCTGGAAGCTCTGCCTCGAGGGCCTTTGCGAGTCCTGGCTCCACCTGTTCCTGGCGAAGGGAAAGGAGACGCTGCTACGAGCAGACCCACCAAAGTCACACCTTACCCACCACCCCATCTCTGACCATGAGGTCAACTTGAGGTGCTGGGCCCTGGGCTTCTACCCTGCGGACATCACCTTGACCTGGCAGCGTGACGGGGAGGACCTGACCCAGGACATGGAGCTGGTGGAGACCAGGCCTGCGGGGGATGGGACCTTCCAGAAGTGGGCAGCCGTGGTCGTGCCCCCTGGGGAGGAGCAGAGATACACGTGCCGTGTGCAGCACCAGGCGCTGCCTGAGCCCCTGACCCTGAGATGGGACCCCCCTTCTCAGACCACCATGACCACCGTGGCCATCGCTGCTGCCGCCCTGGGTCTCCTTGGAGCTGCAGCCGCTGCAGCTGTGCTGTGGAGGAGGAGGCGCTCAGACAGGGACAAGAAGAGCTACTCTCAGGCTGCCTGCAGCGACAGTGCCCAGGGCTCTGATGTGTCTCTCACAGCTCCTAAG

>Dero_G15_NW020093387_1016593_1019527

ATGGAGCACCGACTCCTGCTCCTGCTGCTCCCGGGGGCCCTGACCCTGACCGAGACCTGGGCCGGTCCCCACACCATGAGAATTTTCAGCACTGTTCTGTCCGGACCGGGCCCCGGGAAGACCCGGTACATCAGCGTCTATTACGCGGACGACACGGAGATCATGCGGTTCGACAGCGAGGCCCCGAATCCGAGTGTGGAGCCGCGGGTACCCTGGATGGAACAGGCATGGGTGGAGAAGCAGATGCCGGGCTATTGGAACGAGCGGACAGGGGTTTGCCAGCGCGAGGCACAGATTAACGAAGGGAACCTGAACAAGCTGAGCGCCAACTACAACCAAAGCGATCATGGCTCTCACACCTTACAGAAAATAAGTGGCTGTGTCGTGGGGTCCGACGGGCGCTTCCTCCGCGGGCACAGTCAGATCGCCTACGACGGCACCGACTACGTCGCCCTGAACCTGGACCTGCGCTCCTGGACCACGGCCAACTCTGCGGCCCATATCACCTCGCACAACTTGGTGCAGATCCCTTATGCGGAGACCTTGAAGGTCTTCCTAGAGGGCACTTGTGTGCACTGGCTACACCTGTTCCTGGAGAAGGGGAAGGAGACGCTGCTCCGAGCAGACCCTCCAAAGACACACGTGACCCATCACCCCATCTCTGACCACGAGGTCACCCTGAAGTGCTGGGCCCTGGGCTTCTACCCTGCGGACATCACCCTGACCTGGCAGCGTGATGGGGAGGACCTGACCCAGGACATGGAGCTTGTGGAGACCAGGCCTGGGGGGGACGGGACCTTCCAGAAGTGGGCGGCCATAGCTGTGCCCCCCGGGGAGGAGCAGAGATACACGTGCCGTGTGCAGCACCAGGCGCTGCCCCAGCCCCTGACCCTGAGATGGGACCCCCCTCCTCAGACCACCATGACCACCATGGGCATCATCACTGCTGCCGCCCTGAGTCTCCTTGTAGCTGGGCCGCTGGAGCTGTGCTGTGGAGGAGGAGGCGCTCAGGCTGCCTACAGCGACAGTGCCCAGGGCTCTGATGTGTCTCTCACAGGTCCTAAAGGT

>Dero_G19_NW020091182_20339478_20342485

ATGCGGACCGTGAGTTCCCTAGCCGTCCTTCTCCTCTTCGAGGCCGTGGCGTGGATCGAGGCCTGGGCTGGTCCTCACACCCTGAGATATTTCTACACCGTCATGTCCCCGCCGGGCGGCGGGGAGACCCACTTCGTTGCCGTCGGCTACGTGGACGACACGCAGTTCGTGCGGTTCGACAGCCGCTACGTGAATGCGAGGATGGAGCCGCGGGCGCCGTGGATGGAGGGGCCGTGGGTGGAGGAGGAGGACCCACAGTATTGGGACGTAGAGACGAGGAATGTTAAGGAAACCGCACAGAATTTCCGAGTGAACCTGAACAAACTGCGCTCTCACTACAACCAGAGCCTACACGAGCCTCACACCTTCCAGTGGACCTACGGCTGCGAGATGGGATCGGATGGCAGCTTCCTCCGCGGTTTTGACCAGTTCGCGTACGACGGCGCCGACTATATCACTTTGAACGAGGACCTGAGCTCTTGGACCGCGGCCCGCGCGGAGGCTCAGGTGTCCCAGCGCATATATGAAGCTATGCATGAGGCGGAGGTCCAGAGGCACTACCTGGAGAGGGAGTGCATAAAGTGGCTCAAAAGATACCTGGAAAAAGGGAAGGAGTCTCTGCAGCGCTCAGATCCTCCAAAGACACATGTGACCCACCACCCCGCCTCCGAGCGGGAGGCCACCCTGAGGTGCTGGGCGCAGGGCTTCTACCCTGCGGACATCGCCCTGACCTGGCAGCGGGACGGGGAGGACCTGACCCAGTACATGGAGCTTGTGGAGACCAGGCCCGAGGGGGACGGGACCTTCCAGACGTGGGCGGCGGTGGTGGTGCCTCGAGGAGAGGAGCAGAGATACACGTGCCACGTGGTGCACGCGGGGCTGCCCGAGCCCCTGAGGCTGCAGTGGGAGCCGCCTTCTCAGACCATCAGCAGCATCATCATCATCGTGGGCATTGTCATTGGCCTGGCCCTGCTGGGAGCTGTGGCAGTTGCAGCTGTGATGTGGGCGAGGAGGCGCTCAGGTGAAAAAAGAGGGAGCTGCGCTTGGGCTGCCAGCAGTGACAGTGACCGGGGCTTGGATGTGTCTTTCATAGCTTCTAAA

>Dero_G1_NW020091182_17460997_17463512

ATGCGGGTCATGCGGTGTCCGGCTCTCCTGCTGCTGCTCTCGGCGGCGCTGCCGCTGACCGGTGCCGGGGCGGGTCCGCACTCCCTGAGGTATTTCGACACCGCCGTATCCCGGGCTGGCGGCGGGGAGGCGTGGTTCCTTGTAGCCGGCTACGTGGACGGCGCGGAGTTCGTGCGGTTCGACAGCGACGCTGCAAACGCGAGCATGGAGCCGCGGGAGCCGTGGATGGAGCTGGAGGAACCGCAGTATTGGGTCAGCGAGACGCAGAATGCTTGGAGAACCGCAAAGATTTACCGATTGAGCCTGAACAGCGTGCGCCTCTACTACAACCAGAGCGAGGACGGGTCTCACACTCTCCAGGTGATGTACGGCTGCGACGTCTGGCCGAACGGGACCGTCCTCCGCGGGTACGATCAGTCCGCCTACGACGGCGCCGACTACCTCGCCCTGAACGAGGACCAGCGCTCCTGGACCGCGGCCGACACGGCGGCTCAGAACACGCAGCGCAAGTGGGAGGCGGCCGGTGTGGCGGAGCGATTCGGGGCCTACGTGCGGGGCCCCTGCAGGGAGACGCTCCTGCGGTACCTGGAACACGGGAAGGAGACGCTGCAGCGCGCAGGTACTGGGCCGTCCTCCCACCCCCAACCCCCGGGCTGGGCTCCCACGGGGGACTGGACGCGGGACCAGGCCTTAACCCTGACCTGGCAGCGAGATGGGGTGGACCAGCCCCAGGACACACAGCTTGTGGAGACCAGGCCCTGGGATAACAGTACCTTCCAGACGTGGGCGGAGGTGGTGGTGCCACGTGGAGAGGAGCAGAGATACACGTGCCACGTGGTGCACGCGGGGCTGCCCGAGCCCCTGAGGCTGCAGTGGGAGCCGCCTGCTCAGACCATCAGCAGCATCATCATCATCGTGGGCATTGTCATTGGCCTGGCCCTGCTGGGAGCTGTGGCAGTTGCAGCTGTGATGTGGGCGAGGAGGCGCTCAGGAGAAAAAGGAAGGAGCTACACTCAGGCTGCCAGCAGTGACAGTGACCAGGGCTCGGATGTGTCTCTCACAGCTTGTAAA

>Dero_G26_pr_NW020091182_17477842_17478842

ATGCGCGTCTTGGGATGTCCGAGCCTCCTGCTGCTGCTCTCGGCGGCGCTGCCGCTAACCGGTGCCGGGGCGGGTCCGCACTCCCTGAGGTATTTCGACACCGCCGTGTCCCGGCCCGGCGGCGGGGAGGCCCGGTTCATCTCCGTGGGCTACGTGGACGGCACGGAGTTCGTGCGGTTCGACAGCGACGCCGCGAACCCGAGGATGGAGCCGCGGGCGTGGTGGATGGAGGGGCCGTGGGTGGAGGAGGTGGACCCGAAGTATTGGGACGAGCAGACGCAGGTCGCCCAGGGAAACGCACAGACTTTCCGAGTGAGCCTGCACAATGTGATCGGATACTACAACCAGAGCGAGCACGGGTCTCACACTCTCCAGAGGATGCACGGCTGCGACGTGGGGCGTGACGGGGCCCTGCTCCGCGGGTATCACCAGCTCGCATACGATGGAGCGGACTACATCGCCCTGAACGAGGACCAGCGCTCCTGGACCGCGGCCGACACGGCGGCTCAGAACACGCAGCGCAAGTGGGAGGCGGCCGGTGTGGCGGAGCGATTCGGGGCCTACGTGCGGGGCCTGTGCAGGGAGTGGCTGCTGCTGTACCTGGAACACAGGAAGGAGACCCTG

>Dero_G4_NW020093387_903665_906455

ATGGGGTGCAGACCGCTGCTCCTGCTGTTCTCGGGAGCCCTGGCCCTGATCGAGACCTGGGCCGGTCCCCACACGCTGACGGTATTCAGCACCGCTGTGTCCGGACCCGGCCGCGGGAAGTCCCGGTACATCGTCGTCGGCTACGTGGACCACACGGAGATCGTGCGGTTCGACAGCAACGCCGCAAGGCAGAGGCTGGAGCCGCGGGTACCATGGATGGAGCAGCCGTGGGTGGAGCTGGAGCATCCACATTTTTGGGACCAGAAGACGCGGGACATCAGAAACTATGAAAAGATATATGGAGGGAACGTGGACAACCTGCGCCACTACTACAACCAGAGCGAGGACGGGTCTCACACCTTCCAGGAAATGACCAGCTGTGTAGTGAGATCGGACAGGAGCTTCCTCCGTGGATACACTCAGTTCGCTTACGACGGCACCGACTACATCACCCTGAACGAGGACATGCGCTCCTGGACCGCAACCGACGCGGCGGCCCGGATCACCTGGCGCAAGTTGGTGGAGCTCCCTGCTGCAGAGCGGCGGCGGGGAGTCCTAGGGGACGTATGCGTGCGCTGGCTCCACCTGTTCCTAGAAAACGGAAAGGAGACGCTGCTAGGAGCAGACCCTCCAAAGACATACGTGACCCACCACCCCATCTCTGACCACGAGGTCACCCTGAAGTGCTGGGCCCTGGGCTTCTACCCTTCGGACATCACCCTGACCTGGCAGCGTGATGGGGAGGACCTGACCCAGGAAATGGACCTGGTGGAGACCAGGCCTGCGGGGGACGGGACCTTCCAGAAGTGGGCGGCTGTGGCCGTGCCCCCCAGGGAAGAGCAGAGATACACGTGCCGTGTGCAGCATGAGGCGCTGCCCAAGCCCCTGATTCTGAGATGGGACCCCCCTTCTCAGACCACCATCATCATCATGGGCATCGCTGCTGCTGCCCTGGGTCTCCTTGGAGCTGTGGCCACTGGAGCTGTGCTGTGGAGGAGGAGGCGCTCAGGCAGAAGCAGGGAGAGCTACTCTCAGGCTACCTACAGCGACAGTGCCCAGGATGCTGAGGTTTCTCTCACAGCTCCCAAC

>Dero_G7_NW020093387_1069869_1076834

ATGGAGCCCCGAACCCTTGTCCTGCTGCTCTCGGGGGCCCTGGCCGTGATCCTGAGCGAGAGCTGGGCAGACACCCACACTCTGCGAATTTTCAGCACCTTTGTGTCCAGACCGGGCCGCAGGAAGAACCGGTACATCGGCCTCAGCTACGTGGACCACACGGAGATCCTGCGGCTCGACAGTGGCATGCTAAACCCGAGGCTGGAGCCGCGAGTACCGTGGCTGGGGCAGCAGCTGGTGGAGCAGGAGGATCCACATTTGTGGGAGGAGCAGACGCGTATCTGGAAGGAGAACCAACACTGTTTTCGAGCGGCCCTGAACAACCTGCGCGCCTACTACAATCAGAGCGAAGACGTGTCGCACACCCACCAGGAAAGGAGTGGCTGCGTTGTGGGGTCGGACTGGAGCTTCCTCCGCGGGTACAGTCAGCTCGCCTACGACGGCACTGAGTACGTCGCCCTGAACCTGGACCTGCGCAACTTGACTGTGACTGATACCGCGACTGAGATCACTTGGCGTAATTTGTTACCAAACCTTGATGTGAACTCTTGGAGGATCAGCCTGGAGGACCTGTGCTGGCGCTGGTTCCATATCTTCCTGAAGAAGGGGAAGGAGACGCTGCTCCGAGCAGACCCTCCAAAGACACACGTGACCCACCACCCCATCTCTGACCACGAGGTCACCCTGAAGTGCTGGGCCCTGGGCTTCTACCCTGCGGACATCACCCTGACCTGGCAGCGTGACGGGGAGGACCTGACCCAGGACATGGAACTTGTGGAGACCAGGTCTGCGGGGGACGGGACCTTCCAGAAGTGGGCAGCCATGGATGTGCCTCCTGGGGAGGAGCAGAGATACACCTGCCGTGTGCAGCACCAGGCGCTGCCTGAGCCCCTGACCCTGAGATGGGACCCCCCTCCTCAGACCACCATGACCACTGTGGGCATCACTGCTGCCGCCCTGGGCCTCCTTGGAGCTGTGGCCGCTGGAGCTGTGCTGTGGAGGAGGAGGCGCTCAGGCAGATGCAGGGAGAGCTGCGCTCCTGCTGTCTACAGCGACAGTGCCCAGGGCTCTGATGTGTCTCTCACAGCTCCTAAG

>Dero_G11_NW020093387_1110690_1113676

ATGGGTTACCGACCTCTGGTCCTGCTGCTCTCGGGGGCCCTGGCCCTGACGGAGACCTGGGCCGGTTCCCACACCATGAGAATTTTCACCACCTTCGTGTCCGGACCGGACCGCGGGAAGTCCCGGTACATCGGCATCGTCTACGCGGACGACACGGAGATCCTGCGGTTCGACAGCGACGCCCCGAACCCGAGGCTGGAGCCGCGGGTGCAGTGGATGGAGCAGCCGTGGGTGGAGCAGGAGACACCGGGCTATTGGAAAGATCGGACCGGGGTTTGCCAGCGCTACGCACAGATTAACCAACAGAACGTGAACAAGCTGCGCGCCCACTACAACCAGAGCGAGGACGTGTCCCACACCTTCCAGGAAATGAGTGGCTGCGTGGTGGACCCGGACGGACGCTTCATCCGCGGGTACAGTCAATTAGCCTACGACGGCACCGACTACCTGGCTTTGAACGAGGACATGAGCTCCTGGACTGCCGCGGACACGGACACGATCACCGAGCGCAACTTGGTGCAGATCCCTGATGCGGAGAGCAAGAAGGCCTACCTGAAGAGAACGTGCGTGCGCCGGCTCCTCAAGTACCTGGAGAAGGGGAAGGAGACGCTACTCCGAGCAGACCCTCCAAAGACACACGTGACCCACCACCCCATCTCTGACCATGAGGTCACCCTGAGGTGCTGGGCCCTGGGCTTCTACCCTTCGGACATCACCCTGACCTGGCAGCGTGACGGGGAGGACCTGACCCAGGACACAGAGCTTGTGGAGACCAGGCCTGCAGGGGACGGGACCTTCCAGAAGTGGGCGGCCATGGACGTGCCCCCCGGGGAGGAGCAGAGATACACATGCCGTGTGCAGCACCAGGCGCTGCCCAAGCCCCTGATTCTGAGATGGGACCCCCCTCCTCAGACCACCATGACCACCGTGGGCATTGCTGCTGCTGCCCTGTGTCTCCTTGTAGCTGCAGCCGCTGCAGCTGTGCTGTGGAGGAGGAGGCGCTCAGACAGGGACAAGAAGAGCTACTCTCAGGCTGCCTGCAGCGACAGTGCCCAGGGTTCTGATGTGTCTCTCACAGCTCCTAAG

>Dero_G20_NW020091182_2270180_2272281

ATGTCTTCGCGACCGAAGCGGACTCACACTCTCCAGACCCGGAGGAAACCGGTCCTGAGGCCCCCAACCCTCCTACTGCTCTTGGGGGCCCTGGCGCTGAGCGAGACCCGGGCGGGCCGCCACTCGCTGAGATATTTCGACACCGCCGTGTCCGGGCCCAACCGCGGGGAATACCGGTACATCGCCGCCGGCTACGTGGACGACACGCAGTTCATGCGGTTCGACAGCTACGACAAGAGCCAGAGCCCGGCGCGGCCGCGGGTGCATGGGCCGTGGGCGGTGGAGGAGCCGCAGCATTGGGACCGGTACACGCAGAACGCCAGGGACAACGCACAGAATTTCCGAGTGAGCCTGAACAACCTGCGCGGCTACTACAACCAGAGCGACAACGCCGGTCACGACCCCCGTCCCCACGGACCTCCGGACGTCGCCCCGCGGCTCCGGGTCCGAGCTTCACCCCGAGGCTGCGGGCAGTTCGCCTACGACGGCGCCGACTACCTCTCTCTGAACGCGGACCTGCGCTCCTGGGCCGCGGCCGATGCTGCGGCTCGCATCACCCGGCGCAAGTGGGAGGAGGCGGGTGAGGCGGAGCACTACAGGGCCTACCTGGAGTGGGAGTGCGTGCAGTGGCTCCGCAGAAACCTGGAGAGGCTGCAGCGCGCAGATCCTCCAAAGACGCACTTGACCCATCACCCCATCTCTGAGCATGAGGTCACCCTGCAGTGCTGGGCCCTGGGCTTCTACCCTGCGGAGATCACCCTGACCTGGCAGTGTGATGGGGAGGATAAGACTGAAGACATGGAGCTTGTGGAGACCAGGCCTGCAGGGGACGGGACCTTCCAGAAGTGGGCGGCGGTGGTGGTGCCTCCTGGAGAGGAGCAGAGATACACATGCCATGTGCAGCACCAGGGGCTGCCCGAGCCCCTGACCCTGAGATGGGAGCCACCTTCTCAGGCCAAAATTGGCCTGGTCCTCCTTGGAGTTGTGGTCACTGGAGCTGTGGTCACTGGAGCTGTGGTCGCTGGAGCTGTGGTGTGGAGGAGGAAGCTGTCAGGT

>Dero_G21_ps_NW020090695_3072_6790

GGCATACACAGCGCCCAGCGCATGTTCGGCTGTAATATCCAGGAGGACAGCCTCTCCAGCAACTTCTGGCATTTCGGCTTTGATGGGCAGGACCACCTGTCCCTGGACTCAGAGACTCTGAGCTGGGTGTCAGCTGAGCCTGTGGCCATGCAGACCAAGCGCTTGTGGGATTCGGAGCGCTGCTACGCTGAGTATGACAAGGCCTACCTGGAAAGCCTCTGCCTCACCTCTCTGCACAGGTATCTGGAGCTGGGAAGCCAGATCCTCACCCGGAGAGAGCCACCCACAGTGCACGTGACAAGGCACACAACCCAGGACAGGAGTGCCACACTGAGGTGCTGGGCCCTGGGCTTCTACCCGCAGGATATCTCACTGAGCTGGTGGCTGGGGAAGCAGGAGCTGGCCCTGGAGACTGAGTATGTGGAGACCCGCCCTAGCGGGGACGGCACCTACCAGACGTGGGCCGCTGTACAGGTGCCAGCCGGGCAGGAGGCTGCGTACACCTGCCGTGTGCAGCACTCTGGCCTGAACCACGTGCTCACCGTGGCTTGGGAACCGCCCTCTAGTCAAGGACTCATTGCCATGGTTACCTGTGCCCCCCTCTTCTTGCTGGCCGTGGTAGTTGTGATCTTGACCAGGAGATACCTTCAAGGCAGGAACACGGAACCTTATGAACAAGCCCCAGGTGGAGAGGAACCCATATAA

>Dero_G16_ps_NW020093628_21433436_21434810

ATGCGGGTCATGGGGACCCCAAACCTCCTCCCGGGGGCTCTGGCCCTCACCAAGGCCTGGGCGGTCTCCCATTCCCTGAGCTATTTCAGCACCACTGTGTCCCTGCAGACTTTGGGGAGCCTTGGTACTTCGAAGTCAGCTATGTGGATGACATGCAGTTTGCAGTTCTTGCAGTTCCACCAATGTCCCGCAAGTCTGAGGATGGTGCCACGGGGGCCATGGGCGGAGCAGGAGGAGTCAGACTGTTGGGACTGGAACACGGGGATGGTCAAGAACAAGGCACAGATTGCCAAAGTGCAGCTCAACCACCTGCATGGCTACTACAACCAGAGCGATAACGGGTCTCACACTCTCCAGGGGCTGCCTGGATGTGACATGGCTTTGGATGGGCGCTTCCAGGTGACCCGTGTGGCAGAGCCCATCAAAAACTACCTGCAGGGCAGGTGCCTAGAGGGGCTCTTCAGATACCTGGAGAAGGGGAAAGAGATGCGGCTAGTTACAGATCCCCCAAATACATGTGTAACCCACCACCTCATCTCTGACCATGAGATCACCACCCTAAT!TGCTGGGTCCTGGGCTTCTACCCTGTAGAGATCACCCTGATCTGGCAGCATGGTGGGGAGGACCAGACCCAGGACATGGAG!TTGTGGAGACCAGGCCTGCAGGGACAGGAAC!TTCCAGAAGTGGGTGGCTGTGGTGGTGCAGTCTGGAGAGGAGCAGAGATACGAATGCCACGTGCAGCAGGAAGGG

>Dero_G22_ps_NW020090758_76_2543

GGCCCACACACCCTTACACTTCCCGTCACCTTCGTGTCCGGACTCGGGAGGTCCCAGTACCTAGTCGTCGGCTACGTGGACGACACGCAGATTTTGTGGTTCGACAGCGACTCCCCGAATCCAAGGGTGCAGCCGCGGGTGCCGTGGATGGAGCAGCCGTGGACAAAAATGACGAGGCTCTGCAAGCACTGGGAACAGATTTCCCGAGCAAAGCTGAACAACCTGCTAGATTCCTACAACCTGAGCAAGGCGGGGTCAGGGTCTCACACCTTCCAGGACATGAGTAGCTGCGTTGTGGGGTCGGACGGGGGCTTCCTCAGCGGGTACGGCCAGTACGCCTACGACTGCACTGAATCCTTCTCCCTGAACTTGGACCTGCGCTCATCCACCGGAGCCAACAAGACCGCCCAGATCACCGTTGGGCGAGATTTATTGCATCAAGATGATGTGGAGTGTTGGAGGGCCTATCTGGAGAACACGTGTGTGCGCTGGCTTCACCTGTTCCTGGAGAAGGGGAAGGAGAAGCTGCTAAGAGAAGACCCGCCAAAGACACACGTGACCCACCACCCCATCTCTGACCATGAGGTCACCCTGAGGTGCTGGGCCCTGGGCTTCTACCCTTCGGACATCACCCTGACCTGGCAGCGTGACGGGGAGGACCTGACCCAGGACATGGAGTTTGTGGAGACTAGGCCTGCGGGGGATGGAACC!A!GTGGATGTGCCCCCTGGGGAGGAGCAGAGATACACATGCCGTGTGCAGCACCAGGCGCTGCCTGAGCCCCTGACCCTGAGATGGGACCCCCCTCCTCAGACCACTGTGACCACCGTGGGCATCACTGCTGCCACCCTGGGTCTCCTTGGAGCTGTGGGTGCTGGAGCTGTGCTGTGGAGGAGGAGGCACTCAGGCAGAGGCAGGGAGAGCTGCACTCAGGCTGCCTGCAGTGACAGTGCCCAGGGCTCTGATGTGTCTCTCACAGCTCCTAAG

>Dero_G9_ps_NW020093387_1052490_1055903

GGACACACGCGGTCTCACAGAACATCAGCATCAGACACGGACATCCCGCGACCACGGCGACGACTCGGGGAGTCCCAGTACATTGGGGTCGCCTACGTGGACGACACGGAGATCGTGCGGTACGACACCAACACCCCGAACGCGAGGGTGCAGCCGCGAGTGCCATGGTTGGAGAGCCCGTGGGTGGAGCAGGAGTATCCAGATATGTGGGAGGAGCAGAAGCGAATCTGCAAGCAGTGTGAACAGATTTGCCGAGCAAAGCTGAACAACCTGCGCCATTACTACAACCAGAGCGAGGACGGGTTTCACAACTTCCAGGTTACTTCTGCCTGCGTTGTGGGGTCGGACCGGAGCTTCCTAAGAGGGTACAGTCACACCGCGTACGACGGAACCGAAACCTTCACCCTGAACTTGGACCTAAGTTCCACGACCGAAGCCAAGAAGACCACCCAGATCACCGTGCAGCGCGATTTGTTCCATCAGGACGATGTGGAGGGTTGGAGCGCCTACGTGGAGAACACCTGCGTGCACTGGCTCCGCCTGTTCCTGGAGAAGGGGAAAGAGACGTTGCTCCGAGCAGACCCTCCAAAGACACACGTGACCCACCACCCTATCTCTGACCATGAGGTCACCCTGAAGTGCTGGGCCCTGGGCTTCTACCCTTCGGACATCACCCTGACCTGGCAGCGTGACGGGGAGGACCTGACCCAGGACATGGAGTTTGTGGAGACTAGGCCTGCGGGGGATGGAACCTTCCAGAAGTGGGCAGCCGTGGCCATGCCCCCTGGGGAGGAGCAGAGATACACATGCCGCGTACAGCACCAGGCTCTGCCTGAGCCCCTGACCCTGAGATGGGACCCCCCTCCTCAGACCACCATGACCACCGTGGGCATCGCTGCTGCCGCCCTGGGTCTCCTTGGAGCTGCAGCCACTGGAGCTGTGCTGTGGAGGAGGAGGCGCTCAGGCAAAGGCAGGGAGAGATACTCTCAGGCTGCCTGCAGCGACAGTGCCCAGGGCTCTGATGTGTCTCTCACAGGTCCTAAG

>Dero_G5_ps_NW020093387_920223_922986

CTCTTGGGGTCCCGAACCCTCCTGCTGCTCTCCGGGACCCTGGCCCTGACCCAGACCTGGGCGGGCCCCCACACCCTGAGATATTTAAGCACCACCGTGTTCGAAACGGACAGCGGGAAATACCGGTACATCGTGGTCGCCTACGTGGACGACACGGAGATCTGGAGTTATGACAGCAACACAAGCAGCCCGAGGCTGCAGCCGCGCTTTCTGAGGAAGGAGCTGCTGCGGGGGGAGCGGGAGCATGCAAAATTTTGGAAGATTCAAGAGCGGGAAATAAAGCACAACGAACAGATGAGCAGAGCGAACCTGAACAGGCTGAGCGCGCACTACAATCAGAGCGACCGCGGCGGGGCTGGGCCAGGGCCCCACACTTGGCAGGAAATGACCGCCTGCGTCGTGGGATCGGACGGGACGTTCCTCAGCGGGTTTAGTCAGTTCGCCTACGACGGCACCGACTGCGTCGCCCTGAACCTGGACCTGCGCAACTGGACC!!TTTGGCAGGGATCTGCTGGAGAAACGACGTGTGGGTCCCTGATGCGGACGTCAGGAGGGCCTTCCTGGAGCACGGATGCGTGCACCAGCTCCACCTGCTCCTGGAGAAGGGGAAGGAGACGCTGCTCCGAGCAAACCCTCCAAAGACACACGTGACCCACCACCCCTTCTCTGACCACGAGGTCACCCTGAGGTGCTGGGCCCTGTGCTTCTACCCTTCGGACATCACCCTGACCTGGCAGCGTGATGGGGAGGACCTGACCCAGGACATGGACCTGGTGGAGACCAGGCCTGTGGGGGATGGAACCTTCCAGAAGTGGGCGGCCGTGGCTGTGCCCCCTGGGGAGGAGCAGAGATACACGTGCCAAGTGCAGCACCAGGGGCTGCCTGAGCCTCTGACCCTGAGATGGGACCCCCGTCCTCAGACCACCATGACCACCGTGGGCATCGCTGCTGCTGCCCTGGGCCTCCTTGTAGCTGCAGCTGCTGCAGCTGTGCTGTGGAGGAGGAGGCGCTCAGACAGGGACAAGAAGAGCTACTCTCGGGCTGCCTGCAGCAACAGTGCCCAGGGCTCTGATATGTCTCTCACGGTTCCTAAG

1. **Great roundleaf bat *Hipposideros armiger***

>Hiar_G8_pr_NW017732828_175780_178027

ATGGGCCCCGGAACCCTCCTCCTGCTGCTCTCGGGGGCCCTGGGCCTGACGGGGACCCGGGCGGGCTCCCACTCCCTGAGGTGTTTCGGCACCACCTGGTCCCGGCCCGGCCGCAGTGATCCTCGCTTTGTCGCCGTCGGCTTCGTGGACGACACGCAGTTCATGCGGTTCGACAGCGACGCGGCGAGTCCGAGGATGGAGCCGCGGGCGGCGTGGGTGCAGGGGCCGCGGGCGGAGCAGGCGCTCCCGGGGTACTGGGAGCAGAAGACAGCAGACCTCAAGGTGGTCGCACAGGCTGCCCGAGTGGAGCTGAACTTCCGGCGCAGCTACTACAACCAGAGCGAGGCAGGGTCTCACACCATCCAGTGGATATACGGCTGCGAAGCGGCGTCGGACGGGCGCCTCCTCCGCGGGTACACGCAGTACGCCTACGACGGCGCTGACTACCTCGCCCTGAACGAGGACATGCGCTCCTGGACCGCGGCCGACACGGCGGCTCAGATCACCCAGCGCAACTGGGATGCGGAGAGCCTGAGAGACTACTTTGAGGGCAGGTGCCTGGAGGGTCTCCTCAAATACCTGGAGATCGGGAAGGAGACGCTGCTGCGCGCAGACCCTCCAAAGACACACGTGACCCACCACCCCACCTCTGACCATGAGGTCACCCTGAGGTGCTGGGCGCTGGGCTTCTACCCTGCGGAGATCACCCTGACCTGGCAGCGTGATGGGGAGGACCTGACCCAGGACACAGAGCTCGTGGAGACCAGGCCTGCGGGGGACGGGACCTTCCAGAAGTGGGCAGCTGTGGTGGTGCCTCCTGGAGAGGAGCAGAGATACACGTGCCATGTGCAGCACGAGGGGCTGCCCGAGCCCCTGACCCTGAGATGGGAGCCGCCTCCTCAGGCCGCCATCCCTGTGGGCATCATTGCTGTGCTGGTCCTCCTTGGAGCTGCGGTCACTGGGGCTGTGGTGGCTGGAGCTGTGCCGTGGAGGAGGAAGCGCTCAGGTAGGGAAGGG

>Hiar_G89_ps_NW017732828_88033_90859

ATGGGCCCCGGGACCCTCCTCCTGCTGCTCTCTGGGGCCCTGGGCCTGACAGGGACCCGGACGGGCTCCCACTCCCTGAGGTATTTCTTCACTTCCTGGTCCCGGCCCGGCCGTGGGGAGCCCCGGTTCATCGCCGACGGCTTCGTGGACGACACGCAGTTCGCGCGGTTCGACAGCGACTCCGCGAGTCCGGGGGTGGAGCCGCGGGCGGCGTGGGTGGAGCAGGTGGAGCCTGGGTATTGGGACCCGGAGGCAGCAGAGGTGAAGGACATCGCACAGAATTTCCGAGTGGGGCTGAGCAATGTGCGCGGCTACTACCACCAGAGCGAGGCCGGGTCTCACACCCTCCAGGGGATAATTGCCTGCGACGTGGGGCTGGACGGGCGCCTCCTCCGCGGGTACACGCAGCTCGCCTACGACGGCGCTGACTACATCGCCCTGAACGAGGACCTTTCCTCCTGGACCGCGGCCGACAGGGCGGCTCAGATCACCCGGCGCAAGTGGGAGGTGGCCCATGTGGCAGAGCGCCTGAGGGACTACCTTGAGGGCAGGTGCGTGCAGGCCCTCGGCAAACACCTGGAGAAGGGGAAGGAGACGCTGCTGCGCGCAGACCCTCCAAAGACACACGTGACCCACCACCTCACCTCTGACCGTGACGTCACCCTGAGGTGCTGGGCGCTGGGCTTCTACCCTGCGGAGATCACCCTGACCTGGCAGCGTGATGGGGAGGACCTGACCCAGGACACAGAGCTGGTGGAGACCAGGCCTGCGGGGGATGGGACCTTCCAGAAGTGGGCAGCTGTGGTGGTGCCTCCTGGAGAGGAGCAGAGATACACGTGCCATGTGCAGCATGAGGGCCTGCCCGAGCCCCTGACCCTGAGATGGGAGCCGCCTCCTCAGGCCACCATCCCTGTGGGCATCATTGCTGTGCTGGTCCTTGGAGCTGTGGTCACTGGGGCTGTTGTGGCTGGAGCTGTGTTGTGGAGGAGGAGGCAT!!T!GAGGTGAAAAAGGAAGGAGCTACGCTGAGGCTGCAAGTGGTGACCGTGCCCAGGGCTCTGATGTGTCTCTCACAGCTTCTAAA

>Hiar_G48_NW017732828_38010_40971

ATGCGAATTATGGGGCTGGAAGCCCTCCTGCTGCTCTCGGGGGCCCTGGGCCTGACGGGGACCCGGGCGGGCTCCCACTCCCTGAGGTGTTTCGGCACCTCCTGGTCCCGACCCGGCGGGGAGCCCCGGAACATCGCTGTCGGCTACGTGGACGACACGCAGTTCGCTCGGTTCGACAGCGACGCCGCGAGTCCGAGGGTGGAGCCGCGGGCGGCGTGGGTGGAGCAGATGGACCCGGGGTACTGGGACCAGGAGACAGCAACCGCCAAGGCGGCCGCACGGGATGCCCGAGCGGAGCTGAACTTCCGAAGCAGCTACTACCACCAGAGCAAGGCCGGGTCTCACACCCTCCAGGCGATATTTGCCTGCGACGTGGGGCCGGACGGGCGCCTCCTCCGCGGTTACTGGCAGCTCGCCTACGACGGCGCTGAGTACCTCGCCCTGAACAAGGACCTGCGCTCCTGGACCGCGGCCGACAGGGCAGCTCAGATCACCCGGCGCAAGTGGGAGGCGGCCGGTGTTACGGAGCTTCTGAGGGGTTTCCTTGAGGGCAGATGCGTGGAGTCGCTCCGCAAATACCTGGAGAAGGGGAAGGAGACGCTGCTGCGCGCAGACCCTCCAAAGACACACGTGACCCACCACCCCACCTCTGACCGTGAGGTCACCCTGAGGTGCTGGGCCCTGGGCTTATACCCTGCGGAGATCACCCTGACCTGGCAGCGTGATGGGGAGGACCTGACCCAGGACACGGAGCTCGTGGAGACCAGGCCTGCGGGGGACGGGACCTTCCAGAAGTGGGCAGCTGTGGTGGTGCCTCCTGGAGAGGAGCAGAGATACACGTGCCATGTGCAGCACGAGGGGCTGCCCGAGCCCCTGACCCTGAGATGGGAGCCGCCTCCTCAGGCCGCCATCCCTGTGGGCATCATTGCTGTGCTGGTCCTTCTTGGAGCTGCGGTCACTGGGGCTGTGGTGGCTGGAGCTGTGCTGTGGAGGAGGAGACTCACAGGTGGAAAAGGATGGAGCTACGCTCAGGTTGCAAGCAGTGACATTGCCCAGGGCTCTGATGTGTGTCTCACGGCTTCTAAAGGT

>Hiar_G91_NW017732873_120699_123720

ATGCGCATCATGGGCGCCGGGACCCTCGTCCTGCTGCTCTCGGGGGCCCTGGGCCTGACGGGGACCCGGGCGGGCTCCCACTCCCTGAGGTATTTCGGAACCACCTGGTCCCGGCCCGGCGGGGAGCCCCGCTTTGTTGGCGTCAGCTACGTGGACGACACGCAGTTCGCGCGGTTCGACAGCGACGCCACGCTTCAAAAGGTGGAGCCGCGGGCGGCGTGGGTGGAGCACGTGGAGCCGGGGTTCTGGGACCGGGAGACAGCAGACGTCAAGGTGGTCGCACAGGCTGTCCGAGCGGAGCTGAAGTTCCGGGGCAGCTACTACCACCCGAGAGATGCTGGGTCTCACACCATCCAGGGGATGTCTGCCTGCGACGTGGGGCCGGACGGGCGCCTCCTCCGCGGGTACTGCCAGCTCGCCTACGACGGCGCTGAGTACATAGCCCTGAACGAGGACATGCGCTCCTGGACCGCGGCCGACGCGGCGGCTCAGATCACCCAGCGCAAGTGGGATGCGGCCGGTTGGACAGAGCGCAAGAGGGCCTACCTTGAGGACAGGTGCCCGGAGTTGCTCCGCAGATACCTGGAGAAGGGGAAGGAGACGCTGCTACGCGCAGATCCTCCAAAGGCACACGTGACCCACCACCCCAGCTCTGACCGTGACATCACCCTGAGGTGCTGGGCTCTGGGCTTCTACCCTGCGGAGATCACGCTGACCTGGCAGCGTGATGGGGAGGACCTGACCCAGGACACGGAGCTCGTGGAGACCAGGCCTGCGGGGGACGGGACCTTCCAGAAGTGGGCAGCTGTGACGGTGCCTCCTGGAGAGGAGCAGAGATACACCTGCCATGTGCAGCACGAGGGGCTGCCCAAGCCCCTGACCCTGAGATGGGTTCCAGAGCCGCCTCCTCAGGCCGCCATCCCTGTGGGCATCATTGCTGTGCTGGTCCTTGGAGCTGTGGTCACTGGGGCTGTGGTGGCTGGAGCTGTGCTGTGGAGGAGGAGGCTCACAGTTGGAAAAGGAGGGAGCTACGCTCAGGCTTTAAGCAGTGACAGTGCCCAGGGCTCTGATGTGTCTCTCAAGGCTTCTAAAGGT

>Hiar_G49_pr_NW017732873_145662_147872

ATGGGCCCCGGGACCCTCGTCCTGCTGCTCTCGGGGGCCCTGGGCCTGACGGGGACCCGGGCGGGATCCCACTCCCTGAGGTGTTTCGGCACCACCTGGTCCCGGCCCGGCCGCGGGGAGCCTCGCTTTGTCGCCGTCAGCTACGTGGACGACATGCAGTTCATGCGGTTCGACAGCGACGCGGCGAGTCCGAGGGCGGAGCCGCGGGCGGCGTGGGTGGAGCACGTGGAGCCGAGGTTCTGGGAGCGGAAGACAGCGGACCTCAAGGCGGCCGCCCGAGTTGCCCAAGCGGAGCTGAAGTTCCGGCGCGGCTACTACAACCAGAGCGAGGCCGGGTCTCACACCATCCAGTGGATATACGGCTGCGAAGCGGGGTCGGACGGGCGCCTCCTCCGCGGGTACACGCAGTTCGCCTACGACGGCGCTGACTACATCGCCCTGAACGAGGACCTTTCCTCCTGGACCGCGGCCGACACAGCGGCTCAGATCACCCAGCGCAACTGGGAGGCGGCTGGTGCGGCGGAGCGCCTGAGGGGCTACTTTGAGGGCAGGTGCCTGGAGGGTCTCCTCAAATACCTGGAGATCGGGAAGGAGACGCTGCTGCTCGCAGACCCTCCAAAGACACATGTGACCCACCACCCCACCTCTGACCGTGAGGTCACCCTGAAGTGCTGGGCGCTGGGCTTCTACCCTGCGGAGATCACCCTGACCTGGCAGCGTGATGGGGAGGACCTGACCCAGGACACAGAGCTCGTGGAGACCAGGCCTGCGGGGGACGGGACCTTCCAGAAGTGGGCAGCTGTGGTGGTGCCTCCTGGAGAGGAGCAGAGATACATGTGCCATGTGCAGCACAAGGGGCTGCCTGAGCCCCTGACCCTGAGATGGGAGCCGCCTCCTCAGGCCGCCATCCCTGTGGGCATCATTGCTGTGCTGGTCCTCCTTGGAGCTGTGGTCACTGGGGCTGTGGTGGCTGGAACTGTGCCGTGGAGGAGGAAGCGCTCAGGTAGGGAAGGG

>Hiar_G21_pr_NW017732523_25812_28278

ATGCGGGTCAAGTTGCCAGGAACCCTCCTCTTGCTGCTCTCGGGGGCCCTGGCTCTGACTGGGACCCTGGCGGGCTCCCACTCCCTCAGGTATTTCGTCACCACCTGGTCCCGGCCCGGCCGCGGGGAGCCCCGGTACATCGTCGTCGGCTACGTGGACGACACACAGTTCATGCGATTTGACAGCGACGCCGCGAGTCCAAAGGCGGAGCCGCGGGCGGCGTGGATGGAGGGGCCGTGGCTGGAACAGGTGGACCCGGGGTACTGGGAGCGGGAGACAGGACGCGCCAAGTTCTACCAACAGGTTTTGCGAGCGGACCTGCAGAGCGCGCTGGAGAACTACAACCAGAGCGAGGCCGGGTCTCACACCTACCAGAGGATGTGTGGCTGCGACATGGGACAGGACGGGCGCCTCCTCCGCGGGTACAGGCAGGACGCCTATAACGGCGCTGACTACGTCGCCCTGAACGAGGACATGCGCTCCTGGACCGCGGCCGACACGGCGGCTCAGATCACCCGGCGCAAGTGGGAGGCGGCCGGTGAGGCAGAGCGCCTGAGGGGCTACTATGAGGGCACCTGCATGGAGTGGCTCCTCAGATACCTGGAGAACGGGAAGGAGATGTTGCTGCGCGCAGATCCTCCAAAGACACATATCACTCACCACCCCATCTCTGACCGTGACGTCACCCTGAGGTGCTGGGCGCTGGGCTTCTACCCTGCGGAGATCACACTGACCTGGCAGCTTGATGGGGAGGACCTGACCCAGGACATGGAGCTCGTGGAGACCAGGCCGGCAGGGGATGGGACCTTCCAGAAGTGGGTGTCTGTGGTGGTGCCTTCTGGAGAGGAGCAGAGATACACATGCCATGTGCAGCACAAGGGGCTGCGCAAACCCCTCACCCTGAGATGGGAACCACCTCCTCAGCCCACCATCCCAATTATGGGCATCATTGTTGGTCTGGTTCTCTTTGTGGTCACTGGAGCTGTGGTGCTTGGAGCTGTGCTGTGGAGGAAGAAACACTCAGGTAAGGAAGGAGTGAGGATTGAG

>Hiar_G1_NW017732523_122669_125670

ATGCAGGTCAATGGGCTGGGAACCCTCCTCCTGCTGCTCTCGGGGGCCCTGGCCCTGACGGGGCCCCGGGCGGGCTCCCACTCCCTGAGGTATTTCGTCACCACCTGGTCCCGGCCCGGCAGCGGGGAGCCCCGGTTCATCGGCGTCGGCTACGTGGACGACACGGAGTTCGTGCGATTTGACAGCGACGCCGCGAGTCCAAAGACGGAGCCGCGGGCGGCGTGGATGGAGACGCCGTGGCTGGAGCAGGTGGACCCGGGGTACTGGGAGCGGGAGACACAACGCGCCAAGGACCACCAACAGGTTTTGCGAGCGGAGCTGCAGAGCGCGCTGGAGAACTACAACCACAGTCAGGCCGAGTCTCACACCTACCAGAGAATATGTTGCTGCGAAGTGGGGCCAGACGGGCGCTTCCTCCGCGGGTACGCGCAGTACGCCTACGACGGCGCTGACTACATCTCCCTGAACGAGGACCTTTCCTCCTGGACCGCGGCCGACAAGGCAGCTGAGATCTACAAGAAGAAATTGGAGGCAGCTGGTGTAGCGGACAACGCGAGGGGCTACTTTGAGGGCACCTGCGTGGAGTGGCTCCTCAGATACCTGGAGAACGGGAAGGAGACGCTGCTACGCGCAGATCCTCCAAAGACACATATCACTCACCACCCCATCTCTGACCGTGACGTCACCCTGAGGTGCTGGGCGCTGGGCTTCTACCCTGCGGAGATCACCCTGACCTGGCAGCTTGATGAGGAGGACCTGACCCAGGACATGGAGCTCGTGGAGACCAGGCCGGCGGGGGACGGGACCTTCCAGAAGTGGGTGTCTGTGGTGGTGCCTTCTGGAGAGGAGCAGAGATACACATGCCATGTGCAGCACAAGGGGCTGCGCAAACCCCTCACCCTGAGATGGGAACCACCTCCTCAGCCCACCATCCCAATTATGGGCATCATTGTTGGTCTGGTTCTCTTTGTGGTCACTGGAGCTGTGGTGCTTGGAACTGTGCTGTGGAGGAAGAAACACTCAGGTGAAAAAGGAGGGAACTACACTGAGGCTTCAAGCAGTGACAGTGCCCAGGGCTCTGATGTTTCTCTCACAGCTTTAAAGGTG

>Hiar_G34_NW017732037_1045931_1048838

ATGGGCCCCGGGACCCTCGTCCTGCTGCTCTCCGGGGCCCTGGGCCTGACGGGGACCCGGGCGGGCTCCCACTCCCTGAGGTATTTCTTCACTTCCTGGTCCCGGCCCGGCGGCGGGGAGCCCCGGTTCATCTCCGTCGGCTACGTGGACGACATGCAGTTCGTGCGGTTCGACAGCGACGCCGCGAGTCCGAGGGCGGAGCCGCGGGCGGCGTGGATGGAGGGGCCGTGGGTGGAGCAGGCGCTGCCGGGGTACTGGGAGCGGAACACGCAGTTATACGAGGACACCGCACAGACTGACCGAGTGAGCCTGCACACCCTGCGCCGCTACTACAACCAGAGCGAGGCCGGGTCTCACACCATCCAGAGGATGTCCGGCTGCGACGTGGGGCCGGACGGGCGCCTCCTCCGCGGGTACTGGCAGGCCGCCTACGACGGCGCTGAGTACATCGCCCTGAACGAGGACCTTTCCTCCTGGACCGCGGCCGACACGGCGGCTCAGATCTCCCGGCGCAAGTGGGAGGCGGCCGGTGGGGCGGAGAGCCTGAGGGGCTACTTTGAGGGCCTGTGCCTGGAGTGGCTACGCAAATACCTGGAGAAGGGGAAGGAGACGCTGCTGCGCGCAGACCCTCCAAAGACACACGTGACCCACCACCCCAGCTCTGACCGTGGCGTCACCCTGAGGTGCTGGGCTCTGGGCTTCTACCCTGCGGAGATCACCTTGACCTGGCAGCGTGATGGGGAGGACATGACCCAGGACACGGAGCTCGTGGAGACCAGGCCTGCAGGGGATGGGACCTTCCAGAAGTGGGCAGCTGTGGCGGTGCCTCCTGGAGAGGAGCAGAGATACACGTGCCATGTGCAGCACGAGGGGCTACCCGAGCCCCTGACCCTGAGATGGGAGCCACCTCCTCAGGCCACCAACTCCATTGGCATGGTGATGGGCATCATTGTTGGGGTGCTGGTCCTCCTCGGAGCTGTGGTGGCTGGAGCTGTGCTGTGGAGGAGGAGGCTCTCAGGTGGAAAAGGATGGAGCTACGCTCAGGCTACAAGATGTGACAGTGCCCAGAGCTCTGATGTGTCTCTCACGGCTTCTAAA

>Hiar_G25_pr_NW017732037_1112900_1114038

ATGGGTCCTCGAACGCTGCTCCTGCTGCTCTCGGGGTCCCTGGCCCTCACCGCGACCTGGGCGGGCTCCCACTCCCTGAGATATCTCCACTCCGCCGTGTCCCGGCCCGGCCGCGGGGAGCCCCTCTACATCTCCGTCGGCTACGTGGACGACACGCAGTTCCTGCGGTTCCACAGCGACGCCGCGCTTCCGAAGGTGGAGCCGCGCGCTCCGTGGGTGGAGCAGGAGGGGCCGCAGTTTTGGGAAGCTCAGACGGAGATCGCCAAGGTCCACGCGCAGACTTCGCGATCGAACCTGGAGACGGCCCGTGGCTACTACAACCAGAGCGAGTCTGGGTCTCACACCTTCCAGTGGACTTCTGGCTGCGACGTGGGGCCGGACGGGCGCTTCCTCCGCGGCTATGAGCAGTTCGCCTACGACGGCGCTGACTACATCGCCCTGAATGAGGACCTGCGCTCGTGGAACGCGGCGGACGAGGTGGCACACATCACCCGTCGCAAGTGGGAAGCGGCCGGATTAGCTGAGCGCTACCGGGCATACCTGGAGAGGGAGTGCGTGGAGTGGCTCCGCAGGTACCTGGAGAACGGGAAGGAGACGCTGCAGCGCGCA

>Hiar_G62_NW017733229_36470_46565

ATGGGCCCCGGGACCCTCCTCCTCCTGCTGCTCTCGGGGGCCCTGGGCCTGACGGGGACCCGGGCGGGCTCCCACTCCCTGAGGTATTTCGAGACCACCTGGTCCCGGCCCGGCCGCGGGGATCCCCGGTTCATCGCCGTCGGCTACGTGGACGGCACGGAGTTCCTGCGGTTTGACAGCGACTCTGCGAGTCCGAGGATGGAGCCGCGGGCGGCGTGGATGGAGGGGCCGTGGGTGGAGCAGACGTGGCCGGGGTACTGGGAGCGAGATGTTGGAGCTGAGATGCGTGTTAGAAATACAATGAGGTCATCGCACTGGCAGTTGGGCAGGTTCCGGCGCCATCACTGCGGGGCGGGGTCAGGGTCTCACACCATCCAGGCGATATGTGGCTGCGACGTGGGGCCGGACGGGCGCCTCCTCCGCGGTTACAGGCAGCATGCTTACGACGGCGCTGAGTACATCGCCCTGAACGAGGACATGCGCTCCTGGACCGCGGCCGACACGGCGGCTCAGATCACCCGGCGCAAGTGGGAGGTGGCCCATGTGGCGGAGAGCCTGAGGGGCTACTTTGAGGGCAGGTGCCTGGAGTCGCTCCGCAAATACCTGGAGAAGGGGAAGGAGACGCTGCTGCGCGCAGACCCTCCAAAGACACACGTGACTCACCAGCCCACCTCTGACCGTAACGTCACCCTGAGGTGCTGGGCGCTGGGCTTCTACCCTGCGGAGATCACCCTGACCTGGCAGCGTGATGGGGAAGACCTAACCCAGGACACGGAGCTCGTGGAGACCAGGCCTGCGGGGGACGGGACCTTCCAGAAGTGGGCAGCTGTGGCGGTGCCTCCTGGAGAGGAGCAGAGATACACGTGCCATGTGCAGCACAAGGGGCTGCCCGAGCCCCTGACTCTGAGATGGGAGCCGCCTCCTCAGGCCGCCATCCCTGTGGGCATCATTGCTGTGCTGGCCCTTGGAGCTGCGGTCACTGGGGCTGTGGTGGCTGGAGCTGTTCTGTGGAGGAGGAAGCGTCACAGTTCTGGTGGAAAAGGAGGGAGCTACGCTCAGGCTGCAGGCAGTGACAGTGCTCAGGGCTCTGCTGTGTGTCTCACGGCTTCTAAAGGT

>Hiar_G56_NW017732984_97854_100704

ATGCGCATCATGGGCCCCGGGACGCTCGTCCTGCTGCTCTCGGGGGCCCTGGGCCTGACGGGGACCCGGGCGGGCTCCCACTCCCTGAGGTATTTCTTCACCTCCTGGTCCCGGCCCGGCCGTGGGGAGCCCCGGTTCATCGCTGTCGGCTACGTGGACGACACGCAGTTTGCACGGTTCGACAGCGACGCCGCGAGTCCGAGGGTGGAGTCGCGGGCGGCGTGGATGGAGCAGGTGGAGCCGGGGTTCTGGGACCGGGAGACAGCAGACGCCAAGGAAGTCGCACAGCATTTCCGAATGGGGCTGAGCAATATGCGCGGCTACTACCACCAGAGCGAGGCCGGGTCTCACACCCTCCAGGGGATATTTGCCTGCGACGTGGGGCCGGACGGGCGCCTCCTCCGCGGGTACTGGCAGCTCGCCTACGACGGCGCTGACTACCTCGCCCTGAACGAGGACATGCGCTCCTGGACCGCGGCCGACAAGGCGGCTCAGATCACCCGGCGCAAGTGGGAGGCGGCCGGTGGGGCGGAGCGCCTGAGAGGCTACTTTGAGGGCACCTGCGTGGAGTCGCTCCGCAAATACCTGGAGATCGGGAAGGAGACGCTGCTGCGCGCAGACCCTCCAAAGGCACACGTGACTCACCACCCCACCTCTGACCGTGAGGTCACCCTGAAGTGCTGGGCGCTGGGCTTCTACCCTGCGGAGATCACCCTGACCTGGCAGCGTGATGGGGAGGACCTGACCCAGGACACGGAGCTCCTGGAGACCAGGCCTGCGGGGGACGGGACCTTCCAGAAGTGGGCAGCTGTGGCGGTGCCTCCTGGAGAGGAGCAGAGATACACGTGCCATGTGCAGCACGAGGGGCTGCCCGAGCCCCTGACCCTGAGATGGGAGTCGCCTCCTCAGGCCGCCATCCCTGTGGGCATCATTGCTGTGCTGGTCCTTGGAGCTGCGGTCACTGGGGCTGTGACAGCTGGAGCTGTGCTATGGAGGAGGAGGCGTCACAGAGGTGGAAAAAGAAGGCGCTATGCTCAGGCTGCAAGCAGTGACAGTGCCCAGGGCTTTGATGTGTCTCTCACAGCTTCTAAA

>Hiar_G28_pr_NW017733293_1408_13585

ATGCGGTTAATGGGCTCCGGGACCCTCCTCCTGCTGCTCTCGGGGGCCCTGGGCCTGACGGGGACCCGGGCGGGCTCCCACTCCCTGAGGTATTTCGAGACATCCTGGTCCCGGCCCGGCGGCGGGGAGCCCCGGTTCATCGCCGTCGGCTACGTGGACGACACGCAGTTCGTGCGGTTCGACAGCGACGCGGCGAGTCCGAGGATGGAGCCGCGGGCGGCGTGGATGGAGGGGCCGTGGGTGGAGCAGGCGCTGCCGGGGTACTGGGAGCAGCAGACGCAGCGCTGCAAGGACAACGCACAGATTCACCGAGGGAACCTGCAGACCGCGCTCCGCTACTACAACCAGAGCGAGGCCGGGTCTCACACTGTACAGGGAATGTATGGCTGCGACGTGGGGCCGGACGGGCGCCTCCTCCGCGGGTACAGACAGGACGCCTACGACGGCGCTGACTACCTCGCCCTGAACGAGGACCTTTCCTCCTGGACCGCGGCCGACACGGCGGCTCAGATCACCCGGCGCAAGTGGGAGGAGGCCGGTGCGGCGGAGCATTACAGGGGCTACTTGGAGGGCAGGTGCCTGGAGTGGCTCCGCAAATACCTGGAGAAGGGGAAGGAGACGCTGCTGCGAGCAGACCCTCCAAAGGCACACGTGACCCATCACCCCAGCTCTGACCGTGAGGTCACCCTGAGGTGCTGGGCGCTGGGCTTCTACCCTGCGGAGATCACCCTGACCTGGCAGTGTGATGGGGAGGACATGACCCAGGACACGGATCTCGTGGAGACCAGGCCTGCGGGGGACGGGACCTTCCAGAAGTGGGCAGCTGTGGTGGTGCCTCCTGGAGAGGAGCAGAGATACACGTGCCATGTACAGCACGAGGGGCTGCCAGAGCCCCTGACCCTGAGATGGGAGCCGCCTCCTCAGGCCACCAACTCCGCTGGCACGATGAGAGGCATCACTGTAGGGGTGCTCTGTGTCGCTGGAACTGTGCTGTGGATGAAGAAGCGCTTAGGTGGAAAAGGAGGGAGCTACGCTCAGGCTGCAAGCGGTGACAGTGCCCAGGGCTCTGATGTGTCTCTCGCTGGTTCTAAA

>Hiar_G26_pr_NW017733582_82_1286

ATGCGCATCATGGGTCCCGGGACCCTCCTCCTGCTGCTCTCGGGGGCCCTGGCCCTGACGGGAACCCGGGCGGGCTCCCACTCCCTGAGGTATTTCGCCACCTCCTGGTCCCGGCCCGGCCGCGGGAAGCCCCGGTTCATCGAAGTCGGCTACGTGGACGGCACGCAGTTCGTGCGGTTCGACAGCGACGCGGCGACCCCGAGGATGGAGCCGCGGGCGGCGTGGATGGAGGGGCCGTGGGTGGAGCAGGAGTACCCGGGGTACTGGGACCAGGAGACGCAGCGCTGCAAGGGAAACGCACAGAATAACCGAGTGAGCCTGAACAACCTGCGCGGCCACTACAACCAAAGCGAGGCCGAAGGGTCTCACACCCTCCAGAGGATGTACGGCTGCGACGTGGGGCCGGACGGGCGCCTCCTCCGCGGGTACAGGCAGGACGCCTACGACGGCGCTGACTACCTCGCCCTGAACGAGGACCTTTCCTCCTGGACCGCGGCCGACACGGCGGCTCAGATCACCCGGCGCAAGTTGGAGGCGGCCGGTATGGCGGAGCAATGGAGGGGCTACTTTGAGGGCAGGTGCCTGGAGTCGCTCCGCAAATACCTGGAGAAGGGGAAGGAGACGCTGCTGCGCGCA

>Hiar_G30_pr_NW017733048_39424_40126

ATGGGCCACGCGACCCTCCTCCTGCTGCTCTCGGGGGCCCTGGGCCTGACGGGGACCCGGGCGGGCTCCCACTCCCTGAGGTATTTCTTCACCTCCTGGTCCCGGCCCGGCGGCGGGGAGCCCCGGTTCATCGCCGTCGGCTACGTGGACGACACGCAGTTCGTGCGGTTCCACAGCGACGCGGCGAGTCCGAGGGCGGAGCCGCGGGCGGCGTGGATGGAGCACATAGAGCAAGTGGACCCGGAGTACTGGGACCGGAACACGCTGATCTACAAGGACAACGCACAGAGTCACCGAGTGTGGCTGAACACCCTCCGCGGCTACTACAACCAGAGCGAGGCCGGT

>Hiar_G83_ps_NW017731574_1369941_1370869

AACTCCCTGAGGTATTTCTACACCTCCTGGTCCTGTGAGACCCGGTTCATCTCCCTCGGCTACGTGGATGACACGGAGTTCGTGCGGTTCGACAGCAACGCCGCGAGTCCGAGGATGGAGCGGACGCCGTGGATGGAGGGGCCGTGGGTGGAGCAGGCGCTGCCGGGGTACTGGGAACAACAGACGCAGGTCAGCAAGGGCCATGCACAGAATGAACCGGCGCTCCACTACTGCCACCAGAGCGAGGCCGGGTTTCACACCTACCAGGTGATGTGCAGCTGCGACCTGGGGCCGGTCGGGCGCCTCCTCCGCGCTTCTGAGCAGTATACCTACGATGGCGCTGACTACACCGCCCTGAACGAGGACCTGCGCTCCTGGACTGCGGCCGACAGG!!GCGCAAGTGGGAGGTGGCCCGTGCGGCGGAGCACCTGAGGGGCTACCGTAAGAGCCTGTGTGTGGAGTGGCTCCGCAAATACCTGGAGAAGCGGAAGGAAACGCTGCAGCGCGCAGATCCTCCAAAGCCATACGTGATCCACCACCCCACCTCTGACCGTGACGTCTCCCTGAGGTGCTGGGCGCTGGGCTTCTACCCTGTGGAGATCACTCTGACCTGGCAGCGTGATGGGGAGGACCTGACCCAGGACGTGAAGCTCTTGGAGACCAGGCCTGCGGGGGACGGGACCTTCCAGAAGTGGGCAGCTGTGGTGGTGCCTCCTGGAGAGGAGCACAGATACACGTGCCATGTGCAGCACAAGGGGCTGCCCGAGCCCCTGACCCTGAGAAGGAAGCTGCCTCCTCAGGTCACCGTTCCTGTGGGCATCATTGCTGTGCTGGTCCTCCTTGGAGCTGCGTTCACTGGGGCTGTGGTGGCCGGAGCTGTGCTGTGGAGGAGGAGGTGCTCAGGCTGCAAG!CA

>Hiar_G111_ps_NW017731825_29162_32145

ATGGGCCTCGGGACCCTCCTCCTGCTGCTCTCGGGGGCCCTGAGCCTGACGGGGACCCGGGCGGGTTCCCACTCCCTGAGGTATTTCGACACCTCCTGGTCCCGGCCCGGCCGTGGGGAGCCCCGGTACATCGCCGTCGGCTACGTGGACGGCACGCAGTTCGTGCGGTTCGACAGCGACGCCGCGAGTCCGAGGATGGAGCCGCAGGCGGCGTGGATGGAGGGGCCGTGGGTGGAGCAGGCGCTGCCGGGGTACTGGGAGCGGGAGACGCAGNAGACCGTCTCAGAAACACCTGATCAGCGGTTCCCTTTGGCCCCGGCAGCTGCCCGGGAACCTGGGGGACTTTCTCTCTCAGGCCTTGTTCTCTGCCCCACACACAGTGTGACTGAGGTCAGGACCAGGAATCCCTTTCGCCTCCATCAGAGACCTGTCTCTGCCCCTGGGCTGTCTCACCCTGGTTCTAGGACTTTCTATAGAATAGAGATGATCCCGGATGCCTGTGTCCAGTGTGAGGTGTGGGTTTTGTGCTCCCTTCCCCCACCCCAGTTTCCTGACCATTCTCGGGATGACCCTCCAAAGGCACACGTGACCCACCACCCCACCTCTGACCATCAGGTCACCCTGAGGTGCTGGGC!TTCTACCCTGCGGAGATCACCCTGACCTGGCAGCATGATGGGGAGGATCTGACCCAGGACACGGAGCTCGTGGAGACCAGGCCTACGGGGGACGGAACCTTCCAGAAGTGGGCAGCTGTGGCGGTGGTGCCTCCTGGAGAGGAGCGGAGATACACGTGCCATGTGCAGCACGAGGGGCTGCCCGAGCCCCTGACCCTGAGATGGGAGCCACCTCCTCAGGTCACCATCTCCATTGGCATGGTGATGGGCATCATTGTTGGGGTGATGGTCCTCCTTGGAGCTGTGGTGGCTGGAGCTGTGCTGTGGAGGGGGAGGCACTCAGGTGGAAAAGGAGGGAGCTACGCTCAGGCTGCAAGTGGTGACAGTGCCCAGGGCTCTGATGTGTGTCTCACAGCTTCTAAAGGT

>Hiar_G15_ps_NW017733636_50297_53300

ATGAGCGCCGGGGCCCTCGTCCTGCTGCTCTCGGGGGCCCTGGGCCTGACGGGGACCCGGGCGCGCTCCCACTCCTTGAGGTATCACCGCACCTCCTGGTCCCGGCCCGGCGGCGGGGAGCCCCGGTACATCTCCGTCGGCTACGTGGACGACACGCAGTTCGTGCGGTTTGACAGCGACGCAGCGAATCCGAGGATGGAGCCGCGGGCGGCGTGGATGGAGGGGCCGTGGGTGGAGCAGGCGCTGCCGGGGTACTGGGACGAGCAGACGCAGATCTGCAAGAGCACCGCACAGATTAACCGAGGGAGCCTGAACAACCTGCGCGGCTACTACAACCAGAGCGAGGCCGGGTCTCACACCTACCAGAGGATCACTGGCTGCGATGTGGGGCCGGACGGGCGCCTCCTCCGCGGGTACAGGCAGCTCGCCTACGACGGCGCTGACTACCTCGCCCTGAACGAGGACCTTTCCTCCTGGACCGCGGCCGACACGGCGGCTCAGATCACCCGGCGCAAGTGGGAGGCGGCCGGTGAGGCGGAGCGCTGGAAGGGCTACTTTGAGGGCAGGTGCCTGGAGTGGCTCCGCAGATACCTGGAGAAGGGGAAGGAGACGCTGCTGCGCGCAGACCCTCCAAAGACACACGTAACCCACCACCCCACCTCTGACCATGAGGTCACCCTGAGGTGCTGGGCGCTGGGCTTCTACCCTGCGGAGATCACCCTGACCTGGCAGCGTGATGGGGAGGACCTGACCCAGGACACAGAGCTCGTGGAGACCAGGCCTGCGGGGGACGGGACCTTCCAGAAGTGGGCAGCTGTGGCGGTGCCTCCTGGAGAGGAGCAGAGATACACGTGCCATGTGCAGCACGAGGGGCTGCCCGAGCCCCTGACCCTGAGATGGGAGCCGCCATCTCAGGTCGCCATCTCCATTGGCATAATGATGGGCATCATTGTTGGGGCGCTGGTCTTGCTCGGAGCTGCAGTCGCTGGAGCTGTGTTGTGGAGGAGGAGGCACTCAGGTGGAAAAGGAGGAAGCTACGCTCAGGCTGCAAGTGGTGACAGTGCCCAAGGG!!CTCTGATGTGTCTCTCACAGCTTCTAAAGGT

>Hiar_G53_ps_NW017733408_11151_11946

ATGTGCGTCGTGGGCCTCGGGACCCTCCTCCTGCTGCTGTCGGGGGCCCTGGGCCTGACGGGGACCCGGGCGGGCTCCCACTCCATGAGGTATTTCCACACCTCCTGGTCCCGGCCCGGCGGCGGGGAGCCCCGGTACATCTCCGTCGGCTACGTGGACGACACGCAGTTCGTGCGGTTCGACAGCGACGCGGTGAGTCCGAGGATGGAGCCGCGGGCCGCGTGGATGGAGCACATAGAGCAAGTGGACCCGGCGTACTTGGACCGGAACACGCAG!!ATCAGCCAAGGGCTGGGCACAGAGTTACCGAGTGGGACTGAACACTCTGCGCGGCTACTACAACCAG

>Hiar_G27_ps_NW017731733_1305461_1306548

ATGCGGATCATGGGCCCCGGAACCCTCATCCTGCTGCTCTCGGGGGCCCAGGCCCTGACGGGGACCCGGGCGGGCTCCCACTCTCTTAAGTATTTCTCCATCTCCTGGTCCCAGCCCGGCCGCGGGGAGCCCCGGTTCATCTCCGTCGGCTACGTGGACGACACGCAGTTCGTTCGGTTCGACAGCGACGCAGAGAGTCCGAGGGAGGAGCCACGGGCGGCGTGGATGGAGGGGCCGTGGGTGGAGCAGGCGCTGCCAGGGTACTGGGAGCGGCAGACGCAGACCTGCAAGGTCAGCGCACAGAATGACCGAGTGAGCCTGAACAGCCTGCGCGGCTACTACAACCAGAGCGAGGCGGGGTCTCACACATTTCAGTGGATGTCTGGCTGCGACGTGGGGCCGGACGGGCGCCTCCTCCGCGGGTACATGCAGTACACCTACGACGGCACCGACTACATCTCCCTGAACCAGGACATGCACTCCTGGGCCGCAGCCGCCACTGCGGCTCAGATCACC!AGCACAAGTGGGAGGCGGCGGGTGAGGCGGAGATCCTGAGGGGCTACTTTGAGAGCACGTGCCTGGAGTGGCTCCGCAAATACCTGGAGAAGGGGAAGGAGACGCTGCAGCGCGCAGACCCTCCAAAGACACACGTGACCCACCACCCCACCTCTGACCGTGAGGTCACCCTGAGGTGCTGGGCGCTGGGCTTCTACCCTGCGGAGATCACCCTGACCTGGCAGCGTGATGGGGAGGACCTGACCCAGGACACGGAGCTCGTGGAGACCAGGCCTGCGGGGGACGGGACCTTCCAGAAGTGGGCAGCTGTGGTGGTGCCTCCTGGAGAGGAGCAGAGATACACGTGCCATGTGCAGCACGAAGGGCTGCCCGAGCCCCTGACCCTGAGATGGGAGCCACCTCCTCAGGCCACCATCCCAGTGGGCATCATTGCTGTGCTGGCCCTTGGAGCTGTGGTGGCTGGAGCTGTGCTGTGGAGGAGGAGGCGCTCAGGTAGAAAAGGAGGGAGCAATGCTCAGGCTGCAAGCGGTGACAGTGCCCAGGACTCTGATGCATCTCTCACTGCTTCTAGC

1. **Natal longfingered bat *Miniopterus natalensis***

>Mina_G28_pr_NW015504258_24178382420003

ATGAGGCCCCCAACCCTCCTCTTGCTGCTCTGGCCGACCCTGGTTAGGACCCAGCCCGGGGCGGGCTCCCACTCCCTGGTCTACTTCCACACCGCCGTGTCCCGGCCGGGCCGCGGGGAGCCCCGGTACCTCGAAGTCGGCTACGTGGACGACACCCAGTTCGAGCGGTTCGACAGCGACTCCAGGAGCCAGAGGGCGGAGCCGCGGGCGGGGTGGATGGAGCTGGTGGAGCTGGAGGAGCCCGGCTACTGGGAGCTGAGCACCCGGAACGCGCGGGCCTCGGCGCGGGCGTCCCGCGTGAACCTGCAGACGTTCATCGACTACTACAACCAGAGCCGCGACGGGTCTCATACCTTCCAGGCCATGTGGGGCTGTGAAATGGGACCGGATGGGCGCCTCCTTCTTGGCTATTTCCAACAAGCCTACGACGGCACCGACTACATCGCGCTGAACGATGACCTGCGCTCCTGGACCTCCGCCGACCGGGCGGCGCAGATCACCCAGCGCAAGTGGGAGGCGGCGGGCGAGGCTGATGCCATCAGGAGCCGACTGCAGGGCTTGTGCCTGCAGATGCTCCAGAGATTCCTGGAAATGGGGAAGGAGACTCTGCAGCGCGCGGACCCGCCAAAGACACATGTGACCCACCACCCCATCTCTGACAATAAGGTCATCCTGAAGTGCTGGGCCCTCGGCTTCTACCCTGCGGAGATCACCTTGACCTGGCTGCGTGATGGGGAGGACCAGTCCCAGGACATGGAGGTTGTGGAGACCAGGCCTGCAGGGGATGGGACCTTCCAGAAGTGGGCGGCTGTGGCAGTGCCCGCTGGAGAGGAGCAGAGTTACACCTGCCATGTGCGGCACGAGGGGCTGCCCGAGCCTGTGACCCTGAGTTGGGAGCCAGCTCCTCAGGCCACCATCCCCAATGTGGTCATCATTGTTGTCCTGTTCTTCCTTGGGGCTGTGATCTGTGGAACTGTGGCTGCTGCAGCTGTGATGTTCAGGAGGAAGCGCTCAGGTAGGCAAGGA

>Mina_G15_NW015505110_1895024570

ATGGGGTCCCCGCCCCTCCTCCTGCTGCTCGCGGGGGCCCTGGTCCTGACCCGCACCCGGGCCGGCCCCCACTCCCTGAGATATTTCCTCACCACCTTGTCCCGGCCCGGCCGCGGGGAGCCCCGGTACCTGGAAGTCGGCTACGTGGACGACACGCAGTTCGTGCGGTTCGACAGCGACGCCGCGAATCCGAGGATGGAGCCGCGGGCGCCGTGGATGGAGCAGCCGTGGGTGGAGCAGGAGGACGCGGGGTATTGGGACCGGAACACGCGGATCAGCAAGGAAAATGCAGAGACTGACCGAGTGAACCTGAAGACCGCCCTCGGCTACTACAACCAGAGCGAGGACGGTGAGCCCCGGGCTACTACAACCAGAGCGAGGACGGTGAGCCCCGCGACCCGGCCCCGTCACCACCCCCATCCCCATGGACCGGCCGGGTCGCCCCGAGTGTCGGGATCCGAGGAGGACCTGCGCTCCTGGACCGCGGCCGACACGGCGGCTCAGATCACCCGGAGCAAGTGGGAGGCGGCCGGTGAGGCGGAGAGAGTCAGGAACTACCTGGAGGGCCGGTGCGTGGACTCGCTGCGCCGTTACCTGGAGAAGGGGAAGGAGACCCTGCTGCGCACAGACCCTCCAAAGACACACGTGACCCGCCACCCCATCTCTGAGCATGAGGTCACCCTGAGGTGCTGGGCCCTGGGCTTCTACCCTGCGGACATCAGCCTGACCTGGCAGCGAGACGGGCAGGACCAGACCCAGGACATGGAGTTTGTGGAGACCAGGCCCTCGGGGGACGGGACCTTCCAGAAGTGGGCGTCCCTGGGGGTGCCCCCTGGAGAGGAGCAGAGATACACCTGTGTTGTGCAGCACGAGGGGCTGCTGGGGCCCCAGAGCCTGAGATGGGAGCCTCCCTCTCAGACCCCCTTCATCATCATGGGCGCTGTTGTTGGCCTGGTTGTCCTCGGAGCTGTGGTGGCTGGAGCTGTGATGTGGGCGAGGAGGCGCTCAGGTGGAAAAGGAGGGAGCTACGCTCAGGCTGTCAGT

>Mina_G37_pr_NW015505110_54247965

GGCCCCCACTCCCTGAGATATTTCCTCACCACCGTGTCCCGGCCCGGCCGCGGGGAGCCCCGGTACCTGGAAGTCGGTTACGTGGACGACACGGAGTTCGTGCGGTTCGACAGCGACGCCGCGAATCCGAGGATGGAGCCGCGGTCGCCGTGGATGGAGCAGGAGATGCCGGGGTATTGGGACCGTGAGACGCGGAGGAACAAGGAAAATGCACAGATTTACAGAGTGAACCTGAAGACCCTGCGCGGCTACTACAACCAGAGCGAGGACGGGTCTCACACCATCCAGAGGATGTACGGCTGTGACCTGGGACCGGACGGGCGCCTCCTCCGCGGGTATGATCAGCACGCCTACGACGGCGCCGACTACCTCGCCCTGAACGAGGACCTGCGCTCCTGGACCGCGGCCGACACCGCGGCTCAGATCTCCCGGCGCAAGTGGGAGGCGGACGGTTATGCGGAGAGACAGAGGAACTACCTGGAGGGCCGGTGCGTGGAGTGGCTGCGCCGTTACCTGGAGAAGGGGAAGGAGACCCTGCTGCGCACAGACCCTCCAAAGACACACGTGACCCGCCACCCCATCTCTGAGCATGAGGTCACCCTGAGGTGCTGGGCCCTGGGCTTCTACCCTGCGGACATCAGCCTGACCTGGCAGCGAGACGGGCAGGACCAGACCCAGGACATGGAGTTTGTGGAGACCAGGCCCTCGGGGGACGGGACCTTCCAGAAGTGGGCGTCCCTGGGGGTGCCCCCTGGAGAGGAGCAGAGATACACCTGTGTTGTGCAGCACGAGGGGCTGCTGGGGCCCCAGAGCCTGAGATGGGAGCCTCCCTCTCAGACCCCCTTCATCATCATGGGCGCTGTTGTTGGCCTGGTTGTCCTCGGAGCTGTGGTGGCTGGAGCTGTGGTGGCTGGAGCTGTGATGTGGGCGAGGAGGCGCTCAGGTGGGAGAGGAGGGAGCTACGCTCAGGCTGCCAGCAGTGACAGCTCCCAGGGCTCTGATGTGTCTCTCTCGGCTTCTAAA

>Mina_G34_pr_NW015504604_929552932990

GCAGGGCCCCACTCCCTGAGATATTTCCACACCGCCGTGTCCCGCCCCGGCCGCCGGGAGGCCCTCTACATCTCCGTCGGCTACGTGGACGACACGCAGTTCCTGCGGTTCGACAGCGACGCCGCGAGCCCCAGGGTGGAGCCGCGGGCGCCGTGGATGGAGCAGGAAGCGCAGCCCTTCTGGGAAGCTCAGACCGAGATCGCCCAGGTCCACGCGCAGATTTCCCGCGTGAACCTGCAGACGGCCCTCGGCTACTACAACCAGAGCGAGTCCGGGTCTCACACCTTCCAGTGGACGTCCGGCTGCGACGTGGGGCCCGACGGGCGCTTCCTCCGCGGGTACGAGCAGTTCGCCTACGACGGCGCCGACTACATCGCGCTGAGCGAGGACCTGCGCTCCTGGACCGCGGCGGCCCCGGCGGCCCAGCTCACCCGGCGCAAGTGGGAGGCGGCGGGGCTGGCCGAGCGGTACCGGGCGTACCTGGAGAGGGAGTGCGTGGAGCTGCTGCGCCGCTACCTGGAGAACGGGAGGGAGACGCTGCAGCGCGCAGATCCCCCAGAGACACATGTGACCCATCACCCCATCTCTGACCGTGAGGTCAGCCTGAGGTGCTGGGCCCTGGGCTTCTACCCTGCGGAGATCACCCTCACCTGGCACCGCGATGGGGAGGAGCAGACCCAGGACACGGAGCTTGTGGAGACCAGGCCTGCGGGGGACGGGACTTTCCAGAAGTGGGCGGCCGTGGTGGTGCCCCCTGGAGAGGAGCAGAGATACACGTGCCATGTGCAGCACGAGGGGCTGCCCGAGCCCCGCACACTGAGATGGGAGCCACCTCTTCAGCCCACCTTCATTGCTGTCCTTGTTTTTGGAGCTGTGGTCACTGGAGCTGTGGCAGCTGGAACTCTGGCAGCTGGAGCTGTGATGTGGGGGAAGAAGGGTACAGGTGCAAAAGGACGGAGCTACACTCAGGCTGCCAGCAGTGATGTGTCTCTCAGGGCTCCTGAAGCCCTGACAGAAGCTGTG

>Mina_G2_pr_NW015504106_201164

TCAGACCCTCCAAAGACACACGTGACCCGCCACCCCATCTCTGAGCATGAGGTCACCCTGAGGTGCTGGGCCCTGGGCTTCTACCCTGCGGACATCAGCCTGACCTGGCAGCGAGACGGGCAGGACCAGACCCAGGACATGGAGTTTGTGGAGACCAGGCCCTCGGGGGACGGGACCTTCCAGAAGTGGGCGTCCCTGGGGGTGCCCCCTGGAGAGGAGCAGAGATACACCTGTGTTGTGCAGCACGAGGGGCTGCTGGAGCCCCAGAGCCTGAGATGGGAGCCTCCCTCTCAGACCCCCTTCATCGTCATGGGCGCTGTTGTTGGCCTGGTTGTCCTCGGAGCTGTGGTGGCTGGAGCTGTGATGTGGGAGAGGAGGCGCTCAGGTGGGAAAGGAGGGAGCTACGCTCAGGCTGCCAACAGTGACAGTGCCCAGGGCTCTGATGTGTCTCTCTCGGCT

>Mina_G23_pr_NW015505175_751214

TCAGACCCTCCAAAGACACACGTGACCCGCCACCCCATCTCTGAGCATGAGGTCACCCTGAGGTGCTGGGCCCTGGGCTTCTACCCTGCGGACATCAGCCTGACCTGGCAGCGAGACGGGCAGGACCAGACCCAGGATATGGAGTTTGTGGAGACCAGGCCCGCGGGGGACGGGACCTTCCAGAAGTGGGCGTCCCTGGGGGTGCCCCCTGGAGAGGAGCAGAGATACACCTGTGTTGTGCAGCACGAGGGGCTGCCGGGGCCCCGGGCCCTGAGATGGGAGCCTCCCTCTCAGACCCCCTTCATCATCATGGGCACTGTTGTTGGCCTGGTTGTCCTCGGAGCTGTGGTGGCTGGAGCTGTGATGTGGGCGAGGAGGCGCTCAGCAGGTGGGAAAGGAGGGAGCTACGCTCAGGCTGCCAGCAGTGACAGCGCCCAGGGCTCTGATGTGTGTCTCTCGGCT

>Mina_G50_pr_NW015504619_914984916128

TCAGACCCTCCAAAGACACACGTGACCCGCCACCCCATCTCTGAGCATGAGGTCACCCTGAGGTGCTGGGCCCTGGGCTTCTACCCTGCGGACATCAGCCTGACCTGGCAGCGAGACGGGCAGGACCAGACCCAGGACATGGAGTTTGTGGAGACCAGGCCCTCGGGGGACGGGACCTTCCAGAAGTGGGCGGCCCTGGGGGTGCCCCCTGGAGAGGAGCAGAGATACACCTGTGTTGTGCAGCACGAGGGGCTGCTGGAGCCCCAGAGCCTGAGATGGGAGCCTCCCTCTCAGACCCCCTTCATCATCATGGGCGCTGTTGTTGGCCTGGTTGTCCTCGGAGCTGTGGTGGCTGGAGCTGTGATGTGGGCGAGGAGGCGCTCAGCAGGTGGGAAAGGAGGGAGCTACGCTCAGGCTGCCAGCAGTGACAGCGCCCAGGGCTCTGATGTGTCTCTCTCGNCG

>Mina_G9_pr_NW015504594_36155814

GACTACATCTCCCTGAACGAGGACCTGCGCTCCTGGACCGCGGCCGACACGGCGGCTCAGATCTCCCGGCGCAAGTACGAGTCTGTGGACGAGGCGGAGGTCCGGAGGAACTACCTGGGGACGGAGTGCATGGAGTGGCTCCGCAGATACCTGGACGAAGGGAAGGCGACGCTGCAGCGCGCAGATCCCCCAGAGACACATGTGACCCATCACCCCATCTCTGACCGTGAGGTCAGCCTGAGGTGCTGGGCCCTGGGCTTCTACCCTGCGGAGATCACCCTCACCTGGCACCGCGATGGGGAGGAGCAGACCCAGGACACGGAGCTTGTGGAGACCAGGCCTGCGGGGGACGGGACTTTCCAGAAGTGGGCGGCCGTGGTGGTGCCCCCTGGAGAGGAGCAGAGATACACGTGCCATGTGCAGCACGAGGGGCTGCCCGAGCCCCGCACACTGAGATGGGAGCCACCTCTTCAGCCCACCTTCATTGCTGTCCTTGTTTTTGGAGCTGTGGTCACTGGAGCTGTGGCAGCTGGAACTCTGGCAGCTGGAGCTGTGATGTGGGGGAAGAAGGGTACAGCTGCAAAAGGATGGAGCTACGCTCAGGCTGCCAGCAGTGACAGTGTTGAGGGCTGTGATGTGTCTCTCATGGCTTCTGAA

>Mina_G12_ps_NW015504692_29755022976126

ATCTCCCTGAATGAGGACCTGAGCTCCTGGACCGCAGTGGACACTGAGGCTCAGATCACGCTGCACAGGTGCCTGGGCACTGTAGGAACTACCCCGACGACATGTGCGTGCAGTGGCTCCGCAGATACCTGGCAAAAGGGAAGGAAGGAGATGCTGCAGCCCCCAGACCCTCCAAAGACACATACGACCCACCACCCCATCTCTGACCATGAGGTTACCCTGAGGTGTTGG!CCCTGGTCTTCTACTCTGCAGAGATCACCCTGACCTGGCAGCGTGAC!!AGGGAAGCCT!AGACCCAGGACATGGAGCTTGTGACCAGGCCTGCAGGGGAGGGGCCCTTCCAGAGGTGGGCAGCCCTGGTGGTGCCTTCTGGAGAGGAGCAACCCAAGGCTCTGACCCTGAGATGGGAACTGCCTCCTCAGGCCACCTTCCCCACTGTGGGCATCCTGGCTGTGCTAGTCATCCTTGGAGCTGTGGTCACTGGAGCTGTGATGAGGAGAAAGCACTCAGGTGGAAAAGGAGGGAGCTACGCTCAGGCTGTCAGCAGTGACAGTGCCCAGGGCTCTGATGTCCCTCTCAGGGCTTCTAAAGCT

1. **Chinese horseshoe bat *Rhinolophus sinicus***

>Rhsi_G101_NW017739099_6196_9794

ATGCGAGTCAAGGGGCTGGGAACCCTCCTCCTGCTGCTCTCGGGGGCTCTGGCCCTGATGAGGACCCGGGCGGGCAACCACGCCATGAGGTATTTCATCACCACCTGGTCCCGGCCCGGCCACGGGGAGCCCCGGTACATCGTCGTCGGCTACGTGGACGACACGCAGTTCCTGCGTTTCGACAGCGACGGCACGAGTCCTAAGGCGGAGCCGCGGGCGGCGTGGATGGAGGGGCCGTGGCTGGAGCAGGTGGACCCGGGGTACTGGGACCGGGAGACAGGACGCGCCAAATTCTACCAACAGATTTTGCGAGCGGACCTGCAGAGCGCGCTGGAGAACTACAACCAGAGCGAGGCCGGTGAGCACTTTGGGGTGAAGGGGGCGGGGCTGCCCACAGGACGGAGGCCTGATGCCAGCTTTTCTGAATCATTCACCCTCCACCCAGGTCAGGACTCTGTTCTTTTCCTTAGAGACCTGGAGCCTCCAATTCTAGGTTCTCACCATGATTCTAGAACTTTCCAAGGAATCAGATTATCCCAGAATATGGATCCTACACTGAAGATGGTCATGAGCGCAGCTTCAATGTCCCATGGAGATAACCCAAAGTGTGGATTTTCTGATTCTGCCTCCTCAGACCCTCCAAAGACACACGTGACCCACCACCCCATCTCTGACCACGATGTCACCCTGAGGTGCTGGGCCCTGGGCTTCTACCCTGCGGAGATCACCCTGACCTGGCAGCGTGATGGGGAGGACCTGACCCAGGACACAGAGCTCGTGGAGACCAGGCCTGCAGGAGATGGGACTTTCCAGAAGTGGGTGTCTGTGGGGGTGCCTTCTGGAGAGGAGCAGAGATACACGTGTCATGTACAGCACAAGGGGCTGCCCGAGCCCCTCACCCTAAGATGGGAACCACCTCCTCAGCCCACCATTCCTATTATGGGCATCATTGTTGGTCTGGTTCTCTTTGTGGTCACTGGAGCTGTGGTGGCTGGAGCTGTGCTGTGGAGGAAGAAGCACTCAGGTGGAAAAGGAGGGACCTATGCTGAGGCTGCAAGCAGTGACAGTGCCCAGGGCTCTGATGTTTCTCTCATGGCTTTAAAGGTG

>Rhsi_G106_pr_NW017740154_16482_17110

ATGGGCCCCGGAACCCTCCTGCTGCTGCTGTCGGGGGCCCTGGGCCTGACGGGGACTCGGGCGGGTGAGTGCGGGGTCGGGAGGGAACGGTCTCCACCGGTGGGGAAAGCGAGGGCACCGCCCGATGGGGACGCTGAACCCCGGACGCAGTTCCTGCGGTTCTACAGCGACGCGGCGAGTCCGAGGGTGGAGCCGCGGGCACCATGGATGGAGGGGCCGTGGATGGAGCAGAAGGAGCCGGGTTACTGGGACCGTGAGACACGGATTGCCAAGAGCCACGCACAATTTGCTCGAGCGGAGCTGCAGACCGCGCTCCAGAACTATAACCACAGTGATGCCGGT

>Rhsi_G33_pr_NW017741421_3357_4131

GGATCCCACTCCATGAGGTATTTCGCCACCTCTTGGTCCCGGCCCGGCCGCGAGGATCCCCGCTATATCGCCGTCGGCTACGTGGACGACACGCAGTTCGTGCGGTTCGACAGCGACGCAGCGAGTCCGAGGATGGAGCCGCGGGCGGCGTGGATGGAGGGGCCGTGGGTAAACCAGGTGGTGCAGGGGTACTGGGACGACCAGACGCAGGTCTGCAAGTACTCCGCACAAGTTGACCGTGTGGAGTTGAACAACATCCGCGGCTACTACAACCAGAGCGAGGCCGGGTCTCACACTCTCCAGTGGCTGTCCGGCTGCGACGTGGGGCCGGACGGGCGCCTCCTCCGCGGGTACAGGCAGGTCGCCTACGACGGCACCGACTACCTCGCCCTGAACGAGGACCACCGCTCCTGGACCGCGGCGGACACGGCGGCTCAGATCACCCGGCGCAAGTGGGAGGCGGCCGGTGTGGCGGAGTACCTGAGGGGCTACTATGAGGGCATGTGCAGGGAGTCGCTCGGCAAATAC

>Rhsi_G92_pr_NW017741430_2817_4194

ATGCGCATCAAGGGCCCCGGAACCGGAACCCTCCTCCTGCTGCTCTCGGGGGCTTTGGTTCTGACGGGGACCTGGGCGGGATCCCACTCCCTGACATATTTCGACACCACCTGGTCCCGGCCCGGCAGCGGGGAGCCCCGGTACATCACCGTCGGGTACGTGGACGGCACGCAGTTCGTGCGGTTCGACAGCGACGCAGCGAGTCCCAGGATGGAGCCGCGGGCTGCGTGGATGGAGGGACCGTGGGTAAACCAGGTGGTGCAGGGGTACTGGAACCAGCAGACGCAGGTCTGCAAGAGCGCCGCACGTGCTACCCGAGTGGAGCTGAACAACTTGCGCCGCTACTACAACCAGAGCGAGGCCGGGTCTCACACCATTCAGAGGATGTTCGGCTGCGACGTGGGGCCGGACGGGCGCCTCCTCCGCGGGTACAGGCAGGACGCCTACGACGGCGCCGACTACATCGCCCTGAACGAGGACCTGCGCTCCTGGACCGCGGCTGACTCGGCGGCTCAGATCTCCCGGCGCAAGTGGGAGGCGGCCGGTGTGGCGGAGCGATGGAGGGGCTACTTGGAGGGCACGTGCGTGGAGTCGCTCGGCAAATACCTGGAGAAGGGGAAGGAGACGCTGCTGCGC

>Rhsi_G60_pr_NW017739684_440534_442875

TCAGGGTCTCACACCTACCAGAGGATGTGTGGCTGCGACGTGGGGCCGGACGGGCGCCTCCTCCGAGGCTACAGGCAAGACGCCTACGACGGCGCCGACTACGTCGCCCTGAACGAGGACATGCGCTCCTGGACCGCGGCGGACAAGGCTGCTCAGATCACCCAGCGGAAGTGGGAGGCGGCCGGTGAGGCGGAGCGCCTGAGGGGCTACTATGAGGGCACGTGCGTGCAGTGGCTCCTCAGACACCTGGAGAACGGGAAGGAGACACTGCTGCGCGCAGACCCTCCAAAGACACATGTAACCCATCACCCCATCTCTGACCATGATGTCACTTTGAAGTGCTGGGCACTGGGCTTCTACCCTGCGGAGATCACCATGATCTGGCAGCGTGATGGGGAGGACCTGACCCAGGACACGGAGCTCGTGGAAACCAGGCCTGCGGGGGATGGGACCTTCCAAAAGTGGGCAGCTGTGGGGGTGCCTTCTGGAGAGGAGCAGAGATACACGTGTCATGTGCAGCACAAGGGGCTGCCCAAGCCCCTCACCCTGAGATGGGAACCACCTCCTCAGCCCACCATTTCTATTATGGGCATCATTGTTGGTCTGGTTCTCTTTGTGGTCACTGGAGCTGTGGTGGCTGGAGCCGTGCTGTGGAGGAAGAAGCACTCAGGTGGAAAAGGAGGGAGCTACATTGAGGCTGCAAGCAGTGACAGTGCCCAGGGCTCTGATGTGTCCCTCACGGCTTCTAAA

>Rhsi_G86_pr_NW017740079_101301_102581

NACCTGCGCTCCTGGACCGCGGCGGACTCGGCGGCTCAGATCACCCGGCGCAAGTGGGAGGCGGCCGATGTAGCACAGGGTTTCAGAGAGTACTTTGAAGGCACGTGCCTGGAGTGGCTCCTCAAATACCTGGAGAACGGGAAGGAGACACTGCAGCGCGCAGACCCTCCAAAGGCACACGTGACCCACCACCGCACCTCTCACCATGATGTCACCCTGAGGTGCTGGGCCCTGGGCTTCTACCCTGCGGAGATCACCCTGACCTGGCAGCGTGATGGGGAGGATCTGACCCAGGACACGGAGCTCGTGGAGACCAGGCCTGCGGGGGACGGGACCTTCCAGAAGTGGGCAGCTGTTGTGGTGCCTTCTGGAGAGGCTGGCATGGTGGTGGGTGTCATTGCTGGGCTGGTCCTCCTTGGAGCTGTGGTGGCTGGAGCTGTGCTGTGGAGGAGGAAGCGCTCAGGTAGGGAA

>Rhsi_G30_pr_NW017740079_20467_23315

ATGGCCCCCAGAACCCTCCTCCTACTGCTGTCGGGGGCCCTGATCCTGACTGGGACCCGGGCGGGCTCCCACTCCCTGAGATATTTCATCACCACCTGGTCCCGGCCCGGCCGCGGGGAGCCCCGCTTTGTCGCCGTCGGCTACGTGGACAACACGCAGTTCGTGCGGTTCGACAGCGACGCGGCGAGTCCGAGGATGGAGCCGCGGGCACCGTGGATGGAGCAGATGGAGCCGGGGTACTGGGAAGTAGAGACGGGGCACGTTGAGGACACCGCACAGACTGCCCGAGTGGGGCTGAACACCCTGCGCAGCTACTACAACCAGAGCGAGGCCGGGTCTCACACATTCCAGTGGATGTACGGCTGCGACGTGGGGCCGGACGGGCGCCTCCTCCGCGGTTATGAGCAGTTTGCCTACGACGGCGTCGACTACCTAGCCCTGAACGAGGACCTGCGCTCCTGGACCACGGCGGACACGGCGGCTCAGATCTCCAAGAGGAAACTTGAGATGGCACATGAGGCAGAGATCCACAGGAACTATCTGCAGAGGGAGTGCGTGGAGTGGCTCCTCAGATACCTGGAGAATGGAAAGGAGATGCTGCAGCGCGCAGACACCCCAAAGACACACGTGACCCACCACCGCACTTCTGACCGTGACGTCACCCTGAGGTGCTGGGCGCTGGGCTTCTACCCTGCGGAGATCACCCTGACCTGGCAGCGTGATGGGGAGGACCTGACCCAGGACACGGAGCTCGTGGAGACCAGGCCTGCGGGGGACGGGACCTTCCAGAAGTGGGCAGCTGTGGGGTTGCCTTCTGGAGAGGAGCAGAGATACACGTGCCACGTGCAGCACGAGGTTCTCCCCCAGGCGGTGACA

>Rhsi_G84_pr_NW017740079_7660_9748

ATGGGGCTCCGAACGCTGCTCTTGCTGTTCTCTGCGGCCCTGACCCTGACCGAGACCTGGGCGGGCTCCCACTCCCTGAGGTATCTCCATTCCGCTGTGTCCCGGCCCGGCCGCGGGGAGGCCCTCTACGTCTCGGTCGGCTACGTGGACGACACGCAGTTCCTGCGGTTCGAGAGCGACGCGGTGCGTCCGAAGGTGGAGCCGCGCGCGCCATGGGTGGAGCAGGAGGGACCGCAATTTTGGGAAGCTCAGACTGAGATCGCCAAGGTCCACGCCCAGACTTTGCGGTCGAACCTGGAGACGGCCCGGGGCTACTACAACCAGAGCGAGTCAGGGTCTCACACCTTCCAGTGGACTTCTGGCTGCGACGTGGGGTCGGATGGGCGCTTCCTCCGCGGTTATGAGCAGTTCGCCTACGACGGCGCCGACTACATCGCCTTGAACGAGGACCTGCGCTCCTGGACCGCGGCGGACGAGGTGGCTCACATCACCCGGCGCAAATGGGAGGCGGCCGGGTTAGCTGAGCGCTACCGGGCATACCTGGAGAGGGAGTGTGTGGAGTGGCTTCGCAGGTACCTGGAGAACGGGAAGGAAACGCTGCAGCGCGCAGATCCCCCAAAGACACACGTGACCCACCACCGCACCTCTGAC

>Rhsi_G11_pr_NW017746727_112_1228

GACGGGCGCCTCCTCCGCGGGTACGTTCAGGAAGCCTACGACGGCGCCGACTTCATCGCCCTGAACGAGGACCTGCGCTCCTGGACCGCGGTGGACACGGCGGCTGATATCTCCCGGCGCGAGTGGGAGGCGGCCGATGTGGCGGAGCAATGGAGGGGCTACTTTGAGGGCAGGTGCAGGGAGTCGCTCGGCAAATACCTGGAGAAGGGGAAGGAGACGCTGCAGCGCACAGATCCTCCAAAGACACACGTGACCCACCACCGCACCTCTGACCGTGGCGTCACCCTGAGGTGCTGGGCGCTGGGCTTCTACCCTGCGGACATCACCCTGACCTGGCAGCGTGATGGGCAGGACCTGACCCAGGACACGGAGCTCGTGGAGACCAGGCCTGCGGGGGACGGGACCTTCCAGAAGTGGGCAGCTGTGGGGGTGCCTTCTGGAGAGGAGCAGAGATACACGTGCCATGTGCAGCACGAGGGGCTGCCCAAGCCCCTGACCCTGAGATGG

>Rhsi_G39_pr_NW017742045_1731_2898

GACCCTCCAAAGGCACACGTGACCCACCACCGCACCTCTGACCGTGACGTCACCCTGAGGTGCTGGGCGCTGGGCTTCTACCCTGCGGAGATCACCCTGACCTGGCAGCGTGATGGGGAGGACCTGACCCAGGACACGGAGCTCGTGGAGACCAGGCCCGCGGGGGACGGGACCTTCCAGAAGTGGGCAGCTGTGGGGGTGCCTTCCGGAGAGGAGCACAGATACACGTGCCATGTGCAGCACGAGGGGCTGCCTGAGCCTCTGACCCTGAGATGGGAGCCGCCTCCTCAGGCCGCCATCCCCACTGGCATGGTGGTGGGCGTCACTGCTGGGCTGCTCCTCCTTGGAGCTGCTGTCACTGGAGCTGTGCTGTGGAGGAGGAAGCGCTCAGGTGTAAAAGGACAGAGCTACACTCAGGCTGCAGGTGGTGACAGTGCCCAGGGCCCTGATGTGTCCCTCACGGCTTCTAAA

>Rhsi_G64_NW017739882_60652_63688

ATGCGCGTCATGGGCCCCGGAACCCTCCTCCTGTTGCTCTCGGAGGCCCTGGGCCTGACGGGAATCCGAGAGGGCTCCCACTCCCTGACATATTTCGCCACCTCCTGGTCCCGGCCCGGCCGCGGGGAGCCCCGGTTCATCGAAGTCGGCTACGTGGACGGCTCGCAGTTCGTGCGGTTCGACAGCGACGCTGCGAGTCCGAGGATGGAGCCGCGGGCGGCGTGGATGGAGGGGCCGTGGGTGGAGCAGGTGGTGCAGGGGTACTGGGACCGGGAGACAGGACAAGCCAAGGCCGACGCACAGACTCACCGTATGAGCCTGAAAAACCTGCGCGGCTACTACAACCAGAGCGAGGCCGGGTCTCACACCCTCCAGAGGATGTTCGGCTGCGACGTGGGGCCGGACGGGCGCCTCCTCCGCGGGTACAGGCAGGACGCCTACGACGGCGCCGACTACATCGCCTTGAACGAGGACCTACGCTCCTGGACCGCAGCGGACACGGCGGCTCAGATCACCCGGCGCAAGTGGGAGGCGGCGGGTCTGGCAGAGGAGCTGAGGGGCTACTTTGAGGGCGGGTGCGTGGAGTCGCTCGGCAAATACCTGGAAAAGGGGAAGGAGACGCTGCTGCGCACAGACCCTCCAAAGACACACGTGACCCACCACCGCACCTCTGACCGTGACGTCACCCTGAGGTGCTGGGCGCTGGGCTTCTACCCTGCGGAGATCACCCTGACCTGGCAGCGTGATGGGGAGGACCTGACCCAGGACACGGAGCTCGTGGAGACCAGGCCTGCGGGGGACGGGACCTTCCAGAAGTGGGCAGCTGTCGTGGTGCCTTCTGGAGAGGAGCAGAGATACACGTGCCATGTGCAGCACGAGGGGCTGCCCGAGCCCCTGACCCTGAGATGGGAGCCGCCTCCTCAGGCCACCATCCCCGCTGCCATGATGGTGGGCGTCATTGCTGGGCTGGTCCTCCTTGGAGCTGCGGTTGCTGGAGCTGTGCTGTGGAGGAGGAAGCGCTCAGGTGGAAAAGGAGGGAGCTATGCTCAGGCTGCAGGCGGTGACAGAGCCCAGGGCTCTGATGTGTCTCTCACGGCTTCTAAAGGT

>Rhsi_G76_pr_NW017745016_172_956

GGCTCCCACTCCCTGAGGTGTTTCGGAACCACCTGGTCCCGGCCCGGTCGCGGGGAGCCCCGCTTTATCGCCTTCGTCTACGTGGACGACACGCAGTTGATGCGGTTCGACGGCAACGCAGCGAGTCCGAAGATGGAGCCGCGGGCAGCGTGGATGGAGGAGCCGTGGGTGGAGCAGGAGCTGCCGGGCTTCTGGGACCGGCAGACAGGACAAGCCAAGTTCTTCCAAAAGACTTTGCGAGCCGACCTGCAGAGCGCGCTGGAGAACCACAACCAGAGCGAGGCCGGGTCTCACACCCTCCAGGTGACGTACGGCTGCGAGATGGGACCCGACGGTCGCATCCTCCGCCGGTACCTTCAGGAAGCCTACGATGGCGCCGACTACATCGCCCTGAACGAGGACCTGCGCTCCTGGACCGCGGCGGACCAGGCGGCTGAGATCTCCAAGAGGAAACGTGAGGTGGTACAGGTCGGAGAGTATCTGAGGGACTTCGTTAAGAGCAGGTGCGTGGACTGGCTCCGCAAATCCCTGGAGAAGGGGAAGGAGACACTGCTGCGC

>Rhsi_G78_pr_NW017740189_29086_29891

GGCTCCCACTCCCTGAGGTTTTTCGGCACCACCTGGTCCCAGCCCGGCCGCGAGGAGCCCCGCTATATCGGAGTCAGCCACGTGGACGACACGCAGTTGATGCGGTTCGACGGCAACGCCGCGAATCCGAAGCTCGAGCCGCTGGCGGCGTGGCTGGAGGGGCCGTGGGTGGAGCAGGTGGAACCGGGGTTCTGGAACCGGGAGACACGACGCGCCAAGTTATACCAACGGACTTTGCGAGCGGACCTGCAGACCATGCTCAAGAATTACAACCAGAGCGAGGCCGGGTCTCACACCCTCCAGAGGATGTACGGCTGCGACGTGGGGCCGAACGGGCGCCTCCTCCGCGGGTACAGGCAGGACGCCTACGACGGCGCCGACTACATCGCCCTGAACGAGGACCTGCGCTCCTGGACCGCGGCGGACACGGCGGCTCAGATCTCCAAGAGGAAACTTCAGGTGGTACAATTGGCAGAGTCTATGATGGGCTTCGGTAAAAGCAAGTGCGTGGACTGGCTCCGCAAATCCCTGGAGAAGGGGAAGGAGACGCTGCTGCGCGCA

>Rhsi_G16_ps_NW017738920_6406001_6411617

TCAGATCCTGGCCATGGGATCCCTGAATTTATTTCCGTGGGGTACGTGGACTCTCCTCCCATTGCCACATACAACAGCGTCTCTCGGCTGAAGGAGG!GAGCCCCAGGCCCCATGGATGGTGCAGAGCACCTCGCCCTGACCGCTGGGAGAGGTACCCTCAGCTGCTGCGGGGCTGGCAGCACACGTTGTATTTATCAATGAATCGTTCCCATTATAGGAAAGCTCCCCAAGGGTTTCACACTTATCAGACGGTGACTGGCTGTGAGTTGCTGAAGGATGGAAACAGTGCAGGATTTCTGCAATGTGCGTATGACGGACAGGATTTCATTATCTTCAATAAAGATATGCTCTCCTGGACGGCTGTAGATAACGTGGCACACATCACCAAGGGGTGT!GAGAGGCCAGTCGGCACGAGTTACAGTATCAGAAGAACTGGCTGGAAAACGGATGCATCGCATGGTTGAAGAGATCCCTGGAGTGCGGGAAAGATACCCTACAAAGGACAGGGGAGCCAGTGACACAAATCCTTCCATCTCCTGTCAGTGATAGGGATTTATGAAGCACCTGTTGGGAGTTTATGGCTTTTATCCCCCCCGAAATTTCCATGATCTGGATGAGAAACGGTGAAGCAATCCTCCAAGAAATGGATTATGGAGACATTCTTCCCAGTGGGGACGGGACCTGTCAGACATGGGTGTCAGGTGAGCTGGAGACTCAGAGCAGTGACCTTTACTCCTGTCACGTGGAGCAC

>Rhsi_G112_ps_NW017740137_107103_110001

ATGCGCGTCATGGGCCCCGGAGCCCTCCTTCTGCTGCTCTCGGGGGCCCTGGCCCTGACGGGGACCCGGGCGGGCTCCCACTCCCTGAGGTATTTCCAAACCACCTGGTCCCGGCCCGGCCGCGAGGAGCCCCGGTTCATCGAAGTCGGCTACGTGGACGACACGCAGTTCGTGCGA!!CGCGGCGAGACTAAGAGAGGAGCCGCGGGCGGCGTGGATGGAGGGGCCCTGGGTGGAGCAGGTGGAGCCGGGGCACTGGGACCGGAACACGCAGAGAGCCAAGACAGAAGCACAGAATCACCGAATGTGCCTGAACATCATCCGCGGCCACTACAACCAGAGCGAGGCCGGGTCTCACACCATCCAGAGGATGTACGGCTGCGACATGGGGCCGGACGGGCGCCTCCTCCGCGGGTACTACCGGGACGCCTACGACGGCGCCGACTACCTGACCCTGAACGAGGACCTGCGCTCCTGGACCGCGGCGGACTCGGCGGCAGAGATCTCCCGGCGGAGGGTGGAGGCGACGGGTCTGGCGGAGGACCTGAGGGGCTACTTTGAGGGCACCTGCATGGAGTTGCTCGGCAAATACCTGGAGAGGGGGAAGGAGACGCTGCAGCGCGCAGATCCTCCAAAGACACACGTGACCCACCACCATACCTCTGACCGTGACGTCACCCTGAGGTGCTGGGCGCTGGGCTTCTACCCTGCGGAGATCACCCTGACCTGGCAGCGTGATGGGGAGGACCTGACCCAGGACACGGAGCTCGTGGAGACCAGGCCTGCGGGGGACGGGACCTTCCAGAAGTGGGCAGCTGTAGGGGTGCCTTCTGGAGAGGAGCAGAGATACACGTGCCATGTGCAGCACGAGGGGCTGCCTGAGCCCCTGACCCGGAGATGGGAGCCGCCTCCTCAGGCCGCCATCCCCGCTGCCATGGTGGTGGGCGTCATCGCTGGGCTGGTCCTCCTTGGAGCTGCGGTCACTGGAGCTGTGCTGTGGAGGAGGAAGCGCTCAGGTGGAAAAGGAGGGAGCTACGCTCAGGCTGCAGGTGGCGACAGTGCCCAGGGCTCTGATGTGTCTCTCACGGCTTCTAGA

1. **Pangolin *Manis javanica***

>Manjav_G15_ps_NW016575588_20891_23926

CTGCTGTTGGGGACCCTGGCCCTGACCCAGACCTGGGTGGGCTCCCACTCCCTGAGGTATTTCTACACCACCGTGTCCCGGCCTGGCCACGGGGAGCCCCGCTTCATCACTGTGGGCTATGTGGATGACACGCAGTTCATTTGGTTTGACAGCGATGCCGCGAGTCCGAGGATGGAGCCTCGGACGTGGTGGATGCAGCAGGTGGGGCCTGAATACTGGGACCGGGAGACCCGAA!!CAGGCTTTCCGAGTGGGGCTGAATGACCTACTCCGCTGTTACAACCAGAGGGCAGGGTCTGGGTCTCACACTCTACAGCAGGCATTTGGCCACAAAGTGGGGGTCGGACTGATGCTT!!CCTCCGTGGGTACTGGCAGTTAGCCTACTACAGCTCCGATTACATGGCCCTGAATGAGGATCTGCGGTCCTGGACCGCAGCGGACATGGCGGCTCAGATCACCAGGCATAAGTGGGAGGAGGACAGTGCGGTGGAGCGTATCGGGAACTACTTGGAGGGCAGGTGCATGGAGGGACTGCACAGGTACCTGGATATCTGGAAGCAAATGCTGCAGCGCACAGATCCCCCCAAAAGACATGTGACCCACCACCCCTTCTCTGACCATGTGGTCACTCTGAGGTGCTGGGCCCTGGGCTTCTACCCTGTGGAGATCACCCTGACCTGGCTGCGTGATGGGGATGACCTGACCCAGAACACAGAGCTTGTGGAGACCAGGCCTTCA!GGGATGGAATCTTCCAGAAGTGGGCAGCTGTCGTGGTGCCTTCTGGGGAGGAGCAGAGATACACATGCCATGTGTCAAGGTCAGGACTCAAGCCTGAAGTTAGGCCTTCACTTACTCTTCCCTCCCCAGAGCCACCTCAACCCACCATCCCCATCATAGGTCTCATTGCTGGCCTGGTCCACTTCGGGGTCACTGGGGCCATGGTGGTTGTAGCTGTGATATGGAGTAAGAAGTGCTCAGGAGGAAAAGAAGGGAACCATGCTGAGGCTGCAAACAGTGACCAGGGCTCTGATGTGTCTCTCACAGATGCTAAA

>Manjav_G2_NW016583632_21059_24130

ATGGCTCCCTGGGCCCTTCTCTTGCTGCTGTCGGGGACCCTGGCCCTGACCCAGACCCGGGCTGGTTCCCACTCCCTGAGGTATTTCTACACCGCCATGACCCGGCCCGGCCGCGGGGAGCCCCGCTTCGTCGCCGTGGGCTACGTGGACGATACGCAGTTCGTGAGGTTCGACAGCCACTCCGCGAGTCCGAGGATGGAGCCCCGGGTGCCGTGGATGCAGCAGGAGGGGCCTGAGTACTGGGACCGGGACACGCGGATCTACAAGGACACCGCACAGAATTTCCGAGTGGGGCTGAACAACCTGCGCAGCTACTACAACCACAGCGAGGAGGGGGTGGGGGTGGGGGCGGGGCCAGGGTGTCACACCTACCAGTGGCTCTGTGGATGCGACGTGGGGCCAGATGGGCGCCTCCTCCGCGGGTACAGCCAGTTCGCCTACGACGGTACCGATTACCTCACGCTGAACGAGGACCTGCGCTCCTGGACCGCGGCGGACACGGTGGCTCAGATCACCCAGCGCAAATGGGAGGCTGCCGGTGGGGCAGCGGATCAGAGGGCCTATCTGGACGGGGAGTGCGTGGAGGGGCTGCGAAGGTACCTGGAGAAAGGGAAGGAGACGCTGCAGCGCGCAGAACCCCCAAAAAGACACGTGACCCACCACCCCTTTTCTGACCGTGAGGTCACCCTGAGGTGCTGGGCCCTGGGCTTCTACCCTGCGGAGATCACCCTGACCTGGCAGCGTGATGGGGATGACCTGACCCAGGACACAGAGCTTGTGGAGACCAGGCCTTCAGGGGACGGAACCTTCCAGAAGTGGGCAGCTGTGGTGGTGCCTTCTGGGGAGGAGCAGAGATACACATGCCATGTGCAGCATGAGGGGCTGCCCGAGCCCATCACCCTGAGATGGGAGCCACCTCAGCCCACCATCTCCCTCGTGGGCCTCATTGCCGGCCTGGTCCTCTTTGTGGTCACTGGAACCGCGGTGGCTGGAGCTGTGATGTGGAGGAAGAAGCGCTCAGGAGGAAAAGGAGGGAGCTATGCTCAGGCTGCAAGTGTGAACAGTGACCAGGGCTATGATGTGTCTCTCACGGCTGCTAAA

>Manjav_G5_NW016529316_64027_67050

ATGGCGCCCCGAGCCCTCCTCCTGCTGCTGTCGGGGGCCCTGGCCCTGACCCAGACCCGGGCGGGCTCCCACTCCCTGAGGTATTTCGACACCGCCGTGTCCCGGCCCGGCCGCGGGGAGCCCCTCTACATCTCCGTGGGCTACGTGGACGACACGCCGTTCGTGCGGTTCGACAGCGACGCCGCGAGTCCGAGGATGGAGCCCCGGGTGCCGTGGATGCAGCAGGAGGGGCCGGAGTACTGGGACCGGGAGACGCGGAACGTCAAGAGCAGCGAACAGACTTTCCGAGTGAACCTGAACACCGCCCGCGGCTACTACAACCAGAGCGAGGCCGCAGGGTCTCACACCGTCCAGTGGATGTACGGCTGCGACATGGGGCCGGACGGGCGCCTCCTCCGCGGGTACAGACAGTTAGCCTACGACGGCGCCGATTACATCGCCCTGAACGAGGACCTGCGCTCCTGGACCGCGGCGGACACGGCGGCGCAGATCACCCGGCGCAAGTGGGAGGCTGCCGGTGCGGCGGAGCACTTCAGGAACTACGCGGAGGGCAGGTGCCTGGAGGGGCTGCGCAGGTACCTGGAGATCGGGAAGGAGACGCTGCAGCGCGCAGAACCCCCAAAAAGACATGTGACCCACCACCGCTTCTCTGACCGTGAGGTCACCCTGAGGTGCTGGGCCCTGGGCTTCTACCCTGCGGAGATCACCCTGACCTGGCTGCGTGATGGGGACGACCTGACCCAGGACACAGAGCTTGTGGAGACCAGGCCTTCAGGGGACGGAACCTTCCAGAAGTGGGCAGCTGTGGTGGTGCCTTCTGGGGAGGAGCAGAGATACACATGCCATGTGCAGCATGAGGGGCTGCTCAAGCCCATCACTCTGCAGTGGGAGCCACCTCAGCCTACCATCCCCCTCGTGGGCCTCATTGCCGGCCTGGTCCTCTTCGGGGTCACTGGAGCTGCGGTGGCTGGAGCTGTGATGTGGAGGAAGAAGCGCCCAGGAGGAAAAGGAGGGAGCTATGCTCAGGCTGCAAGTAATGACAGTGACCAGGGCTCTGATGTGTCTCTCACGGCTGCTAAAGGT

>Manjav_G14_NW016535030_22825_26228

ATGGCGCCCCGAGCCCTCCTCCTGCTGCTGTCGGGGGCCCTAGCCCTTACCCAGACCTGGGCGGGCTCCCACTCCCTGAGGTATTTCCACACCGCCGTGTCCCGGCCCGGCCTCGGGGAGCCCTTCTACATCTCCGTGGGCTACGTGGACGACACGCCGTTCCTGCGGTACAACAGCGACGCTGCAAATCCAAGGGTGGAGCCTCGGGCGCCATGGATGGAGCAGGAAGAGCCACAGTTTTGGGACCGGCAAACCAGTATTGCCAAACGGCATTCTCAGACCTGCACATTCAATCTGAAGACTGCCCTTGGCTACTACAACCAGAACGAGTCCGTGCCTCACGCTTTCCAGTGGCTGTCCGGCTGCTCTGTGGGGCCTGACGGGCGCCTCCTGCGTGGCTATGAAGAATTTGCCTACGACGGCGCGGATTACCTCGCCCTGAATGAGGACTTGCGCTCCTGGACCGCGGCGGACGCGGCGGCTCAGATGACCCGGCGCAAGTGGGAGGCAGCCGGTATGGCGGAGCACTACCGGCTCTATTTGCAGAGGGAGTGCGTGGAGTGGCTCCGCTGGTACCTGGAGAAAGGGAAGGAGGCGCTGCAGCACGCAGACCCCCCAAAGACACATGTGACCCATCACCCCACCTCTGACAGTGAGGTCACCCTGAGGTGCTGGGCCCGGGGCTTCTACCCTGCGGAGATCACCCTGACCTGGCTGCGTGATGGGGACGACCTGACCCAGGACACAGGGCTTGTGGAGACCAGGCCTTCAGGGGACGGAACCTTCCAGAAGTGGGCGGCTGTGGTGGTGCCTTCTGGGGAGGAGGAGCAGAGATACACATGCCATGTGCAGCATGAGGGGCTGCCTGAGCCCGTCACCCTGAGATGGGAGCCACCTCAGCCCACCATCTCCCTTCTGGGCCTCATTGCTGGCCTGGTCCTCTTTGTGGTCACTGGAACTGCGGTGGCTGGAGCTGTGATGTGGAGGAAGAAGCGCTCAGGAGAAGAAGGAGGGAGCTATACTCAGGCTGCAACCCCAGGTAGTGACAGTGCCCAGAGCTCTTATGTGTCTCTCATGGCTCCTAAAGAT

>Manjav_G11_pr_NW016599696_3_1592

GCAGGCTCCCACTCCCTGAGGTATTTCGGTACCGCCGTGTCCCGGCCCGGCCGCAGGGGGCCCCGCTTCGTCGCTGTGGGTTACGTGGACGACACGCAGTTTGTGAGGTTCGACAGCAATGCCGCGAGTGCGAGGATGGAGCCGCTCGCGCGGTGGGTGGAGCAGGAGGCGCCGGAGTATTGGGACCGGGAGACGCGGATCGCCAAGGGTCACGAACAGAATTTCCGAGTGGGGCTAAACAACCTGCGCAGCTACTACAACCAGAGCGCGGCCGAGTCTCACACCCTCCAGAAGATGTGTGGATGCGACCTGGCGCCGAACGGGTGCCTCCTACGCGGGTATTTCCAGTTAGCCTACGACGGCACCGATTACCTTACCCTGAACGAGGACCTGAGCTCCTGGACCGCGGCGGACACGTCGGCTCAGATCGCCCGGCGCAAGTGGGAGGTGGGCCGTGAAGCGAAGAGTCAAAGGAACTACTTAGAAGGAAGGTGCGTGGAGTGGCTGCGCAGGTACCTGGAGATCGGGAAGGAGACGCTGCAGCGCGCAGGAACCCCCGAAAGACACGTGAGCCACCACCCCTTCTCTGATGGTGAGGTCACCCTGAGGTGCTGGGCCCTGGGCTTCTACCCTGCGGAGATCACCCTGACCTGGCTGCGTGATGGGGAAGACCTGACCCAGGACACAGAGGTTGTGGAGACCAGGCCTTCAGGGGACGGAACCTTCCAGAAGTGGGCGGCTGTGGTGGTGCCTTCTGGGGAGGAGCAGAGATACACATGC

1. **Killer whale *Orcinus orca***

>Orcorc_G1_NW004438695_503248_506150

ATGGCATCGGGAGCCCTCCTTCTGCTCCTCACGGGGGCCCTGACCCTGACCGAGACCTGGGCGGgctcCCACTCCCTGAGGTATTTCTACACCGGGGTGTCCCGGCCGGGCCGCGGGGAGCCCCGCTTCATCGCCGTCGGCTACGTGGACGACACGCAGTTCGTGCGGTTCGACAGCGACGCCCCGAATCCGAGGGAAGAGCCGCGGGCGCCGTGGGTGGAGCAGGAGGGGCCGGAGTACTGGGATCGGAACACGCGGATCTACAAGGAGGCCGCACAGATTTACCGAGTGGACCTGAACACCCTGCGCGGCTACTACAACCAGAGCGAGGCCGGGTCTCACACCATCCAGGAGATGTACGGCTGCGACGTGGGGCCGGACGGTCGCCTTCTCCGCGGGTACAGCCAGGACGCCTACGACGGCGCCGATTACATCGCCCTGAACGAGGACCTGCGCTCCTGGACCGCGGCCGACGCGGCGGCTCAGATCACCAAGGGCAAGTGGGAGGCGGCcggtgctgcggagcaactaaggaaCTACGTGGAGGGCACCTGCGTGGAGTGGCTCCTCAGATACCTGGAGACCGGGAAGGACACGCTGCAGCGCGCAGACCCTCCAAAGACACACGTGACCCACCACCCCATCTCTGACCGTGAGGTCACCCTGaggtgctgggccctgggcttcTACCCTAAGGAGATCTCACTGACCTGGCAGCGTGATGGGGAGGATCAGACCCAGGACATGGAGCTTGTGGAGACCAGGCCTTCAGGGGACAGAACCTTCCAGAAGTGGGCGGCCCTGGTGGTGCCTTCTGGAGAGGAGCAGAGATACACGTGCCACGTGCAGCACGAGGGGCTTCAGGAGCCCCTCACCGTGAGATGGGaACCTCCTCAGTCCACCGTCCCCATCATGGTCCTCATTGTTGGCCTGGTTCTCTTGGTGGTCACTGGAGCTGTGGTGGCTGGAGCTGTGATCTGGAGGAAGAAGCACTCAGGTGAAAAAGGAGGGAGCTACGCTCAGGCTGCAAGCAGCGACAGTGCCCAGGGCTCTGATGTGTCTCTCACGGATCCTAAA

>Orcorc_G17_NW004438695_36273_39163

ATGGCACCGCGAACCCTCCTCCTGCTCCTCTCCGGGGCCCTGGCTCTGACCGAGACCTGGGCGGGTGAGTGCGGGGTCGGGTATTTCAACACCGGGGTGTCCCGGCCGGGCCGCGGGGAGCCCCGCTTCATCGCCGTCGGCTACGTGGACGACACGCAGTTCGTGCGGTTCGACGGCGACGCCCCGAATCCGAGGATGGAGCCGCGGGCGCCGTGGGTGGAGCAGGAGGGGCCGGAGTACTGGGATCGGGAGACGCGGAACTTCAAGGACGCCGCACAGAATTTCCGAGCGGGCCTGGACACCCTGCGCGGCTACTACAACCAGAGCGAGGCCGGGTCTCACACCCTCCAGCAGATGTACGGCTGCGACGTGGGGCCGGACGGTCGCCTCCTCCGCGGGTACAGACAGGACGCCTACGACGGCGCCGATTACATCGCCCTGAACGAGGACCTGCGCTCCTGGACCGCGGCCGACGCGGCGGCTCAGAACACCAAGCGCAAGTGGGAGGCGGCcggtgctgcggagcaacaaaggaACTACGTGGAGGGCACCTGCGTAGAGGCGCTCCTCAAATACCTGGAGACCGGGAAGGACACGCTGCAGCGCGCAGACCCTCCAAAGACACACGTGACCCACCACCCCATCTCTGATCGTGAGGTCACCCTGaggtgctgggccctgggcttcTACCCTAAGGAGATCTCACTGACCTGGCAGCGTGATGGGGAGGATCAGACCCAGGACATGGAGCTTGTGGAGACCAGGCCTTCAGGGGACAGAACCTTCCAGAAGTGGGCGGCCCTGGTGGTGCCTTCTGGAGAGGAGCAGAGATACACGTGCCACGTGCAGCACGAGGGGCTTCAGGAGCCCCTCACCCTGAGATGGGaACCTCCTCAGCCCACCATCGCCATCATAGGCCTCATTGTTGGCCTGGTTCTCTTGGTGGTCACTGGAGCCGTGGTGGCTGGAGCTGTGATCTGGAGGAAGAAGCACTCAGGTGAAAAAGGAGGGAGCTACGCTCAGGCTGCAAGCAGTGACAGTGCCCAAGGCTCTGATGTGTCTCTCAGGCATCCTAAG

>Orcorc_G29_NW004438747_635557_638696

ATGGCACCGCGAACCCTCCTCCTGCTCCTCTCCGGGGCCCTGGCTCTGACCGAGACCTGGGCGGGCTCCCACTCCCTGAGGTATTTCCACACCGGGGTGTCCCGGCCCGGCCGCGGGGAGCCCCGCTTCACCGCCGTCGGCTACGTGGACGACACGCAGTTCGTGTGGTTCGACAGCGACGCCCCGAATCCGAGGAAGGAGCCGCGGGCGCCGTGGGTGGAGCAGGAGGGGCCGGAGTACTGGGAAGAGGAGACGCGGATCTCCAAGGACGCCGCACAGATTTACCGAGTGAACCTGAACAACCTGCGCGTCTACTACAACCAGAGCAAGGCCGGGTCTCACACCCTCCAGGAGGTGTACGGCTGCGACGTGGGGCCGGACGGTCGCCTCCTCCGCGGGTACAGACAGTTAGCCTACGACGGCGCCGATTACATCGCCCTGAACGAGGACCTGAGCTCCTGGACCGCGGCCGACGCGGCGGCTCAGATCTCCAAGCGCAAGTTTGAGAAAGCCGGTGCTGCGGAGCGCCACAGAGCCTACCTGGATGGGGCATGCGTAGAGGCGCTCCTCAGATACCTGGAGACCGGGAAGGACACGCTGCAGCGCGCAGACCCTCCAAAGACACACGTGACCCACCACCCCATCTCTGACCGTGAGGTCACCCTGaggtgctgggccctgggcttcTACCCTAAGGAGATCTCACTGACCTGGCAGCGTGATGGGGAGGATCAGACCCAGGACATGGAGCTTGTGGAGACCAGGCCTTCAGGGGATGGAACCTTCCAGAAGTGGGTGGCCCTGGTGGTGCCTTCTGGAGAGGAGCAGAGATACACGTGCCATGTGCAGCACGAGGGGCTTCAGGAGCCCCTCACCCTGAGATGGGaACCTCCTCAGTCCACCGTCCCCATCATGGCCCTCATTGTTGGCCTGGTTCTCTTGGTGGTCACTGGAGCCGTGGTGGCTGGAGCTGTGATCTGGAGGAAGAAGCACTCAgGTGAAAAAGGAGGGAGCTACGCTCAGGCTGCAAGCAGCGACAGTGCCCAGGGCTCTGATGTGTCTCTCACGGATCCTAAA

1. **Anteater *Myrmecophaga tridactyla***

>Myrtri_G60_PVIY010024886_22571_27432

TTGGCGCCGCGAGCCCTCCTCCAGTTGCTCTTGGGGGCCCTGGCTCTGACCCAGACCCGGGCGGGTGAGTGCGGGGTCGGGGGGGAAATGCCCCCGGACCGCGGGGATTCCCGCTACATTGAAGTCGGCTACGTGGACGACACGCAGTTCGTGCGGTTCGACAGCGACTCGGCGAGTCCGAGGATGGAGCCGCGGGCGCCGTGGGTGGAGCAGGAGGGGCCGGAGTATTGGGAGCGGGAGACGCGGATCTCAAAGCAAAACGCACGAACTTTCCGAGTGGACCTGCGGACCCTGCGCGGCTACTACAGTCAGAGCGAGGCCGGGTCTCATACTCTTCCGTGGATGTCTGGCTGCGACGTGGGGCCCGATGGGCGCCTCCTCCGCGGGTACCGTCAGGACGCCTACGACGACCACGATTACATCGCCCTGAACGAGGACCTGCGCTCCTGGACGGCGGCGGACACGGCGGCTCAGATCACCCGGCGCAAATTGGAAGTGGCCAGAGATGCAGACTACTACAGGGCCTACCTGGAGGGCAGGTGCATGGAGTGGCTCCAAAGACACCTGGAGAACGGGAAGGAGACGCTGCAGCGCACAGATCCTCCAAAGACACACATGACCCACCACCCTATCTCTGACCGTGAGGTCACGCTGAGGTGCTGGGCCCTGGGCTTCTACCCGGCGGAGATCACACTGACCTGGCAGTGGGAGGGGGAGGACCAGACCCAGGACACAGAGTTTGTGGAGACCAGGCCTGCGGGGGATGGAACCTTCCAGAAGTGGGCGTTGTTGGTGCCCCCGGGAGAGGAGCAGAGATACACATGCCATGTGCAGCACGAGGGGTTGGCTGAACCCCTCACCCTGAGATGGGAGCCACCTTTGGAGTTCCCCATCCTCTTGGTGGAAATTATAGCTGGCCTGGTTCTCCTTGGAGCTATAGTGGCTGTGGTGGCTAGAGTTGTGAACTGGAGGAACAAGAGCTCAGGTAGGGAAAGGGGCAGGGGTGGAAATGGAGAGAGCCATACTCAGGCTGCTGGTGGTGACGGTTCCCAGGGCTCTGATGATTCTCTTACGGCTTCTAAA

>Myrtri_G96_PVIY010122061_1151_2148

ATGGCGCCGCTAGCCCTCCTCCTGCTGCTCTCGGGGGCCCTGGCCCTGACCCAGACCCGGGCGGGCCCCCACTCCATGAGGTATTTCCTCACCGCCGTGTCCCGGCCCGACCGCGGGGATTCCCGCTACATTGAAGTCGGCTACGTGGACGACACGCAATTCGCGTGGTTCGACAGCGACGCGGCGAGTCCGAGGATGGAGCCGCGAGCGCCGTGGGTGGAGCAGGAGGGGCCGGAGTATTGGGAGCGGGAGACGCGGCGCGCCAAGGGCTGTGCACAGACGTCCCGAGGGAACCTGCAGACCGCACTCCGCTACTACAACCAGAGCGAGGCCGGGTCGCACACCTTCCAGTGGATGTTTGGCTGCGATGTGGTGCGCGAAGGTCGCCTCCTCCGCGGGTACTATCAGAACGCCTACGACGGCCGCGATTACATCGCCCTGAACGAGGACCTGCGCTCCTGGACGGCGGCGGACACGGCGGCTCAGATCACCCGGCGCAAGTGGGAGGCGGCTGGGACTGCAGAGAACATGAGGGCCTACCTGGAGGGCCCGTGCGTGGAGTGGCTCCAGAGACACCTGGAAAACGGGAAGGAGACGCTGCAGCGC

>Myrtri_G79_PVIY010211831_3_895

CTCTCGGGGGCCCTGGCCCTGACCCAGACCCAGGCGGGCCCCCACTCCCTGAGGTATTTCAGCACCGCCGTGTCCCGGCCCGACCGCGGGGATTCCCGCTTCATCGTCGTCGGCTACGTGGACGACACGCAGTTCGTGCGGTTTGACAGCGACGCGGCGAGTCCGAGGATGGAGCCGCGGGCGCCGTGGGTGGAGCAGGAGGGGCCGGAGTATTGGGAGCGGGAGACGCAGAACGCCAAGGCCCACGCACAGACTTCACGAGTGGATCTGCAGACCGCACTCCGCTACTACAACCAGAGCGAGGCCGGGTCGCACACTCTCCAGGTGATGTATGGCTGCGACGTGGGGCCCGACGGGCGCCTCCTCCGCGGGTACCGTCAGGTCGCCTATGACGGCCACGATTACATCGCCCTGAACGAGGACCTGCGCTCCTGGACGGCGGCGGACACGGCGGCTCAGATCACCCGGCGGAAGTGGGAGGCGGCCGGGGATGCGGAGCTCCACAGGGCCTACCTG

>Myrtri_G23_PVIY010030496_15621_18400

ATGGCGCCGCGAGCCCTCCTCCTGCTGCTCTCGGGGGCTCTGGCCCTGACCCAGACCCGGGCAGGCTCCCACTCCCTGAGGTATTTCGCCACGGCCATAAGTGGGCTGGACGACCACTACATTGAAGTTAGCTATGTGGACGACACACAGTTCCTGCGGTTCGACAGCGACAGCGCGAGCGGAAGGGTAGAACCGCTGGCGCCATGGGTGGAGCAGAAGGGGCCGGAGTATTGGGAGCGCGAGACGCGAAAAGCTCGGCGGAACGCTACGAGTATGCTAGGTGACATTCAGGAACTGCGCCGCCACTACAACCAGAGCGAGGCCGGTTATCACACCTTCCAAAGGATGTATGGCTGTGACTTGGGGCCGGACGGGCGCCTTCTTCGCGGGTACTATCAGTTTGCCTATGACGGCAATGAATTCATCGCCCTGAACGAGGACCTGCGCTCCTGGAACGCGGCGGACGCGGTGGCTCAGATCGCCCAGACCTTCTGGGAGGATCAGCGTCTTGCAGATTACCTTAGGGCCTACCTGGAGGGGGAGTGCGTGGAGAGACTACAAAGATACCTAGAATATGGGAAGGAGACGCTGCAGCGCCCAGACCCGCCAAAGACACACATGACCCACCACCCAATCTCTGATCGTGAGGTCACGCTGAAGTGCTGGGCCCTGGGCTTCTACCCTGCGGAGATCACACTGACCTGGCAGCGGGACGGGGAAGACCAGACCCAGGACACAGAGTTTGTGGACACCAGGCCTGCAGGAGATAGAACCTTCCAGAAGTGGGCAGCTGTGGTGGTGCCTTCTGGAGAGGAGCAGAGATACACGTGCCATGTGCAGCATGAGGGGCTGTCTGAGCCCCTCACCCTGAGATGGGAGCCGCCATCACAGCCCACCCATCACCATACTCTAGTGGCTATGGTGGCTGGAGTTCTGATATGGAGGAAGAAGAGCTCAGGTGGAAAAGGAGGGAGATCCTGTTTTTGTTCCTCCCTAGGTGGTGACAGTGCCCAGGGCTCTGATGTGTCTTTCACAGCT

>Myrtri_G43_PVIY010123211_1_2116

CTGCGCGGCTACTACAACCAGAGCGAGGCCGCAGGGTCTCACACTCTCCAGAGGAAGTTTGGCTGCGACGTGGGGCCCGACGGGCGCCTCCTCCGCGGGTACTCTCAGCACGCCTATGACGGCCACGATTACATCGCCCTGAACGAGGACCTGCGCTCCTGGACGGCGGCGGACACGGCGGCTCAGATCACCAGGCGCAAGTGGGAGGCGGCCGGGGAGGCGGAGCCCCTCAGGGCCTACCTGGAGGGCCCGTGCGTGGAGTGGCTCCGGAGATACCTGGAGAACGGGAAGGAGACGCTGCAGCGCACAGACCCCCCAAAGGCTCACATGACCCACCACCCCATCTCTGACCGTGAGGTCACGCTGAGGTGCTGGGCCCTGGGCTTCTACCCGGCGGAGATCACACTGACCTGGCAGCGGGAGGGGGAGGACCAGACCCAGGACACGGAGTTTGTGGAGACCAGGCCTGCGGGGGATGGAACCTTCCAGAAGTGGGCGGCTGTGGTGGTTCTCCCTGGAGAGGAGCAGAGATACACGTGCCATGTGCAGCATGAGGGGCTGTCTGAG

>Myrtri_G50_PVIY010066441_6799_9474

GGGTCTCACACTCTCCAGGTGATGTTTGGCTGCGACGTGGGGCCCGACGGGCGCCTCCTCCGCGGGTACTCTAAGCACGCCTACGACGGCCGCGATTACATCGCCCTGAACGAGGACCTGCGCTCCTGGACAGCGACGGACACAGCGGCTCAGATCACCCGGCACAAGTGGGAGGCGGCCGGGGAGGCGGAGCACGACAGGGCCTACCTGGAGGGCCCGTGCGTGGAGTGGCTCCGGAGATACCTGGAGAACGGGAAGGAGACGCTGCAGCGCACAGACCCCCCAAAGACACACATGACCCACCACCCCATCTCTGACCGTGAGGTCACGCTGAAGTGCTGGGCCCTGGGCTTCTACCCGGCGGAAATCACACTGACCTGGCAGCGGGAGGGGGAGGACCAGACCCAGGACACAGAGTTTGTGGAGACCAGGCCTGCGGGGGATGGAACCTTCCAGAAGTGGGCAGCTGTAGTGGTTCTCCCTGGAGAGGAGCAGAGATACACATGCCATGTGCAGCATGAGGGGCTGTCTGAGCCCCTCACCCTGAGATGGGAGCCACCTTCGGAGTTCACCATCTCCTTGGTGGTAATTGTGACTGGCCTGGTTCTCCTTGGAGTTACAGTGCCTGTGGTGACTGGAAGGAAGAAGAGCTCAGGCGGAAAAGGAGGGAGCTACACTCAGGCTGCTAGCAGCAACAGCCCCCAGGGCTCTGATGTGTCTCTCACGGCT

>Myrtri_G44_PVIY010021519_39342_42310

CTGCGCGGCTACTACAACCAGAGCGAGGCCGCAGGGTCTCACACTATCCAGGTGATGTATGGCTGCGACGTGGGACCCGACGGGCGCTTCCTCCGCGGGTACCGTCAGGACGCCTACGACGGCCGCGATTACATCGCCCTGAACGAGGACCTGCGCTCCTGGACGGCGGAGGACATGGCGGCTCAGATCACTAGGCGCAAGTGGGAGGCGGCCGGGGAGGCGGAGCACGACAGGGCCTACCTGGAGGGCATGTGCATGGAGTGGCTCCGGAGATACCTGGAGAACAAGAAGGAGACGCTGCAGCGCACAGACCCCCCAAAGACACACATGACCCACCACCCTATCTCTGACCGTGAGGTCACGCTGAGGTGCTGGGCCCTGGGCTTCTACCCGGCGGAGATCACACTGACCTGGCAGCGGGATGGGGAGGACCAGACCCAGGACACGGAGTTTGTGGAGACCAGGCCTGCGGGGGACGGAACCTTCCAGAAGTGGGCGGCTGTGATGGTTCTCCCTGGAGAAGAGCAGAGATACACGTGCCATGTGCAGCATGAGGGGCTGTCTGAGCCCCTCACCCTGAGATGGGAACCACCTTCGGAGTTCTACGTCCCCTTGGTGGTAATTGTGGCTGGCCTGGTTCTCCTTGGAGTTACAGTGCCTGTGGTGGCTGGAATTCTGATCTGGAGGAAGAAGAGCTCAGACGGGGGAAAAGGAGGGAAGTACACTCAGGCTGCTGGTAGCGACAGTCCCCAGGGATCTGATGTGTCTCTCACGGCT

>Myrtri_G76_PVIY010026714_10276_13366

ATGGCGCCGCGAGCCCTCCTCCTGCTGCTCTCGGGAGCCCTGACCCTGTCCCAGACCCGGGCGGGCCCCCACTCCCTGAGGTATTTCAGCACCGCCGTGTCCCGGCCCGACCGCGGGGATTCCCGCTACATCGCCGTCGGCTACGTGGACGACACGCAGTTCATGCGGTTCGACAGCGACGCGGCGAGTCCGAGGATGGAGCCGCGGGCGCCGTGGATGGAGCTGGAGGGGCCGGAGCATTCGGAGGAGTACACGCGGATCGCCAAGACCAACGCACAGACTGATCGAGTGGCCCTGCGGACCCTTCGCGGCTACTACAACCAGAGCGAGGCCGCAGGGTCTCACACTCTCCAGGGGATGCTTGGCTGTGACGTGGGGCCCGATGGGCGCCTCCTCCGCGGGTACCATCAGCACGCCTATGACGGCCACGATTACCTTACCCTGAACGAGGACCTGCGCTCCTGGACGGCGGCGGGCACAGTGGCTCAGATGACCCGGCACAAGTGGGAGGCGGCCCAGTATGCAGAGTACATCAGGGCCTACCTGGAGGGCAGGTGTGTGGAGTGGCTCCAGAGATACCTGGAGAAAGGGAAGGAAACGCTGCAGCGCACAGACCCCCCAAAGACACGCGTGACCCACCACCCCATCTCTGACCGTGAGGTCACGCTGAGGTGCTGGGCCCTGGGCTTCTACCCGGCCGAGATCACACTGACCTGGCAGCGGGAGGGGGAAGACCAGACCCAGGACACGGAGTTTGTGGAGACCAGGCCTGCGGGGGATGGAACCTTCCAGAAGTGGGCGGCTGTGGTGGTTCTCCCTGGAGAGGAGCAGAGATACACATGTCATGTGCAGCATGAGGGGCTGTCTGAGCCCCTCACTCTGAGATGGGAGCCACCTTTCCATACGACCATCCCCATCCTGGGAATCGTGGCTGGCCTGGTTCTCCTTGGAGCTATCATGGCTGTGGTGACAGGATTTCTGATCTGGAGGAAGAAGAGCTCAGGTGGAAAAAGAGGGAGCTACACTCAGGCTGCTGGTGGTGACAGTACCCAGGGCTCTGATGTGTCTTTCACAGCTTCTAAAGGT

>Myrtri_G92_PVIY010051678_6588_9526

ATGGCGCCGCGAGCCCTCCTCCTGCTGCTCTCGGGGGCCCTGGCCCTGACCCAGACCCGGGCGGGCCCCCACTCCCTGAGTTATCTTCGCACCGTGGTGTCCCGGCCCGACCGCGGGGATTCCCGCTTCATCACCGTCGGCTACGTGGACGACACGCAGTTCGTGCGGTTCGACAGCGACGCGGCGAATCCCAGCATGCAGCCGCGGGCGGCGTGGGTGGAACAGGAGGGGCCGGAGTATTGGGAGCGGGAGACGGAGATCTCAAAGCAGAACGCACAAAATTACCAAGTGGCCCTGCAAAACCTGCGGGGCTACTACAACCAGAGCGATGCCGGTTCTCACACCTACCAGAGTATGTATGGCTGCGTCATGGGGCCCGACGGGCTCCTCCTCCGCGGATACTCTCAGTACGCTTATGACGGCCACGATTACATCGCCCTGAACGAGGACCTGCGCTCCTGGACGGCGGCGGACACGGCGGCTCAGATCACCCGGCGCAAGTGGGAGGCGGGCAGGTGTGCGGATCACCGCAGGGCCTACCTGGAGGGCACGTGCGTGGAGTGGCTCCAGAGACACTTGGAGAACGGGAAGGAGACGCTGCAGCGCACAGACCCCCCAAAGACACAGGTGACCCACCACCCCATCTCTGACCGTGAGGTCACACTGAGGTGCTGGGCCCTGGGCTTCTACCCGGCGGAGATCACACTGACCTGGCAGCGGGAGGGGGAGGACCAGACCCAGGACATGGAGTTTGTGGAGACCAGGCCTGCAGGGGATGGAACCTTCCAGAAGTGGGCAGCTGTGGTGGTGCCTTCTGGAGAAGAGCAGAGATACATGTGCCTTGTGCAGCACGAGGGGCTGTCTGAGCCCCTCACTCTGAGATGGGAGCCACCTTCAGAGTCCACCATCCCCATCGTGGAAATTATGGCTGGCCTGGTTCTCCTTGGAGCTATAGTACCTGTGGTGGTTGGAATTCTGATCTGGAAGAAGAAGAGCTCAGGTCAAAAAGGAGGAAGCTATGCTCAGGCTGCTTGCAGTGACAGTGCCCAGGGCTCTGATGAGTCTCTCATGGCT

>Myrtri_G99_PVIY010058210_1918_4742

AGGGCGCCGCGAGCCCTTCTCCTGCTGTTCTCGGGGGCCCTGGCCCTGACCCAGACCCGGGCAGGCCCCCACTCCCTGAGGTATTTCCTCACCGCCGTGTCCCGGCCCGACCGCGGGGATTCCCGCTACATCCCCGTGGGCTACGTAGACGACACGCAATTCGTGCGGTTCGACAGCGACGAAGCGAGTCCGAGGATGGAGCCGCGGGCGCCGTGGGTGGAGCAGGAGGGGCCGGAGTATTGGGAGCCGGAGACTCGAAAGGCCAAGAACAACGCACAGACGTCCCGAGAGAACCTGCGGACTCTGCGAGGCTACTACAACCAGAGCGAGGCCGTTTCTCACACTGTCCAGGTCTTATCTGGCTGTGTTTTGGCCCCTAATGGGCGCCTCCTCCGCGAGTACTATCAGGTCGCCTACGACTGCCACGATTACATCGCCCTGAACGAGGACCTGCGCTCCTGGACGGTGGCGGAAACGGCGGCTCAGATCACCCGGTGCAAGTGGGAGGCGGCCGGGGATGCGGAGCAACACAGGGCCTACCTGGAGGGCAGGGGCGTGGAGTGGCTACAGAGACACCTGCAGAACGGGAAGGAGACGCTGCAACGCACAGACCTACCAAAGACACACATGACCCACCACCCAATCTCTGACCATGAGGTCACGCTGAGGTGCTGGGCCCTGGACTTCTACCCAGCGGAAATCACACTGACCTGGCAGCAGGACAGAGAGGACCAGACCCAGGACATAGAGGGCCAGACCCAGGACATGGAGTTTGTGGAGATCAGGCCTGCAGGGGATGGAACTTTCCAGCAGTGGTGGGCAGCTTTGGTGGTGCCCTCTGGAGAGGAGCAGAAATACACATGCCATGTGCAGCAGGAAAGGCTGTCTGAGCCCCTCACCCTGAGATGGGAGCCACCTTCCCATCCAACCATTCCTATTGTGAAAATTGTGGTTGGCCTGGTACTCCTTGGAGCTATAGTGGCTGTAGTTCTGATCTGGAGGAAGAAGAGCTCAGGTAGAAAAGGAGGGAGCTACACTCAGACTTCTGGCAGCAACAGTGACCAGGGCTTTGATGGGTCTCTCATGGCTTATAAAGGT

>Myrtri_G72_PVIY010007569_34001_36054

GGGTCTCACATTTTCCAGAGGATGTTTGATTGCAATGCGGAGACTGATGGGGGCCTCATCCGCCTGTATGACCAGCATACCTACGACAGCAATGATTACATTGCCCTGAACGAGGACCTGCGCTCCTGGACTGCTGCAGACATGGCGGCTCAGATCACCCTGCGCAACTGGGAATTGGGGCAATTTGCAGAGACCACTAAGGCCTACCTGGAGGGCAGGTGCATGGAGTGGCTCCAAAGATATCTAGAGAATGGGAAGGAGACTGGGCAACACAGAGACCCCCCAAAGGCACACATGACCCACCACCCCATCTCTGACCGTGAGGTCACGCTGAGATGCTGGGCCCTGGGCTTCTACCTGGCGGAGATCACATTGACCTGGCTGTGGGATGGAGAGGACCAGACCCAGGACACAGAGTTTGTAGAGACCAGGCCTGTGGGGGATGGAACCTTCCAGAAATGGGCAGTTGTCATGGTGCCTTCTGGAGATGAGCAGAGATACACGTACCATGTTTGGCATGAAGAATTAGCTGAGCCCCTCACCCTGAGATGGGAGCCACCTTTGAGGCCCACCATCCCCATTCTGGTAATCATGGCTGTCCTGGTTCTCCTTGGAGCTATAGTGGCTGTGATCTGGAAGAAGAAGAGCTCAGGTGGAAAAGGAGAGAGCTACACTCAGGCTGCTGGTGGTGACAGTGCCCAGGGCTCTGATGTGTCTCTCACAGCT

>Myrtri_G80_PVIY010079135_4399_6034

TCTCACTCTCTGAGCTACGACCTTAAGGCAGTGTCCGCGCCCAGCCCTGGGCAGCCGGAGGTGACAGCCCTCGGCTACCTGGACGGCCGCCTCTTCATCGTCTACGACAGCAGGAGCCACCTTGCTCGGCTCAGGGGCCCCGCGGACGCGGAGCTGGCCTCGCTGCTGGTAGCCAGGGAAACTGCGTTCCTTGAGGGTCGCGCACAGGAGGTGCTTTGGGGTCTGAAGACCACCATGGGCTACAAGGAGCTCAGCGCCCCAGGGCCCCATACACTACAGCTGAGTCACGGCTGCGAACTGGGCAGCAGCGGTCGACTCTGGCGCTCCGGCTACGACGGGGAGGGCTTCACCGCCTACGACCCCGCGACCTGGGCCTGGGGCGCGGCGCTCCCGGAGCCCGAGAGGCGGCGGCGGCAGCTGGTGGTCGGGCGCCGCCGGTCTGCGGTGCGCGCGTGGCTGGAGCGCGAGTGCGGGGCGGCGCTGCGGCGCTGGCTGGCGGCGGGGCTCGAATCCAGTGCCCCCCACCCCCCACCCCGGGTTGTGGTCACCCAAGGGAAGGTCCCTGAAGGCCATCACACCCTGAAATGTAGAGCATTTGGCTTCTCACCTGCCAACATCACTCTGACTTGGCTGCAGGAAGGCCAGGAGCTGACCACGGACTCAGGACTCAGGGAGACCCGACCTGCAGGGAACGAAACCTCCCAGAGCTGGGCAGCTGTGGACGTCCTCCCCGGAAAGGAGCTGAGATACACCTGC

1. **Lesser hedgehog tenrec *Echinops telfairi***

>Echtel_G5_NW004558850_59253_77453

ATGTCTGGgatcctcctcctgctgctgctctgcgAGGCGGTGGTCCTGAGCCAGACCCAGGCAGggccCCACTCGCTAAGGTATTTCCTCACGGCCATATCGCGACCCGGCCGCGAGGAGCCCCGCCTCATCGCCGTCGGTTACGTGGACAGTTTGCAAGTCGCGCGGTTCGACAGCGAGGCTCCGAATCCAAGGGCGGAGACGCAGGCTCCCTGGATGAACCTCCAGGAGCCCGAATTTTGGGAGGGGCAGACCCGGATCGCCAGGGTCCGCGAACAAACGTACCGGGAGTACCTGCGGGATTTGCGCGACTATTACAACCAGAGCAACTCTGTGTCTCACACCTACCAGGGTATGTATGGCTGTGATGTGGGGCCTGATGGGCGCCTCCTCCGTGGGTACAATCAGCGAGCCTACGATGGTGACGATTACATCGCCCTGAATGAGGACCTGCGCTCCTGGACAGCTGCAACCACAGCTGCTCAGCTCACCAAGAGAAAGTGGGAAGAGACTGGTTTGGCAGAGCGCTACAGGGCCTACTACTTGAAGGGCCGGTTCCTGGAGTGGCTGCGTAGATTCCTGGAGAGTGGGAAGGAGATGCTGCAGCGCACGGAATCACCAAAGACACATGTGACTCATCACCCCATCTCTGAAGACGAGGCCACACTGAGGTGCTGGGCCCGGGGCTTCTACCCCTCAGAAATCACCCTGACCTGGCAGCGGGACCAGGAGGACCAGCTCCAGGACACAGAGCTTGTGGAGACCAGGCCTTCCGGGGATGGAACCTTCCAGAAGTGGGCAGCCATACTGGTTCCCATCGGAGAAGAGCAGAGATATACATGCCGTGTGCAGCATGAGGGGCTGCCTGAGCCCCTCACCCTGAGATGGAAACGGTCTTCCAAGCCCACCGCCTCCATCACAGGAATCATTGTTGGCCTAATTCTCCTAGGAACTGTAATCGTTGGAGCTGCAGTGGCTGCTGCTGTGATTTGGAGGAAGAAATCAGGTGGAAAAGGAGGGAGCTATGCTCAAACTGCATgctatGACAATGCCCAAGGCTCTGAagtgcccctcccctct

>Echtel_G59_NW004558850_21387_32497

ATGGCGCTCCGGACGTGCCTTCTGTTGCTCTTGGAGTTGGGGCTTCTGGTCGTGACCCGGACACGGGCGGgctcccaCTCCCTGCGGTATTTCATCACCGCCGTGTCCAGGCCTGGGCGCGCGCAGCCCCACCTCATGGTCGTCGGCTACGTGGACGACACGCAGTTCGTGCGCTTCGACAGCGACGTCCCAAACGCGAGAATGGAGGCGCGGGCGCCGTGGATGGAGCTCATGGAGAAGGAGTTCTGGGACGAGCAGACGCGCTTTGCTAAGATCCGGGAACAGACTTACCGCCAGTACCTGCGGAGCCTGCGGGAGTACTACAACCAGAGCGACTACTTGTCTCACACCTACCAGGGTATGTATGGCTGTGACGTGGGGTCTGATGGACGCCTCCTCCGTGGATACAATCAGCGAGCCTACGATGGTGAGGATTACATCGCCCTGAATGAGGACATGCGCTCCTGGACTGCTGCTGATGAGTCTGCTGAGACCACCAAgagaaagtgggaagagaacgGTTTGGCGGAATGCTACCGGGCCTACTACGTGGAAGACAGGTTCATGGAGTGGCTGCGTAGATACTTGGAAAGTGGGAAAGAGATGCTGCAGCGCACAGATGCCCCAAAGACACACGTGACTCGCCACCCCATCTCTGAACAAGAGGCCACACTGAGGTGCTGGGCCCGGGGCTTCTACCCTTCAGGAATCACCCTTACCTGGCAGCGGGACCAGGAGGACCAGATCCAGGATGTGGAGCTTGTGGAGACCAGGCCTTCTGGGGATGGAACCTTCCAGAAGTGGGCAGCCATAGTGGTTCCCATTGGAGAAGAGCAGAGATACACATGCCGTGTGCAGCATGAGGGGCTGCCTGAGCCCCACATCCTGAGATGGGGACAGTCTACTGAGCTTACTGAGCCCCTCTTGGGGGCTACTAGCCTGGTCCTCCTTGGAGCAGTGGTCCTTACAACAGTTGTGTTTGTGGTTGTGATCTGGAAGAGGAACAATTCAGGGGCAAAACAAGGGAGCTATGCCCAAGCTGCAATCAGTGACAGTGCCAAGAGTTCCGAGGTGTCTCTCACAGGC

>Echtel_G36_NW004558756_10531613_10536017

ATGGCTCCCTGggccctcctcctgctgctctcgGGGGTCTCGGTCCTGACCCCGACCCGGGCGGGCTCCCACTCCCTGCGCTATTTACGCACCACCATGTCCCTGCCGGACGCCCCCGAGCCCCACTACATTGAAGTCGGCTACGTGGACGACACGCAGTTCGTGCGCTTCGACAGCGAAGCTCCGAACCCGAGGCTGGAGCCGCGGGCGCCTTGGATGGAGCCGACGGAGCCCGAGTACTGGGAAAAGGAGAGCAAGCGCGCCAGCCGCCGGGCCCAAGGTCACATAGATGACCTGCGGGGGCTGACTGTCGTCTACAACCAGAGCCAGTCAGGGGTGGGGTCAGTGTCTCACACCCTTCAGGTGATGCGTGGCTGCGACACGGGGCCGGATGGACACCTCCTCCGGGGGTACCTGCGGTGGGGCTACGACGGCGCCAATTTTCTCACCCTGAATGAGGACCTGAGCTCCTGGACCGCCGCAGACGAGACAGCTGAGATCACGAAACGCTTCTGGGAAAAGGTTCGACTGGCAGAGATTGAGAAGGAGTACTTTGAGGGCACGTGTGTAAAGGATCTCCGCCGTTAcctggagaagaggaaggagatgtTGCAGAGCGCAGATCCCCCAAAGGCACATATAACCCACCACCCCATCTCTGATGAAGACGCGGTCACACTGAGGTGTTGGGCCCGGGGCTTCTACCCTGCGGAGATCACCCTGACCTGGCAGCGAGACGGGGAGCACCAGGCCACGGAGCTGGTAGAGACCAGGCCTGCGGGGGACGGAACCTTTCAGAAGTGGGCGGCTGTGGTGGTGCCCTCTGGAGAGGAGCAGAGATACACGTGCCAAGTGCAGCACGAGGGGCTGCCTGAGCCCCTCACCCTGAGATGGGAGTCCTCTGCTCAGTCTGCCAGCCCCGTCATGGGAGTCATTGTGGGCCTCGTTTTCCTTGTGCTTGTCAGAGCTGTGGTCACCGGTGGTGTGGTCGGGAGGAGGAAGCTCTCAGGTAAGCTAAGGGGTGGGTTTCAAGGCCCAGGCAAGGACAGTGCCTCGGGCGGTAATGTCTCTCTCACTGCT

>Echtel_G97_ps_NW004558702_74428392_74431972

ACCCATGTTTCCGCAGGGAGCCACACTCTTCACTACCACTATCTGGCTCTGTCAGAACCAGGTCCAGATCTGCCCCCGTTTCTGGCAGTGAGCTATGTGGACGACCAGCCCTTCATCCGCTA!GGACAGCCAAGTGGGCAGGGCAGAACCCCAGGCTCCTTGGATGGCTCCCGTGGACGCCCATACTGGGAACAGGAGACCCTGAAGCAGAGGATGTGGGGAAAGGTGCAGCAGGTGGAGATGTGGACAGTGATGGGCTACCACCAGAGCAGGGGCAAGCACAGTGCTCAGCGCATGTTCGGCTGCACCATGCAGGAGGATGGCCGCTCCAGCAGTTTCTGGCAATTTGGCTTCAATGGGCAGGACCACCTGTCCCTGGACCTGGAGACCCTGAGCTGGGTGTCAGCCCAGCCGGCAGCCTTTCAGACAAAGCGCTGGTGGAGACAGAGC!ACTGCTACGCTGAGTACAACAAAGCCTACCTGGAAGGCCTCTGCCTCATCTCCCTGCGCAGGTACCTGGAGCTCGGAGGCCAGAGTCTGACCCAGACAGAGCCGCCCAAGGTGCAGGTGACGCGGCACATGGCTCAGGATGGCAGGGCCACGCTGAGGTGCTGGGCCCTGGGCTTCTTTCTACCCATGGGGCATCTCCATGAGCTGGTGGCTGGCCAGGTGGAGCTGGCCCTAGAGACTGAGCATGTGGAGACATGCCCCAGTGGGGATGGCACCTACCAGGAGTGGGCAGCTGTGCAGGTGCCTGCCGGGAAGGAAACCCAGTACACCTGCCACGTGCAGCACCCAGGCCTGAACCACACACTCACTGTGACCTGGGCGTCACCTTCTGGTCCAAGTCTTACTGtc

>Echtel_G37_pr_NW004558722_9953061_9957976

tcttttacaGGGCCTTACTCTCTGTCCTTCCTTTACACTGGGCTATCCAAGCCCAGGGAAGGCTTCCCCAGCTTTCAGGCCATGGCCTACCTCAACGACCAGCCCTTCTTCCACTACAATAGTGACAGCAGAAAGGCTGAACCCCTAGGACCTTGGAGTCAGGTGGAAGGAATGGAGAACTGGGAGAAGGAAAGCCAACTTCAGAAGGCCCGGGAGGACATTTTCATGGTGACCCTGAACGACATCATGGACTATTACAAGGACAGAGAAGGGTCTCACACCTTTCAGGGAATGTTTGGTTGTGAAGTTCGGAACAACAAAAGCAGTGGCGCATTCTGGAGGTATGCCTATGATGGACGAGACTTCATTGAGTTCAACAAAGAAATTCCAGCCTGGGTTCCTCTGGACCCAGCAGCCCAAAACACCAAGCAGAAGTGGGAGGCAGAAAAAGTCTATGTGCAGCGGGCCAAGGCCTACCTGGAGGAAGAGTGTCCTGAGATGCTGCAGACATACCTGGAATACAGCAAGACTCACCTGGACCAACAAGATCCTCCTTCTGTGTCACTCACCAGCCACACCAGCCCGGGGAAGGCGAGGAGCCTCAAATGCCTGGCCTATGACTTCTACCCCCGAGAAATTGATATGTACTGGACTCGGGCCAGTGAGGTGAAGATGTCTGAGACAGAGAAAGATGTTCTTCCCAGCGGAAATGGTTCTTACCAACTTTCGGTGGTGGTGGAAGTCCCTCCTGAAGACAGAGAAACCTACACCTGCCACGTGGAACACAGTAGAATAAGCCAGTCTCACAGAGTGCTGTGGGAT

>Echtel_G38_NW004558765_8422443_8457432

ATGGCGCCCCGgatcctcctcctgctgctcctggggCTGCTGCTTCTGACCCAGACCCGGGCGGGCTCCCACTCGCTGAGATATTTCCACACCTCCGTGTCCCGGCCGGGCGGCGGGGACCCCCGCTTCATCGTCGTCGGCTACGTGGACGACACGCAGTTCGTGCGCTTCGACAGCGACGCCCCGGAACCCAGGATGGAGCCGCGCGCGCCGTGGATGGAGCAGGAGGGGCCCGAGTACTGGGAGCGCGAGACCGCGAACTGCAGGAGCAAAGCACAGACCCACCGAGTGAACCTGCGGACCCTGCGCGGCTACTACAACCAGAGCGAGGCCGGGTCTCACACCATCCAGAGGATGTCCGGCTGCGACCTGGGCCCCGACGGGAGCCTGCTCCGCGGGTACGAACAGCAAGCCTACGATGGCGCAGACTACCTCGCCCTGAACGAGGACCTGCGCTCCTGGACGGCGGCGGACACGGCCGCGCTCATCACTAAGCGCAAGTGGGAGGCGGCCGGGGAGGCGGAGCGCTTCAGGGCCTACATGGAGGGCTTGTGTGTGGATTACCTCCTCAGATACctggagaacgggaagggaaccCTGCAGCGAGCAGAGCCTCCGAAGACCCAGGTGACCCACCACCACGAACGCCATGTCCCTGAGGGAGAGGTCACACTGAGGTGCTGGGCCCGGGGCTTCTACCCGGCGGACATCGTCCTGACCTGGCAGCGGGACGGCGAGGACCAGACCCAGGACATGGAGCTGGTGGAGACGAGGCCTGCGGGGGACGGGACCTTCCAGAAGTGGGCGGCTGTGGTGGTGCCCTCCGGGGAGGAGCAGAGATACACGTGCCGAGTGCAGCACGAGGGGCTGCCTGAGCCCCTCACCCTGAGATGGGAACTGCCTTCCCAGCGCACCATGCCCATTGTGGGAATAGTTGTTGGCCTGGTTCTTCTCGCAGCTGTGGTCATTGGAGCTGTGGTTGGATTTGTGATCTGGAGGAAGAAGTGCTCAGgtagacGGAAATGTTCCTGCTATGGACAGTCTTCCAGCCATGACAGTAGCCAGGGCTCAGATGTGTCTCTCATAGCT

>Echtel_G44_pr_NW004558765_8812181_8813883

TCAGGGTCTCACACCCTCCAGGTGATGTCTGGTTGTGATTTGGGACCTGATGGGCATCTCCTCCGTGGGTACCGCCAATATGCCTATGATGGTGCCGACTACATCGCCTTGAATGAGGATTTGCGCTCCTGGACAGCGGCGGACATGGCTGCTCGGATCACCCAGCGTAAATGGGAGGAGGAAGGTGTGGCGGAGCTCCGAAGGGCTTATGTGGAGGGTATCTGTGTGTACTGGCTCCAGAGACACCTGGCGAATGGAAAGGAGGTGCTGCAGAGAGCAGAGCCTCCAAAGACCCAGGTGACCCGCCACCACAATCGCCATGTCCCTGAGGGAGAGGTCACACTGAGGTGCTGGGCCCGGGGCTTCTACCCGGCGGACATCGTCCTGACCTGGCAGCGGGACGGCGAGGACCAGACCCAGGACATGGAGCTGGTGGAGACGAGGCCTGCGGGGGACGGGACCTTCCAGAAGTGGGCGGCTGTGGTGGTGCCCTCCGGGGAGGAGCAGAGATACACGTGCCGAGTGCAGCACGAGGGGCTGCCTGAGCCCCTCACCCTGACATGGGAACTGTCTTCCCAGTCCAGCATCCCCATTGTGGGAATAGTAGTTGGCCTGGTTCTCGCAGCTGTGATCATTGGAGCTGTGATTGGATATGTGATCCGGAGGAAGAAGTGCTCAGGTGAAAAAGGAGGGACCTACTCTCAAGCTGCAAGCATTAACAGACAGAGCTTGGATGTGTCTCTAATAGCT

>Echtel_G3_ps_NW004558765_8824946_8827269

TCTCACACCATCCAGAGGATGTATGGCTGCGACCTGGGTCCCAATGGGAGCCTGCTCAGCGGGTACGCACAGTACGCTTACGATGGCACCGACTACATCTCCCTGAATGAGGACCTGCGCTCCTGGACCGCAGCGGACATGGCCGCGCTCATCACCAAGAGCAAGTTTGAAGAGGCCCGATGGGCTGAGCACATGAAGGCCTATGCGGAGGGCAAGTGTGTGGATTCACTCCTCAGATACCTGGAGAACGGGAAGGAGACCCTGCAGAGAGCAGACCCTCCGAAGACCCAGGTGACCCACCACCAGGAAAGCCATGTCCCTGAGGGAGAGGTCACACTGAGGTGCTGGGCTCGGGGCTTCTACCCAGCGGACATCGTCCTGACCTGGCAGCGGGACGGCGAGGACCAGACCCAGGACATGGAGCTGGTGGAGATGAGGCCTGCGGGGGACGGGACCTTCCAGAAGTGGGCGGCTGTAGTGGtgccctccagggaggaggagagataCAAGTGCCTAGTGCAGCACTAGGGGCTGCCTGAGTTCCTCACCCTGAGATGGGAACTGCCTTCCCAGCGCACCATCCCCATTGTGGGAATAGTAGTTGGCCTGGTTCTCCTCACAGCTGTGGTCATTGGAGCTGTGGTTGGATTTGTGATCCGGAGGAAGAAGTGCTCAcatagaggtggggaaggagggagctaTGCTCAAGCTGCAGGTgagtatgggggaggggggGGCTCAGATGTGTCTCTCATAGCT

1. **African bush elephant *Loxodonta africana***

>Loaf_G5_NW003573558_1634320_1637339

ATAGTTTCCCAGATCCTCCTCTTCTTGCTCTCGGGGCCCCTGGCCCTGACCCAGGCCCGAGCGGGCTCCCACTCCCTGAGGTATTTCGACACCGTTGTGTCCCGGCCCGGCCGCGGGGAGCCCCGGTACATCGCCGTCGGCTACGTGGACGACACGGAGTTCGTGCGGTTCGACAGCGACGCCGTGAACCCGAGGGCGGAGCCGCGGGCGCCGTGGATGGAGCAGGAGGGGCCGGAGTATTGGGACCGGGAGACACGGAGCGCCAAGGGCACCGCACAGGCTTTCCGAGTGAGCCTGCGGAACGTGCGCGGCTACTACAACCAGAGCGACGCCGGGTCTCACACATTCCAGAGGATATATGGCTGTGAAGTGGGTCCTGACGGGCTCTTCCTCCGCGGGTACGCTCAGTACGCCTACGACGGCTCCGACTACATCACCCTGAGCCAGGACATGCGCTCCTGGACCGCGGCGGACACGACCGCTCAGATCACCCAGCGCAAGTGGGAGGCTGACAAGTATGCAGATCAGGTCAAGGCCTACTTGGAGGGTGCCTGTGTGGAGGGGCTCCGCAGATACCTGGAGAACGGGAAGGAGTCTCTGCAGCGTGCAGATCCCCCAAAGGCACATGTGACCAACCACCCCATCTCTGACCATGAGGTCATGCTGAGATGCTGGGCCCTGGGCTTCTACCCAGCTGAGATCACACTGACCTGGCAGCGGGACGGGGAGGACCAGACCCAGGACACAGAGCTTGTGGAGACTAGGCCTGCGGGGGATGGGACCTTCCAGAAGTGGGTGGCCATAGTGGTGCTCTCTGGAGAGAAGCAGAGATACACGTGTCGTGTGCAGCATGAGGGGCTGCCTAAGCCCCTCACCTTGAGATGGGAGCCACTCCCTCAGTCCACCATCCCCGTCATGGGAATAGTTGCTGGCCTGGTGCTCTTTGGAGCCCTGGCTTTTGGAGCTGTGGTGGCTGCAGTTGTGATCTTAAGGAAGAGGAGCTCAGGCGGAAAGGGAAGGAGCTCCACACAGACTGCAAATGGCGACAGTGCCCAGGGATCTGATATGTCTCTCATGACT

>Loaf_G18_NW003573558_1696617_1699690

ATGGCTTTCCAGATCCTCCTCCAGTTGCTCTCAGGGGCGTTGGCCCTGACCCAGACCCGGGCGGGCTCCCACTCCCTGAGGTATTTCGAGACCGCCGTGTCCCGGCCCGGCCTCGAGAAACCCCGCTTCATCGCCGTCGGCTACGTGGACAACACGCAGTTCGTGCGGTTCGACAGCGACGTTGCGAATCCGAGGATGGAGCCGCGGGCGCAGTGGGTGAAGCAGGAGGGGCCGGAGTATTGGGACCGGAACACATTGATAGCCAAGGACAACGCACAGGCACACCGAGTGAACCTGCGGACCCTGCGCCGCTACTACAACCAGAGCGACGCCGGGTCTCACACCATCCAGAGGATGTCTGGCTGTGAAATGGAGCCCGAGGGGTACCTCCTCCGCGGGTACACTCAGTATGCCTATGATGGCGCCGATTACATCGCCCTGAACGAGGACCTGAGCTCCTGGACGGCTGTGGCCACTGCCGCTCAGATCACCCAGCGCAAGTGGGAGGAGGCCGGCGTGGCCAAGAAAAAGAAGACCTACTTGGAAGATGTGTGTGTGAAGTGGCTCCACAGATACCTGGAGAACGGGAAGGAGTCTCTGCAGCGCGCAGATCCCCCAAAAGCACATGTGACCAACCGCCCCATCTCTGACCGTGCAGTCACACTGAGATGCTGGGCCCTGGGCTTCTACCCGGCTGAGATCACACTGACCTGGCAGCGGGACGGGGAGGACCAGACCCAGGACACAGAGCTTGTGGAGACTAGGCCTGCGGGGGACGGGACTTTCCAGAAGTGGGCGGCCATGGTGGTGCCCTCTGGAGAGGAGGACAGATACACGTGTTGTGTGCAGCACGAAGGGCTGCCTGAGCCTCTCATCCTGAGATGGGATCCACCCCCTCAGTCCACCATCCCCATCAAAGGAATTGCTGCTGGCCTGAAGCTCTTTGGTGTGGTGGTTGTAGTTGTGGCTGGAGCTGTGGTTGCTGCAGTCGTGATCTTGATGAAGATGAGAGGTAGAAAGGGAAGGAGCTACACTCAGGCAGCAAATGGCGACAGTGCTCAGGGCTCTGGTGTGTCTCTCATGGCT

>Loaf_G31_NW003573542_1981655_1984534

ATGGCGCCCCGGACCCTCCTCCTGCTGCTCTCGGGGGCCCTGGCCCTGAGCCAGACTCGGGCGGGCTCCCACTCCCTGAGGTATTTTCACACCGCCGTGTCCCGGCCCGGCCGCGGGGAGCCCCGGTACATCGAAGTCGGCTACGTGGATGACACGCAGTTCGTGCGGTTCGACAGCGACGCCGCGAACCCGAGGATGGAGCCGCGGGCGCCGTGGATGGAGCAGGAGGGGCCGGAGTATTGGGATCGGAACACACAGAACGCCAAGGGCCACGCACAGACTTTCCGAGCGAGCCTGCGGACCCTGCGCGGCTACTACAACCAGAGCGACGCCGGGTCAGGGTCTCACACTATCCGGTTGTTGTACGGCTGTGAAGTGGGTGCCGACGGGCGCCTCCTCCGCGGGTACCATCAGTATGCCTACGACGGCGCCGATTATCTGGCCCTGAACGAGGACCTGCGCTCCTGGACGGCGGCGGACACGGCAGCTCAGATCTCAAAGGGCAAATTCGAGGTGGTCAGGGTGGCAGAGTATCGGAGGGCCTACCTGGAGGGCGCATGCCTGCAGTGGCTGGGCAAATACCTGGAGAACGGGAAGGAGACGCTGCAGCGGGTAGACCCCCCAAAGACACACATGACCCACCACTGCATCTCTGACCATGAGGCCACACTGAAGTGTTGGGCCCTGGGCTTCCACCCAGCGGAGATCACCCTGACCTGGCAGCGGGATGGGGAGGACCAGACCCAGGACATGGAGCTTGTGGAGACCCGGCCTGCAGGAGACGGGACATTCCAGAAGTGGGCAGCCCTGGTGGTGCCCTCTGGAGAGGAACAGAGATACACATGCCGTGTGCAGCATGAGGGGCTGTCTGAGCCTGTGACCCTGCGATGGGAACTGTCTTCCCAGGCCAGCACCCCGAAAATAGAGGTCGTTGCTGGCCTCCTTCTCCTTGGAACTGTAGTCCTTGGAGTTGTGGTGGCTGGAGCTGTTGTGATGTGGAGGAAGAAGAACTCAGGTGGGAAAGGAGGGAGCTACACTCAAGGTTCAAGCAGCGACAGTGACCAGGGCTCTGATGTGTCTCTCACAGCTTCTAAAGGT

>Loaf_G50_NW003573542_2165286_2168192

ATGGCGCCCCGGACCCTCCTCCTGCTGCTCTCGGGGGCCCTGGCCCTGACCCAGACCGGGGCGGGCTCCCACTCCCTGCGGTATTTCGACACCACCGTGTCCCGGCCCGGCCGCGGGGAGCCCCGGTACATCTCCGTCGGCTACGTGGACGACACGCAGTTCGTGCGGTTCGACAGCGACGCCGCGAACCCGAGGATGGAGCCGCGGGCACCGTGGATTGGGCAGGAAGGGCCGGAGTATTGGGATCGGAACACACAGATCGCCAAGGACAGCGCACAGACTTCCCGAGAGAGCCTGCGGACCGCGCTCGGCTACTACAACCAGAGCGACGCCGGGTCTCACACCATCCAGTTGATGTACGGCTGTGAAGTGAGTCCCGACGGGCGCCTCCTCCACGGGTACCATCAGTATGCCTACGACGGCGCCGATTATCTGGCCCTGAACGAGGACCTGCGCTCCTGGACGGCGGCAGACAGGGCAGCTCAGATGTCAAAGCGAAAATTCGAGAATCGCGGGGTGGCGGAGCGTTGGAGGGCCTACCTGGAGGGCACATGCCTGCAGTGGCTGGGCAAATACCTGGAGAACGGGAAGGAGACGCTGCAGCGAGTAGACCCCCCAAAGACACACATGACCCACCACCGCATCTCTGACCGTGAGGCCACACTGAGGTGTTGGGCCCTGGGCTTCTACCCGGCGGAGATCACCCTGACCTGGCAGCGGGATGGGGAGGACCAGACCCAGGACATGGAGCTTGTGGAGACCCGGCCTGCGGGAGACGGGACATTCCAGAAGTGGGCAGCCCTGGTGGTCCCCACTGGAGAGGAACAGAAATACACATGCCGTGTGCAGCATGAGGGGCTGTCTGAGCCTGTCACCCTGCGATGGGAACTGTCTTCCCAGGCCAGCACCCCCAACATAGGGGTCGTTGCTGGCCTCCTTCTCCTTGGAGCTCTGGTCCTTGGAGTTGTGGTGGCTGGAGCTGTTTGGATGTGGAGGAAGAAGAACTCAGGTGGGAAAGGAGGGAGCTACACTCAAGGTTCAAGCAGCAACAGTGACCAGGGCTCAGATGTGTCTCTCACAGCTTCTAAAGGT

>Loaf_G51_NW003573542_2663540_2666499

ATGGCGCCCCGGACCCTCCTCCTGCTGCTCTTGGGGGCCCTGGCCCTGACCCAGACCCGGGCGGGCTCCCACTCCCTGAGGTATTTCAGCACCGCCGTGTCCCGGCCCGGCCGCGGGGAGCCCCGGTACATCTCCGTCGGCTACGTGGACGACACGCAGTTCGTGCGGTTCGACAGCGACGCCGCGAACCCAAGGGAGGAGCCGCGGGCGCCGTGGATGGAGCAGGAGGGGCCGGAGTATTGGGACCAGGAGACACAGATCTCGAAGGGCAACACACAGACTTCCCGAGTGAACCTGCGGACCCTGCGCGGCTACTACAACCAGAGCGACGCCGGGTCTCACACCATCCAGAGGATGTACGGCTGTGAAGTGGGGCCCGATGGACGCCTCCTCCGTGGCTATGAGCAGCACGCCTATGACGGCGCCGACTACATCGCCCTGAACGAGGACCTGAGCTCCTGGACCGCGGCGGACACGGCCGCTCAGATCACCCGGCACAAGTGGGAGGCTGAGAAGGTTGCAGATCGGATCAGGGCCTACGTAGAGGGCAGGTGCCAGGAGTACCTCCGAAGATACCTGGAGAACGGGAAGGAATCTCTGCAGCGCGCAGACCCCCCCAAAGCACATGTAACCCACCACCCCATCTCTGACCGTGAGGCCACGCTGAGGTGCTGGGCCCTGGGCTTCTACCCTGCGGAGATCACCCTGACCTGGAAGCGGGACGGAGAGGACCAGACCCAGGACATGGAGCTTGTGGAGACCAGGCCTGCAGGGGACGGAAACTTCCAGAAATGGGCAGCCGTGGTGGTGCCTTCTGGAGAGGAGCAGAGATACACGTGCCATGTGCAGCATGAGGGGCTGTCCAAGCCTCTCACCCTGAGATGGGAGTCCCGCTCTCAGTTCCCTGCCATCATCACGGGAGTCATTACTGCCCTGGTTCTCCTTGTGGTTGTTATGCTGGCTGGAGCTGCGATCTGGAAGAAGATGAGCTCAGGTGGAAAAGGACGGAAATATGCTCAGGCTGCAAGCAGCAACAGTGCCCAGGGCTCTGATACGTCTCTGATGGCT

>Loaf_G53_NW003573542_2065220_2068093

ATGGCGCCCCGGACCCTCCTCCTGCTGCTCTCGGGGGCCCTGGCCCTGACCCAGACCGGGGCGGGCTCCCACTCCCTGAGATATTTTCACACCGCCGTGTCCCGGCCCGGCCGCGGAGAGCCCCGCTTCATCTCCGTCGGCTACGTGGACGACACGGAGTTCGTGTGGTTCGACAGCGACGCCGCGAACCCGAGGATGGAGCCGCGGGCGCCGTGGATCGAGCAGGAGGGGCCGGAGTATTGGGACCAGGAGACACAGAACGCCAAGGTCACCGCTCAGAACTACCGAGTGAACCTGCGGACCCTGCGCGGCTACTACAACCAGAGCGACGCCGGGTCAGGGTCTCACACCCTCCAGAGGATGTATGGCTGTGAAGTGGGTCCCGACAGGCGACTTCTCCGCGGGTACCTTCAGTTTGCCTACGACGGCGCCGATTATCTGGCCCTGAACGAGGACTTGAGCTCCTGGACTGCGGCGGACAGGGCAGCTCAGATCTCAAAGGGCAAATTCGAGAAGGGCGGGGAGGCGGAGTATCGGAGGGCCTACCTGGAGGGCTCATGCGTGCAGTGGCTCGGCAAATACCTGGAGAAGGGGAAGGAGACGCTGCAGCGGGTAGACCCCCCAAAGACACTCATAACCCACCACCGCATATCTGACCGTGAGACCACATTGAGGTGTTGGGCCCTGGGCTTCTACCCAGCGGAGATCACCCTGACCTGGCAGTGGGATGGGGAGGACCAGACCCAGGACATGGAGCTTGTAGAGACCCGGCCTGCAGGAGACGGGACGTTCCAGAAGTGGGCAGCCCTGGTGGTGCCCTCTGGAGAGGAACAGAGATACACATGCCGTGTGCAGCATGAGGGGCTGTCTGAGCCTGTGACCCTGCGATGGGAACTGTCTTCCCAGGCCAGCACCCCCATCATAGGGGTCGTTGCTGGCCTCCTTCTGCTTGGAGCTGTGGTCCTTGGAGTTGTGGTGGCTGGAGCTGTTGTGATGTGGAGGAAGAAGAACTCAGGTGGGAAAGGAGGGAGCTACACTCAAGCTGCAAGCAGCGACAGTGCCCAGGGCTCTGATGTGTCTCTCGTAGCTTCTAAA

>Loaf_G56_NW003573542_1884698_1887799

ATGGCGCCCCGGACCCTCCTCCTGCTGCTCTCGGGGGCCCTGGCCCTGACCCAGACCCGGGCGGGTGAGTGCGGGGTCGGGGGGAAAACGGCCTCTGCGGAGGGAGGAGCGAGGGGACCGCTCGCCGGGGACGCAGGACCCTCGGGGAAGCCGCCACCGCCCAGAGCCTCCACCCGCCCCTACACCCCGGCCCTGATCCTCCGGTCACCCCATCTCGGTCCCCTCTCCCCTCTCCCCTCTAGGAAATCGGATGGGGGAGCCCAAAATTATCTTTATTTAATGAGGAAGGAGAAAAGAACCAACCTGGGGGCGGGGTCAGGGTCTCACACTATCCAGTTGATGTACGGCTGTGAAGTGGGTCCCGACGGGCGCCTCCTCCACGGGTACCATCAGTATGCCTACGACGGCGCCGATTACCTGGCCCTGAACGAGGACTTGAGCTCCTGGACGGCGGCGGACTCGGCAGCTCAGATCTCAAAGGGCAAATTCGAGAATCGCGGGGAGGCGGAGTATCGGAGGGCCTACCTGGAGGGCGCATGCCTGCAGTGGCTGGGCAAATACCTGGAGAACGGGAAGGAGACGCTGCAGCGAGTAGACCCCCCAAAGACACACATGACCCACCACCACATCTCTGACCGTGAGGCCACGCTGAGATGTTGGGCCCTGGGCTTCTACCCGGCGGAGATCACCCTGACCTGGCAGCGGGATGGGGAGGACCAGACCCAGGACATGGAGCTTGTGGAGACCCGGCCTGCAGGAGACGGGACGTTCCAGAAGTGGGCAGCCCTGATGGTGCCCTCTGGAGAGGAACAGAGATACACATGCCGGGTGCAGCATGAGGGGCTGTCTGAGCCTGTCACCCTGCGATGGGAACTGTCTTCCCAGGCCAGCACCCCCATCATAGGGGTCGTTGCTGGCCTCCTTCTGCTTGGAGCTGTGGTCCTTGGAGTTGTGGTGGCTGGAGCTGTTGTGATGTGGAGGAAGAAGAACTCAGGTGGGAAAGGAGGGAGCTACACTCAAGCTGCAAGCAGCGACAGTGCCCAGGGCTCTGATGTGTCGCTCACAGCTTCTAAAGGT

>Loaf_G33_ps_NW003573542_2572929_2576300

ATGGCGCCCCGGACCCTCCTCCTGCTGCTCTCGGGGGCCCTGGCCCTGACCCAGGCCCGGGCGGGCTCCCACTCCCTGAGGTATTTTCACACCGCTGTGTCCCGGCCCGGCCTCGGGGAGCCCCGCTTCATCGCCGTCGGCTATGTGGACGGACACTCAGCGACGCGCCCGGAATCCGAGGGCAGAGCCGGCGGGCGGCCGTGGGCTGAGCAGGAGGGGCCGGAG!!GCATTGGGGAAGAGCTGACGCGGAGAGCCAAGTACATCTCACTGACTTCCCCGAGTGGCGCCTGCAGTCTCTGCGCGACTACTACAACCACAGCGACGCCGGAGGGTCTCACACATTCCAGAGGATGTTTGGCTGTGAAGTGGACTCCAAGGGACTCTTCCTCCGCGGGCGCTATCAGTACGCCTATGATGGAGCCGATTACATCTCCCTGAACGAGGACCTGCGCTCCTGGACGGCAGCGGACACAGTCGCTCAGATCACCCAGCGCCAGTGGATAGAGGCCGCTGTATCCGGGCATAAGAAGGCCTTCCTGGAGGGCTTGTGTGTGGAATGGCTCCGCAGATACCTGGAGAATGGGAAGGAGTCTCTGCAGCGGGCAGACCCCCCCAAAACACATGTGACCCACCACCTCATCTCTGAACGTGAGGCCACGCTGAGGTGCTGGGCCCTGGGCTTCTACCCTGCGGAGATCACCCTGACCTGGCAGCGAAATGGGGAGGACCAGACCCAGGACATGGAGCTTGTGGAGACCAGACCTACGGGGGATGGGACCTTCCAGAAGTGGGCGGCCATAGTGGTGCCCTTTGGACAGGAGCAGAGATACACGTGCCATGTGCAGCATGATGGGCTGCCCAAGCCTCTCACCTTGAGATGGGAGTCCCATTCTCAGTACCCCGCCATCATCATGGGAGTCATTGCTGGCCTGATTCTCCTTATGGTCATTGTAGCTGTGGTGGCTGGAGCTGTGATCTGGAGGAAGAAACAGTCAGGTAGAAAAGGAGGGAATTATGCTCAGGCTGGAAGCAGCAAAGGTGCCCAGGGCTGTGATGTGTCTCTTATGGCTTCTAAAGGT

>Loaf_G65_NW003573652_104108_107210

ATGGTTTCCCAGATCCTCCTCTTCTTGCTCTTAGGGTCCCTGTCCCTGACCCAGATCCAGGCGGGCTCCCACTCCCTGAGCCATTTCTTCACCGCCGTGTCCCGGGCCGGCGGCGTGGAGCCCCGCGTCATCGCCGTCGGCTACGTGGACGACACGCAGTTCGGACACTTTGACAGCGACGCCGCGAATCCCAGAGCGGAGCCGCGGACGCCGTGGGCGGAGCAGGACGAGCAGGAGCACTGGGAAGAGCTAACCCGTAGAGCCAAGGGCGTCGCACAGGATTTGCGAGTGGACCTGTGGACCCTGCGCGGCTATTACAACCAGAGCGACTCCGGTTCTCACACCATCCAGTGGCTGTCTGCCTGCGAGGTGAGGTCGGACGGGCGCTTCCTCCGCGGGTACCGTCGGATCGCCTACGACGGAACCGATTACATTGCCCTGAACGAGGACCTGCGCTCCTGGACGGCGGCGGACGCCGCGGCTCAGATCACCCGGCGCCAGTGGGAGGAGGCCGGTGTGGCGGAGCTTGTCAGAGCCTACCTGGAGGAGGAGTGTGTGGAGTGGCTGCATAGATACCTGGAGAAAGGGAAGGAGACGCTGCTGCGAGCAGATCCCCCAAAGGCTTATGTGACCCGCCACCCCATCTCTGACCATGAGGTCACGCTGAGGTGCTGGGCCCTGGGCTTCTATCCTGCGGAGATCATACTGACCTGGCAGCGAGATGGAGAGGACCAGACCCAGGACACAGAGCTTGTGGAAACCAGGCCTGCGGGGGATGGGAACTTCCAGAAGTGGGCCTCCCTGGCGGTACCCTCTGGAGAGGAGCAGAGATACGTGTGCCACGTGCAGCACGAGGGGCTATCTGAGCCCCTCACCCTGAAATGGGAGGTGCCTCCTCAGCCCACCACTTCCATCCTGGGAATTGTTGTTGGCCTGGTTCTCCTAGGAGCTGTGGTCACTGGAGTTGTGATCTGGTTAAAGAAGAGCTCAGGTAGGGGAGGGGCTGGGGTGGAAAAAGAAGGAAGTGGTAACAGTGCCCAGGGGTCTGATGTGCCTCTCACAGATTCTACAGGT

>Loaf_G11_NW003573652_176313_179777

ATGGCTTCCCAGGTCCTTCTCTTCTTGCTTTCGGGGTCCCTGGCCCTGACCCAGACCCAGGCGGGCTCCCACTCCCTGAGCCATTTCTTCACCGCCGTGTCCCGGCCCGGCGGCGGGGAGCCCCGCATCATCGCCGTTGGCTACGTGGACGACACGCAGTGGGGACGCTTTGACAGCGACGCCGCGAATCCCAGAGCGGAGCCGCGGACGTGGTGGATGGAGGAGGTGGAGCAGGAGCACTTGGAAGAGCTGACCCGCAGAGCCAAGGGCTTTGCACGGGATTTGCGAGTGCACCTGTGGACCCTGCGCGGCTACTACAACCAGAGCGACTCCAGTTCTCACACCATCCAGGCGCTGTCTGCCTGCGACGTAAGGTCGGACGGGCGCTTCCTCCGGGGGTACCGTCAGATGGCCTACGATGGCACCGATTACATTGCCCTGAACGAGGACCTGCGCTCCTGGACGGCGGCGGACGCCGCGGCTCAGATCACCCGGCGGGAGTGGGAGGAGGCCGGTGAGGCGGAGCTCGCCAGAGCCTTCCTGGAGGGGGAGTGTGTGGAGTGGCTGCATAGATACCTGGAGAAAGGGAAGGAGACCCTGCTGCGAGCAGATCCCCCAAAGGCATATGTGACCCACCACCCCATCTCTGATCGCGAGGTCACGCTGAGGTGCTGGGCCCTGGGCTTCTACCCTGTGGAGATCACACTGACCTGGCAGCGAGATGGAGAGGACCAGACCCAGGACACAGAGCTTGTGGAGACCAGGCCTGCGGGGGACGGGACCTTCCAGAAGTGGGCCGCCCTGGTAGTACCCTCTGGAGAGGAGCAGAGATACGTGTGCCGTGTGCAGCACGAGGGGCTGTCTGAGCCCCTCACCCTGAGATGGGAAGTGCCTCCTCATCCCACCACTTCCATCCTGGGAATTGTTGCTGGCCTGGTTCTCCTAGGAGCTGTGGTCACTGGCGTTGTAATCTGGATAAAGAAGAGCTCAGGTAGGGGAGGGGCTGGGGTGGAAAAAGAAGGAAGTGGTAACAGTGCCCAGGGCTCTGAAGTGTCTTTCACAGATTCTAAAGGT

>Loaf_G39_ps_NW003573652_16447_19326

ATGGCTTCCCAGATCCTCCTCTTGTTGCTCTCCGGGACCCTGCCCCTGACCCAGACCCGGGCGGGCTCCCACTCCCTGAACTATTTCTTCACCGCCGTGTCCCGGCCCGGCGTCGGGGAGCCCCGCGTCATC!!GGTCATCGCCGTCGGCTACGTGGATGACACGCAGTTCGGACACTTTGACAGCGACGCCGCGAATCCGGGGTCGGAGCCGCGGGCTCTTTGGGTGGTGCAGGAGGGGCCGGAGCATTGGGCAGAGCTGACGCGCAGAGCCAAGGACATCGCACAGGCTCTCCGAGTGGGCCTGCAGGCCTTGCGCGGCTACTACAACCACAGCGACACCAGTTCTCACACCATCCAGGCGCTGTCTGCCTGCGACGTAAGGTCGGACGGGCGCTTCCTCCGCGGGTGCCCTCGGATCGCCTACGACGGAACCGATTACATTGCCCTGAACGAGGACCTGCGCTCCTGGACGGCGGCGGACGGCGCGGCTCAGATCACCTGGCGTGAGCGCGCCGAGGCCGGTGAGGCGGAGCGCGTCAGAGCCTTCCTGGAGGGGGAGTGTGTGGAGTTGCTATGTAGATACCTGGAGAAAGGGAAGGAGACCCTGCTGCGAACAGATGCCCCAAAGGCTTATGTGACCCACCACCCCATCTCTGATCGCGAGGTCACGCTGAGGTGCTGGGCCCTGGGCTTCTATCCTGCGGAGATCACACTGACCTGGCAGCGAGATGGAGAGGACCAGACCCAGGACACAGAGCTTGTGGAGACCAGGCCTGCCGGGGATGGGACCTTCCAGAAGTGGGCCACCCTGGTGGTACCCTCTGGAGAGGAGCAGAGATATGTGTGCCGTGTGCAGCACAAGGGGCTCTCTAAGCCCCTCACCCCGAGATGGGAGATGCTTCCTCATCCCACCACTTCCATCCTGGGAGTTGTTGCTGGCCTGGTTCTCCTAGGAGCTGTGGTCACTGGAGTTGTGATCTGGATAAAGAAGAGCTCAGGTAGGGGAGGGGCTGGGATAATTCTGGTGATGAGAGCTGATTCTCAGCAGTCACAGGGCTCTGATGTGTCTCTCACAGATTCTAAA

>Loaf_G80_NW003573570_148406_151443

ATGGCTTCCCAGGTCCTTCTCTTCTTGCTTTCCGGGTCCCTGGCCCTGACCCAGACCCAGGCGGGCTCCCACTCCCTGAGCCATTTCTTCACCGCCGTGTCCCGGCCCGGCGGCGGGGAGCCCCGCGTCATCGCCGTCGGCTACGTGGACGACACGCAATTGGGACGCTTTGACAGCGACGCCGCAAATCCCAGAGCGGAGCCGCGGACGTGGTGGATGGAGCAGGAGGAGCAGGAGCACTTGGAAGAGCTGACCCGCAGAGCCAAGGGCGTCGCACAGAATTTGCGAGTGCACCTGTGGACCCTGCGCGGCTACTACAACCAGAGCGCCTCCGGTTCTCACACCATCCAGTGGCTGTCTGCCTGTGACCTAAGGTCGGACGGGCGCTTCCTCCGGGGGTACCGTCAGATGGCCTACGATGGCACCGATTACATTGCCCTGAACGAGGACCTGCGCTCCTGGACGGCGGCGGACGCCGCGGCTCAGATCACCCAGCGGGAGTGGGAGGAGGCCGGTAGGGCGGAGCGAGTCAGAGCCTTCCTGGAGGGGGACTGTGTGGAGTGGCTGTACAGATACCTGGAGAAAGGGAAGGAGACGCTGCTGCGAGCAGATCCCCCAAAGGCATATGTGACCCACCACCCCATCTCTGATCACGAGGTCACGCTGAGGTGCTGGGCCCTGGGCTTCTACCCTGTGGAGATCACACTGACCTGGCAGCGAGATGGAGAGGACCAGACCCAGGACACAGAGCTTGTGGAGACCAGGCCTGCGGGGGACGGGACCTTCCAGAAGTGGGCTGCCCTGGTAGTACCCTCTGGAGAGGAGCAGAGATACGTGTGCCGTGTGCAGCACGAGGGGCTGTCTGAGCCCCTCACCCTGAGATGGGAAGTGCCTCCTCAGCCCACCACTTCCATCCTGGGAATTGTTGCTGGCCTGGTTCTCCTAGGAGCTGTGGTCACTGGCGTTGTAATCTGGATAAAGAAGAGCTCAGGTAGGGGAGGGGCTGGGATCCTGTTTTGTTCTTCCCCAGGTGGTAACAGTGCCCAGGGCTCTGATGTGTCTTTCACAGATTCTAAAGGT

>Loaf_G28_NW003573570_257410_260481

ATGGCGTCCCAGATCCTCCTCTTCTTGCTCTCGGGGTCCCTGGCCCTGACCCAGACCCAGGCGGGCTCCCACTCCCTGAGCCATTTCTTCACCGCCGTGTCCCGGCCCGGCGGAGGGGAGCCCCGCGTCATCGCCGTCGCCTACGTGGACGACACGCAATTGGGACGCTTTGACAGCGACGCCGCGAATCCCAGAGCGGAGCCGCGGACGTGGTGGATGGAGCAGGAGGAGCAGGAGCACTTGGAAGAGCTGACGCGCAGAGCCAAGGGCGTCGCACAGGATTTGCGAGTGGACCTGTGGACCCTGCGCGGCTACTACAACCAGAGCGCCTCCGGTTCTCATACCATCCAGGCGCTGTCTGCCTGCGAGGTGAGGTCGGACGGGCGCTTCCTCCGCGGGTACCGTCAGATGGCCTACGATGGCACCGATTACATTGCCCTGAACGAGGACCTGCGCTCCTGGACGGCGGCGGACGCCGCGGCTCAGATCACCCGGCGGGAGTGGGAGGAGGCCGGTGTGACGAAGCTCGCCAGAGCCTTCCTGGAGGAGGACTGTTTGGAGTGGCTGCATAGATACCTGGAGAAAGGGAAGGAGACGCTGCTGCGAGCAGATCCCCCAAAGGCTTATGTGACCCACCACCCCATCTCTGACCGTGAGGTCACACTGAGGTGCTGGGCCCTGGGCTTCTACCCTGCGGAGATCACACTGACCTGGCAGCGAGATGGAGAGGACCAGACCCAGGACACAGAGCTTGTGGAGACCAGGCCTGCGGGGGACGGGACCTTCCAGAAGTGGGCCGCCCTGGTAGTACCCTCTGGAAAGGAGCAGAGATATGTGTGCCATGTGCAGCACGAGGGGCTGCCTGAGCCCCTCACCCTGAGATGGAAAGTGCCTCCTCATCCCACCACTTCCATCCTGGGAATTGTTGCTGGCCTGGTTCTCCTAGGAGCTGTGGTCACTGGCGTTGTGATCTGGATAAAGAAGAGCTCAGGTAGGGGAGGGGCTGGGTTTTGTTCTTCCCCAGGTGGTAACAGTGCCCAGGGCTCTGATGTGCCTCTCACAGATTCTAAAGGT

>Loaf_G32_NW003573780_90888_93778

ATGGCGCCCCGGACCCTCCTCCTGCTGCTCTTGGGGGCCCTGGCCCTGACCCAGACCCGGGCGGGCTCCCACTCCCTGAGGTATTTTCACACCGCCGTGTCCCGGCCCGGCCGCGGGGAGCCCCGCTTCATCTCCGTCGGCTACGTGGACGACACGGAGTTCGTGTGGTTCGACAGCGACGCAGCGAACCCGAGGATGGAGCCGCGGGCGCCGTGGATCGAGCAGGAGGGGCCGGAGTATTGGGACCAGGAGACACAGAACGCCAAGGTCACCGCACAGAACTACCGAGTGAACCTGCGGACCCTGCGCGGCTACTACAACCAGAGCGACGCCGGGTCAGGGTCTCACACCCTCCAGAGGATGTATGGCTGTGAAGTGGGTCCCGACGGGCGCCTCCTCCGCGGGTACCTTCAGTTTGCCTACGACGGCGCCGACTATCTGGCCCTGAACGAGGACTTGAGCTCCTGGACTGCGGCGGACAGGGCAGCTCAGATCTCAAAGGGCAAATTCGAGAAGGGCGGGGAGGCGGAGTATCGGAGGGCCTACCTGGAGGGCTCATGCGTGCAGTGGCTGAGCAAATACCTGGAGAAGGGGAAGGAGACGCTGCAGCGGGTAGATCCCCCAAAGACACTCATGACCTACCACCGCATCTCTGACCATGAGACCACACTGAAGTGTTGGGCCCTGGGCTTCTACCCGGCGGAGATCACCCTGACCTGGCAGCGGGATGGGGAGGACCAGACCCAGGACATGGAGCTTGTGGAGACCCGGCCTGCAGGAGACGGGACGTTCCAGAAGTGGGCAGCCCTGGTGGTGCCCTCTGGAGAGGAACAGAAATACACGTGCCGTGTGCAGCATGAGGGGCTGTCTGAGCCTGTCACCCTGCGATGGGAACTGTCTTCCCAGGCCAGCACCCCCATCATAGGGGTTGTTGCTGGCCTCCTTCTCCTTGGAGCTGTGGTCCTTGGAGTTGTGGTGGCTGGAGCTGTTGTGATGTGGAGGAAGAAGAACTCAGGTGGGAAAGGAGGGAGCTACACTCAAGGTTCAAGCAGCAACAGTGACCAGGGCTCTGATGTCTCTCTCATAGCTTCTAAA

>Loaf_G6_ps_NW003573555_731105_734663

ATGGTGCCCCAGGCCCTCCTCCTGCTGTTCTCGTGGACCTTGGCCCAGGCCCAGGCAGGCTCCCATTCCCTGGGGTATTTCGACACAGCCGTGTCCCGGCCTAACCGCGGGGAGCCGCGATACATCGCCGTCGGTTACGTGGACGACACGCAGTTCGTGCGGTTCGACAGCAAGGCCGCGAATCCGAGGGAGGAGCCGCGGGCGCCGTGGATAGAGCACGAGGGGCCGGAGTACTGGAACCCCAACACGCGGGCGGGT!!CTACAGTGCCTCTGCACAATCCTTTCGTGTGGGACTGCGGAGCCTGCGCAGCTACTACAACCAGAGCTACGCGGCAGGGTCTCACACCTTGCAGAGGATGTAGGGCTGCGAAGTGGGGCCGGATGGACGCTTCATCCGTGGGTTCTACGGGCTTGCCTATGACGGTGCGGATTATATCGCCCTGAGTGAAGACCTGCGCTCCTGGACAGCGGCGGACATGGCCGCTCAGATCACCCAGCGCAAGTGGGAGGAGGCCGGTAATGAAAAGCACGTGGCGATTTACCTGAAGGAGGAATGCGTGGAGTCTCTCCGCAGATACCTGGAAAACGGGAAGGAGACGCTGCAGCGCTCAGTGCCTCCAAAGACACACATGACCAGCCATCACATCTCTGACCATGAGGTCACACTGAGGTGCTGGGCCCTGAGCTTCTACCCAGCTGAGATCACCCTGACCTGGCAGTGGGACGGGGAGGACCAGACCCAGGACATGGAGCTTGTGGAGACCAGGCCTGCGGGCGATGGGACCTCCCAGAAGTGCGCGGCTGTGGTGGTGCCCTCTGGAAAGGAGCAGAGATACCTGGGCCATGTGCAGCACGAGGGGCTGCCTGATCCCGTCACTCTGAAATTGGAACTGCCTTCCCAGCCCAGCACCCCTATCCTGGCTGTCCTTGGAGTTGCGGTCGTTGGAGTTGTGGCTGGAGCTTTTATGATCTACAGGAAGAAGAGCTCAGGTAGAGATGGGGATAGCACCTCGTTTTTCTTATTTCACTGGGGGTTTCAAGTCCCAGATAGGAAATTGCCATCCTGCCTCATT

>Loaf_G25_ps_NW003573555_1808443_1813952

ATGGCACCCCGGACCCTCCTCCTGCTGCTCTCAGGGACCCTGGCCCAGACCCGGGGCGGGGATGGGGGCTCCCACTCCCTGAGGTATTTCGACACAGCCGTGTCCCGGCCCAACCACGAGGAGCCCCGGTACATCTCCGTCGGCTACGTGGACGACACGCAGTTCGTGGGGTTCGACGGCGACGCCGCGAATCCGAGGGAGGAGCCACGGGCGCCGTGGATGGAGCAGGAGGGGCCGGAGTATTGGGACCTTAACGCGCGGGTCTGCAAGGTTTCTGCGCAATCCTTTGGTGTGGGTCAGCGGAACCTGCGCGGCTACTACAACCTTAAGGAGTCTGGGCGGGGCCAGGGTCTCACACAGAGAATGTATGGCTGTGAATTGGGGTCAGATGGACGCCTCCTCCGTGGATTCTATCAACTGGCCTACGAGGGTGCCGATTACATCGCCCTGAATGAGGACATGGGCTCCTGGACAGCAGCGGACATGGCCGCTCGGATCACCCATCGCAAGTGGGAGGAGGCCAAAGAGCAATAGCACTTGAGGGTCCACTTTAAGGGAGAGTGCCTGGAGTGGCTCCGTAGGTACCTGGAGAAAGGGAAAGAGACTGCATCAGAGACAGTGCCTCCAAAGACACATGTGACTGATCACCACATCTCTGACCGTGAGGTCACACTGAGGTGCTGGGCCCTGGGCTTCTACCCAGCCGAAATCACCCTGACCTGACAAAGGGACGGTGAGGACCAGACTTA!GGATACAGAGACCTGGAAACTAGTCCTGCAGGGGACGGGACCTTCCAGAAGTGGGTGGCCGTGGTGGTGCCCTCTGGAGAGGAGCAGAGATACTTGTGCCATGTGCAGCATGATGGGCTGCCTGAGCCCATCACCCTGAGATGGGAACTGTCTTCCCGGCCCATCACCCCCAACATGGAAATTGTTGCTGTCCTTGTTCTGGGAATTGTTGCTGTCCTGGTTCTCCTTGGAGTTGTGGCTAGAGCTGTGATCTACAGGAAGAAGAGCTCAGGTGAAAGAGAAGGGAGCTACACTCAAGCTTCAAATGGTGACAGTGTCCAGGGCTCTGATGACTTGAGCTGCTCCAGAAACCCTGTG

>Loaf_G74_ps_NW003573528_1098608_1101402

ATGGTGCCGGGGACCCTCCTGCTGCTGCTCTCAGGTGCCCTGACCCTGACCCAGATCCGGGCTGGCTCCCACTCCCTGAGGTATTTCCACACCGCTATATCCCGGCCCGACCACGGGGAGCCCCGGTACATCGAAGTCGGCTACGTGGACAACACACAGTTCGTGCGGATCGACAGCGACGCCGCGCATCCGCGGACGGAGCCGCGGGCGCCATGGATCGAACACGTGGGGCCGGGGTATTGGGAGCAGCAGACACGGAGAGCGAAGGGCAGTGCACCATCTTTCCGAGGGAACTTGCTGACCTTGCGCAGCTACTACAACCAGAGCGACGCCGTAGGGCCTCACACCATCCAAGTGGTATTTGGCTGTGAGGTGGGTCCAGATGGGCGCCTCTTCCATCCGTACAATCGGCAGGCCTACGAGGGAGCTGATTACCTCGCCCTGAACGGGGACCTGCGCTTCTGGACCGCGGCGGACACGGCTGCTCAGATCACTCAGCCCAAGTGGGAGGCTGCTGGGTTTGTAGATCAGGTCAAGGCCTACCTGGAGGATACGTGTGTGGAGTGGCTGCACAGATAGTTGGAAAGCGGGAAAGAGACACTGCAGCGCACAGACCTCTCAAAGACACACGTGACTCACCACCCCATCTCTGACTGTGAGGCCACACTGAGGTGCTGGGCCCTGGGATTCTACCCAGCGGAGATCACCCTGATCTGGCAGCGGGATGGAGAGGACCAGACCCAGGACATGGAGCTTTTAGAGACCAGGCCTGCAGGGGACGGGACCTTCCAGAAGTGGGCAGCTATGGTGGTGCCCTCTGGACAGGAACAGAGATACACGTGCTATGTGCAGCATGA!GGGCTGCCTGAGCCCCTCACCCTGAGATGGGAGTCCCGTTCTCAGTCCCCCGCCCTCATTATGGGAATCATTGCTGGCCTGGCTCTCCTTGGAGCTGTGGTGGCTGGAGCTGTGATCTGGAGGAAGAAGAGCTCAGGTGGAGAAGGAGGGAACTATGCTCAGGCTGCAAGCAGTGACAGTGCC

>Loaf_G82_ps_NW003573804_56183_59168

ATGGCGCCCCGGGCCCTCCTCCTGCTGCTCTCGGGGGCCCTGGCCCTGACCCAGACCCGGGCGGGCTCCCACTCCCTGAGGTATTTCGACACCACCGTGTCCCGGCCCGGCGGCGGGGAGCCCCGCTTCATCGAAGTCGGCTACGTGGACGACACGCAGTTCGTGCGGTTCGACAGCGACGCCGCGAACCCGAGGATGGAGCCGCGGGCGCCGTGGATCGAGCAGGAGGGGCCGGAGTATTGGGATCGGAACACACAGATCGCCAAGGACAACGCACAGACTTCCCGAGTGAGCCTGCGGACCGCGCTGC!!GGCTACTACAACCAGAGCGACGCCGAGGGGTCTCACACCATCCAGGTGATGTACGGCTGTGAAGTGGGGCCCGACGGGCGCCTCCTCCGTGGCTATGGGCAGCACGCCTACGACGGCGCCGACTACATCGCCCTGAACGAGGACCTGAGCTCCTGGACGGCGGCGGACACGGCAGCTCAGATCACCCAGCGCAAGTGGGAGGCTGAGAAGTATGCAGATCAGTTCAGGGCCTACGTGGAGGGCAGGTGCCAGGAGTCGCTCCGCAGATACCTGGAGAACGGGAAGGAGTCTCTGCAGCGCGCAGACCCCCCCAAAGCACACATAACCCACCACCCCATCTCTGACCGTGAGGCCACGCTGAGGTGCTGGGCCCTGGGCTTCTACCCTGCGGAGATCACCCTGACCTGGCAGCGGGACGGAGAGGACCAGACCCAGGACATGGAGCTTGTGGAGACCAGGCCTGCAGGGGACGGAACCTTCCAGAAGTGGGCAGCCATGGTGGTGCCCTCTGGAGAGGAGCAGAGATACACGTGCCATGTGCAGCATGAGGGGCTGCCCAAGCCTCTCACCCTGACATGGGAGTCCCGTTCTCAGTCCTTCGCCATCATCATGGGAATCATTGCTGGCCTGGTATTCCTTGTGGTCGTTGTAGCTGTGGTGGCTGCAGCGGTGATCTGGAGGAAGAAGAGCTCAGATGGAAAAGGAGGGAACTATGCTCAGGCCGCAAGCAGCAACAGTTCCCAGGGCTCTGATGTGTCTCTCATGGCT

>Loaf_G70_ps_NW003573558_1658378_1660342

GAGGGGGTCGGTGGTCTCAGCCGCTCCCCGCCCCCAGGCTCCCACTCCCTGAGGTATTTTCACACCGCCGTGTCCCTGCCCGGCCGCGGGAAGCCCGGGTACATCGCCGTCGGCTACGTGGACCACATGGAGTTCGTGCGGTTCGAGAGCGACGCAGCGAACCCGAGGGCGGAGCCGCGGGTGCCGTGGATAAAGCAGGAGGGGCCGGAGTATTGGGACCGGGAGACACGGAGCGCCAAGGGCACCGCACAGACTTACCGAGTGGACCTGCGGACCCTGCACGGCTACTACAACCAGAGCGACGCCGGTGATCTCACTCTCCAGAGGATGTCTTGCTGCGACTTGGGGCAAGACAGGCGCTTCCTCCTTAGGTGTGAGCAGCTAGCGTATGATAG!GCCAATTACCTTGCCCTGAACAAACACCTGCGCTCCTGGAGCGCGGCAGACACCTTGGCTCAGATCACCCAGCGCAATTTGGAGGAGGCCGGTATGGCGGACCACGTCAGGGCCTACGTGGAAGGGGAGTGCGTGCACTGGTGTCCCAAAGGAGGGGAGGAGAATGGGATTGAGTCTCTCAGCCTCCACACAGATCCCCCACAGATACATGTGACCCACCACCCAATCTCTGAACTTGACGTTGACTGTGATGTCATGTTGAGGTGTTGGGCCCTGGGCTTCTACCTGGTAGAGATCACTCTGGCCTGGCAGCAGGATGGGGTGGACCAGACCCAGGACACGGAACTTGTGAAAACCAGGACTGCAGGGGGTGTGTGGCATGATGGGCTGCCCAAGTCCCTCACCCTCAGATGGAAGCCACCTTCTCAGTCCAGCACCCCCATCATGGGAATTGTTTACCTGTTTCTCCTTGGATCTGTGATCGCTGGAGCTGGGGTGGCTGGAATTGTG

>Loaf_G79_ps_NW003573509_651327_652069**

CAGGTGTTGTCTGGCTGTGAAGTGGACCCCGTCGGGCGCCTCCTCCGCGGGTACCGTCAGTATGCCTATGATGGCGCCGATTATCTCGCCCTGAATGAGGACTTGCGCTTCTGGACGGTGGCGGACACGGCAGCTCAGATCTCAAAGGGCAAATTCAAGAAGGGCAGGGAGGCAGAGAATCAGAGGGCCTACCTGGAGGGCACATGCGTGCAGTGGCTGGGCAAATACCTGGAGAACGGGAAGGAGACGCTGCAGCGAGTAGACCCCCCAAAGACACACATGACCCACCATCGCATCTCTGACCCTGAGACCACGCTGAGATGTTGGGCCCTGGGCTTCTACCCGGTGGAGATCACCCTGACCTGGCAGCAGGATGGGGAGGACCAGACCCAGGACATGGAGCTTGTGGAGACCTAGCCTGCTGGAAATGGGACGTTCCAGAAGTGGGCAGCCCTGGTGGTACCCTCTGGAGAGGAACAGAGATACACATGCC!TGTGCAGCATGAGGGGCTGTCTGAGCCTGTCACCCTGTGATGGGAACTGTCTTCCCAGGCCAGCACCCCTATCACAGGGATCGTTGCTGACCTCCTTCTCCTTGGAGCTGTGGTCCTTGGAGTTGTGGTGGCTGGAGCTGTTGTGATGTGGAGGAAGAAAAACTCAAGTGGGAAAGGAGGGAGCTACACTCAAGCTGCAAGCAGCGACAGTGCCCAGGGCTCTGATGTGTCTCTCACAGCTTCTAAAGCG

>Loaf_G66_ps_NW003573542_33386_35310

CCAAAGACACACGTGACTGATCACCACATCTCTGACCGTGAGATCACACTGAGGTGCTGGGCCCTGGGCTTCTACCCAGCCGAAATCACCCTGACCTGGCAAAGGGACGGTGAGGACCAGACCCAGGACACAGAG!ACCTGGAAACCAGGCCTGCAGGGGACGGGACCTTCCAGAAGTGGGTGGCCGTGGTGGTGCCCTCTGGAGAGGAGCAGAGATACCCGTGCGGTGTGCAGCACGAGGGGCTGCCTAAGCCCATCACCCTGAGATGGGAACTGTCTTCCCGGCCCATCACCCCCAACATGGAAATTGTTGCTGTCCTTGTTCTGGGAATTGTTGCTGTCCTGGTTCTCCTGGGAGCTATGCCTGCCTCCTCCCCAATCCAATCTCTCACAGAATTCTTTCTTCCTGCAGGTGAAAGAGGAGGGAGCTACACTCAAGCTTCAAATGGTGACAGTGTCCAGGGATCTGATGTACAAAGAGATTCATCCAAGACC

>Loaf_G43_pr_NW003573652_147284_148774

GGGCTCTGGAATTACTGCTATGACAAGGAACCTTTCCTCTGCTATCACCCAGAGACCAAGAGATGGACAGCACCCGGTACCTTGGCATTGGAAATGAAGAAGACCTGGGATGCAGATAGGGATAAACACAAATATTACGGTCACCATGTGGAGGGAGATATTTGTAAAAGACTGCGGAGCTACCTGGACTTGGGGAGAGACTTCATGGGGAGAACAGTGCCCCCAGCAGTGAACGTGACCCTCAGTCAGGCCATGAAGGGCAAAGTCACCCTGACTTGCTGGGCTGCTGGCTTCTATCCCCAGAAAATCACACTGACC

>Loaf_G35_pr_NW003573570_507203_508521

GGCATCTGGGATTACTGCTACGATGGGGAGCCATTCCTCTCCTATGACCCGGGGACCCAGAGATGGACGGTTCCCCTGTCCTCAACCCAGACCTTGGCACTGGAAATCAAGAAGACATGGGATACAGATGGGGATAAACACAAATATTACCATCACCATGTGCAGGGAGATATTTGCAAAAGACTGCGGAGCTACTCAACCTCTGGCAGAAACTTCATGGAGAGGACAGTGCCCCCAGCAGTGAACGTGACTGTCAGTAAGGCCGTGAAAGGCAACGTCACCCTGATGTGCAGGGCTGCTGGCTTCTATCCTCGGAAAATCACACTGACCTGGCTTCAGGATGGGGAACCCCTAAGCACGGACTCCCAGAACCACGGGTGTATCCTGCCAGATGGAAATGGGACCTATGAGACCTGGGTGTCCACAAGGCTCCCCCAAGGAGAGGAGCAGAGGTACAGCTGCCAAGTGGAACACAGTGGGAAGAACGTTACACAGCCTGTGAGCAATGGTGGGTCCTCAGTACAAGCGAGTGTAATACCAATCGTTCTTGCTGCTGTTGCTGCTATTGTTTTTGTCCTTTGGCGGTACAAGAAGGAGAAA

>Loaf_G87_pr_NW003573880_70623_72711

TCAGGGTCTCACACCTTACAGAGGATACACGGCTGTGAAGTGGGTCCCGACGGGCTCTTCCTCCGCGGGTACGTTCAGTATGCCTACGACGGCGCCGACTACATCACCCTGAGCCAGGACATGCGCTCCTGGACCGCGGCGGACACGACCGCTCAGATCACCCAGCGCAAGTGGGAGGCTGACAAGGTTGCAGATCAGGAAAAGGCCTACTTGGAGGGTGCGTGTGTGGAGGGGCTCCGCAGATACCTGGAGAACGGGAAGGAGTCTCTGCAGCGTGCAGATCCCCCAAAGGCACTTGTGACCAACCACCCCATCTCTGACCATGAGGTCATGCTGAGATGCTGGGCCCTGGGCTTCTACCCGGCTGAGATCACACTGACATGGCAGCGGGACGGGGAGGACCAGACCCAGGACATGGAGCTTGTGGAGACTAGGCCTGCGGGGGACGGGACCTTCCAAAAGTGGGTGGCCATGGTGGTGCCCCCTGGAGAGGAGCAGAGATACACGTGTCGTGTGCAGCATGAGGGGCTGCCTAAGCCCCTCACCTTGAGATGGGAGCCACCCCCTCAGTCCACCATCCCCGTCATGGGAATAGTTGCTGGCCTGGTGCTCTTTGGAGCACTGGTTGTTGGAGCTGTGGTGGCTGCAGTTGTGATCTTGAGGAAGAGGAGCTCAGGTGGAAAGAGAAGGAGCTCCATGCAGACTGCAAATGGCGACAGTGCCCAGGGCTCTAATATATCTCTCATGGCT

>Loaf_G42_pr_NW003574726_247_2366

TCAGGGTCTCACACCATCCAGAGGATGTCCGGCTGTGAAGTGGGGCCCGACGGGCGCCTCCTCCGTGGCTACCAGCAGCACGCCTATGACGGCGCCGACTACATCGCCCTGAACGAGGACCTGAGCTCCTGGACCGCGGCGGACACGGCCGCTCAGATCACCCAGCGCAAGTGGGAGGCTGAGAAGGCTGCAGATCGGATCAGGGCCTACGTGGAGGGCAGGTGCCAGGAGTACCTCCGCAGATACCTGGAGAACGGGAAGGAATCTCTGCAGCGCGCAGACCCCCCCAAAGCACACATAACCCACCACCCCATCTCTGACCGTGAGGCCACGCTGCGGTGCTGGGCCCTGAGCTTCTATCCTGCGGAGATCACCCTGACCTGGCAGCGAGATGGAGAGGACCAGACCCAGGACATGGAGCTTGTGGAGACCAGGCCTGCAGGGGACGGAACCTTCCAGAAGTGGGCAGCCATGGTGGTGCCCTCTGGAGAGGAGCAGAGATACACGTGCCATGTGCAGCATGAGGGGCTGCCCAAGCCTCTCACCCTGACATGGGAGTCCCGTTCTCAGTCCCCTGCCATCATCGTGGTTATCATTGCTGGCCTGGTATTCCTTGTGGTCGTTGTAGCTCTGGTGGCTAGAGCTGTGATCTGGAGGAAGAAGAGCTCAGGTGGAAAAGGAGGGAACTATGCTCAGGCTGCAAGTAACAACAGTGCCCAGGGCTCTGATGTGTCTCTCATGGCT

>Loaf_G8_ps_NW003573555_851452_853478

TGGTTAAGAGATTGGCTGCTAACTGAAAGGGAGACAGACTCGTTTTCAGATTCACCATTAACACACGTGATCCACCACCCCATCTTTGACTGTGAGGCCACACTGAGGTTCTGGGCCCAGAGCTTCTACCTGGCAGAGATCATACTGACCTGGCAGCAGCACTGGGAGGATCAAACCCAAGACAAGGAGGTTGTGGAGACCATGCCTGCGTGGGACGGGACCTTCCAGAAATGGGGGGCCATGGTGTGTCCT!CTGGAGAGAAGCAGAAATATACACGTCGTGTGCAGCAGGAGAGGCTGCTGGAGCCCCTTACCCTGAGAAGGGTGGAGCCTCTTCTCAAGCCCACCATCCCCGTTGTGGGAATCCTTGCCAGCCTGGTTCTCCTTGGAGCTGTGGTAGCTGAAGTTCTGGTGGCTGGAGTTGTGATCTGGAGGAAGAAGGTCTCAGGTAGGGAAGGGAGTGGCTACCTTGCCCAACATAGAAACAAGGATAATAGCCTCTTTATGTTACAAGCAAGAAAGGTG

>Loaf_G10_ps_NW003573509_651327_652069

CAGGTGTTGTCTGGCTGTGAAGTGGACCCCGTCGGGCGCCTCCTCCGCGGGTACCGTCAGTATGCCTATGATGGCGCCGATTATCTCGCCCTGAATGAGGACTTGCGCTTCTGGACGGTGGCGGACACGGCAGCTCAGATCTCAAAGGGCAAATTCAAGAAGGGCAGGGAGGCAGAGAATCAGAGGGCCTACCTGGAGGGCACATGCGTGCAGTGGCTGGGCAAATACCTGGAGAACGGGAAGGAGACGCTGCAGCGAGTAGACCCCCCAAAGACACACATGACCCACCATCGCATCTCTGACCCTGAGACCACGCTGAGATGTTGGGCCCTGGGCTTCTACCCGGTGGAGATCACCCTGACCTGGCAGCAGGATGGGGAGGACCAGACCCAGGACATGGAGCTTGTGGAGACCTAGCCTGCTGGAAATGGGACGTTCCAGAAGTGGGCAGCCCTGGTGGTACCCTCTGGAGAGGAACAGAGATACACATGCC!TGTGCAGCATGAGGGGCTGTCTGAGCCTGTCACCCTGTGATGGGAACTGTCTTCCCAGGCCAGCACCCCTATCACAGGGATCGTTGCTGACCTCCTTCTCCTTGGAGCTGTGGTCCTTGGAGTTGTGGTGGCTGGAGCTGTTGTGATGTGGAGGAAGAAAAACTCAAGTGGGAAAGGAGGGAGCTACACTCAAGCTGCAAGCAGCGACAGTGCCCAGGGCTCTGATGTGTCTCTCACAGCTTCTAAAGCG

>Loaf_G16_ps_NW003573624_63348_65267

GCTGAGAGCGTCGGGGCCCACCCGGAAGTTGAGTGCCTGGAGTGGCTCCGCAGAAGAGTGACTTACCCAGAAAGGCCCTCCTTTCTCTCAGAAACAGTGCCTCCAAAGACACACATGACCGACCACTGCGTCTCTGACTGTAAGGTCACACTGAAGTGCTGGGCCCTGAGCTTCTACCTGGTGGCGATCACGCTGACCTGGCAGCAGAACGAGGAGGACCAGACCCAGGATGTGGAGCTTGTAGAGGCCAGGCCTGCAGGGGACAGGACCTTCCAGAAGTGGGCG!!GGCAGCCTTGGTGGTGCCCTCTGGAGAGGAGCAGAGATACACGTGCTGTGTGCAGCATAAGGGGCTGCCTGAGCCCCTCACACTGAGATGGGAACTGTCTTCCCAACCCACCACCCACATCATGGGAATCATTGCTGGCCTGATTCTTCTTGGAGCTGTGATTGTTGGGGGTGTGGCAGCTGGAGCTGTGGTCTGGAGGAAGAAGATCTCAGGTGGAAAAAGAGGAAGCTATGCTCAAGTTGCAAGCATTAACAGTGCCAAGGCTCTGATAATGTCTCTCATGGCTTCTAAAGGT

>Loaf_G63_pr_NW003573558_1775671_1777645

TCAGGTTCTCACACCATCCAGGCGCTGTCTATCTGCGAGGTAGGGTCGGACGGGCGCTTCCTCCGCGGGTACCGTCGGATCGTGAACGAGGACCGGCGCTCCTGGACGGCGGCGGCCGGCGCGGCTCAGATCACCCGGCGGGAGTGGGAGGAGGCCGGTGAGGCGGAGCGCGTCAGAGCCTTCCTGGAAGGAGAGTGTGTGGAGTGGCTGCATAGATACCTGGAGAAAGGGAAGGAGACGCTGCTGCGAGCAGATCCCCCAAAGGCATATGTGACCCACCACCCCATCTCTGATGGTGTGGTCACGCTGAAGTGCTGGGCCTCGGGCTTCTACCCTTCGGAGATCACCCTGACCTGGCAACGAGATGGGGAGGACCAGACCCAGGACACAGAGTTTGTGGAGACCAGGCCTGCAGGGGACGGGACCTTCCAGAAGTGGGCTGCCCTGGTAGTACCCTCTGGAGAGGAGCAGAGATATGTGTGCCTTGTGCAGCATGAGGGGCTGTCTGAGCCCCTCACCTTGAGATGGGAGGTGCCTCCTCAGCCCACCACTTCCATCCTGGGAATTGTTGCTGGCCTGGTTCTCCTAGGAGCTGTGGTCACTGGAGTTGTGATCTGGATAAAGAAGAGCTCAGGTGGAAGAAGAGGGACCTATGTTCAGACCCTTAGTGGTGACAGTGCCCAGGGCACTGATGTGCCTCTCACAGATTCTAAA

>Loaf_G68_pr_NW003573570_433024_435255

AGTACACCCCGGACCCTCCTCGTGTTGCTCTCGGGGGCACTGTTCCTCACCCAGACCAAGGCGGGCTCCCACTCCCTGAGGTATTTTCACACCGCCGTGTCCCGGCCCGGCCGCGGGGAGCCCCGCTTCATCGAAGTCGGCTATGTGGACGACACGCAGTTCGTGCGATTCGACAGCGACGCCGCGAATCCGAGGATGGAGCCGCGGGCGCGGTGGGTGGAGCGGGAGGGGCCGGAGCATTGGGACCAGGAGACACGGATCGCCAAGGACAACGCACAGAACTACCGAGTGAACCTGCGGACCCTGCGCGGCTACTACAACCAGAGCGACGCCGCAGGGTCTCACACCATTCAGGTGATGTTCGGCTGCGAAGTGGGGTCAGATGGTCGCCTTCTCCGCTGGTACAATCAGCATGCCTATGATGGCGCCGATTACATCGCCCTGAATGAGGACCTGCAATCCTGGACAGCGGCGGACACGGCCGCTCAGATTACGCAGCGCAAGTGGGAGGAGGCCGGTGTGGCGGACCACATCAGGGCCTACGTGGAGGGCAGGTGCCTGGAGTGGCTCCGCAGATACCTGGAAAACGGGAAGGAGACGCTGCGGCGAGCAGTGCCCCCAAGGATGCACATGACCCACCACCACATTTCTGACCATGAGGCCACACTGAAGTGCTGGGCCCTGGGCTTCTACCCAGCGGAGATCACCCTGACCTGGCAGCGGGATGGGGAGGACCAGACCCAGGACATAGAGCTTGTGGAGACCAGGCCTTCGGGGGATGGGACCTTCCAGAAGTGGGTGGTGGTGGTGGTGCCCTCTGGAGAGGAGCACAGATACACGTGCAATGTGCAGCATGAGGGGCTGCTGGAGCCCTTCACCCTGAGATGGGAAACATCTTCCCAGCCCACTAGCCTCGTCATGAGCATTGTTGCTGGCCTGGTTCCTGTAGCTGTGGTTGTT

>Loaf_G22_pr_NW003573432_62461351_62461602

!TCGGTTTCCCAGATCCTTCTCTCTTGCTCTCGGGGCCTCTGGCCCTGACCCAGACCGGAGCGGGCTCCCACTCCCTGAGGTATTTCGACACCGCTGTGTCCCGGCCCGGCCGCGGGGAGCCCCGCTTCATCGCCGTCGGCTACGTGGACGACACGGAGTTCATGCGGTTCGACAGCGACGCCGCGAAGCCGAGGGCGGAGCCGCGGGCGCCGTGGATGGAGCAGGAGGGGCCGGAGTATTGGGACCGGGAGACACAGAGAGTCCAGGGCCACGCACAGGCTTTGCGAGTGGGCCTGCGGAACGTGCGCGGCTACTACAACCAGAGCGACGCCGGGTCTCACACCTTCCAGAAGATATATGGCTGTGAAGTAGGTCCCGACGGGCTCTTCCTCCGCGGGTACGCTCAGTTTGCCTACGACGGCGCCGACTACATCACCCTGAGCCAGGACCTGAGCTCCTGGACCGCGGCGGACACGACCGCTCAGATCACGCAGCACAACTGGGAGGCTGGCAAGTTTGCAGATCAGGTCAAGGCCTACTTGGAGGGTGCGTGTGTGGAGGGGCTCCACAGATGCCTGGGGAACGGGAAGGAGTCTCTGCAGCGTGCA
